# Local translation of endosome-associated *lc3b* mRNA in axons contributes to endosomal clearance

**DOI:** 10.1038/s44319-026-00867-5

**Published:** 2026-07-17

**Authors:** Francesca Pappacena, Cinzia Caterino, Stefano De Pretis, Cristina Sironi, Alessandro Esposito, Giuliana Cesare, Michela Bruni, Jean-Michel Cioni

**Affiliations:** 1 IRCCS San Raffaele Scientific Institute, Division of Neuroscience, Milan, Italy; 2 IRCCS San Raffaele Scientific Institute, Center for Omics Sciences, Milan, Italy

**Keywords:** Membranes & Trafficking, Neuroscience, RNA Biology

## Abstract

RNA localization to organelles is emerging as a key mechanism for regulating protein expression at the subcellular level in neurons. Although certain transcripts associate with endosomes, the functional significance remains poorly understood. Using APEX-seq, we identify a broad set of mRNAs localized to endosomes. We focus on the autophagy-related *lc3b* mRNA and confirm its endosomal association in cultured cells and *Xenopus* neuronal axons. In axons, *lc3b* mRNA is translated at endosomes, where the resulting LC3B protein also colocalizes, suggesting a tight spatial coupling between transcript localization and protein function. Impairment of LC3B membrane insertion via expression of a mutant ATG7 leads to the accumulation of enlarged axonal endosomes. Moreover, RAB5 overactivation promotes the formation of dysfunctional endosomes in axons that are targeted and cleared by LC3B-mediated autophagy. Finally, chloroquine-induced damage to axonal endosomes triggers their targeting by LC3B in a translation-dependent manner. Collectively, our findings expand the catalog of endosome-associated transcripts and reveal a functional link between autophagy and endosomal turnover in axons.

## Synopsis

*lc3b* mRNA is associated with and translated on endosomes in axons. Moreover, LC3B promotes the clearance of damaged axonal endosomes, establishing a link between RNA localization and local organelle quality control.

- APEX-seq identifies a broad repertoire of endosome-associated mRNAs.
- Endosomes serve as translation sites for *lc3b* mRNA in axons.
- Disruption of LC3B function impairs endosomal turnover in axons.
- Endosome damage leads to recruitment of *lc3b* mRNA.

## Abstract (web summary)

*lc3b* mRNA is associated with and translated on endosomes in axons. Moreover, LC3B promotes the clearance of damaged axonal endosomes, establishing a link between RNA localization and local organelle quality control.

## Graphical abstract

**Figure:**
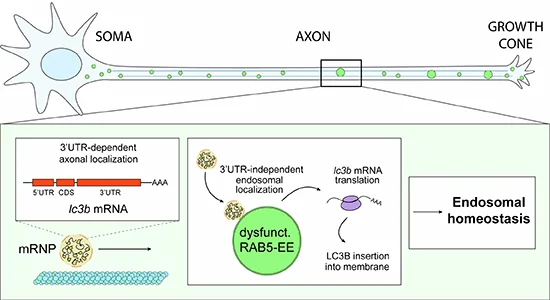

**Figure:**
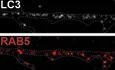

## Introduction

RNA localization enables spatial control of gene expression. This fundamental mechanism ensures that proteins are produced where they are needed, allowing cells to respond quickly to extracellular signals, developmental cues, and cellular stress (Das et al, 2021). In neurons, mRNA transport and localized translation within axons and dendrites enable rapid changes to the local proteome, supporting processes such as synaptic plasticity, axon maintenance, and memory consolidation (Holt et al, 2019). Importantly, disruptions in mRNA localization are linked to various neurological diseases (Fernandopulle et al, 2021), but the functions and mechanisms guiding mRNA targeting and translation in neuronal sub-compartments remain to be fully understood.

Recently, endosomes have emerged as hubs for mRNA localization and translation. Direct mRNA-endosome associations have been observed across cell types, including neurons (Chouaib et al, 2020, preprint: Popovic et al, 2020; Baumann et al, 2014; Cioni et al, 2019). The FERRY complex acts as a RAB5 effector on early endosomes (EEs), bringing a specific group of transcripts and ribosomes to their membrane (Schuhmacher et al, 2023). Additionally, in axons, lysosomes transport a selective subset of mRNAs (De Pace et al, 2024), and Annexin A11 facilitates this process by tethering RNA-binding protein-containing granules to lysosomal membranes (Liao et al, 2019). While the interplay between the endolysosomal system and mRNA regulation is increasingly recognized, a thorough profile of the mRNAs interacting with these organelles and the biological functions associated with these interactions has yet to be fully defined.

To address these questions, we analyzed the endosome-associated transcriptome by coupling proximity labeling with RNA sequencing. We then focused on the identified autophagy-related *lc3b* mRNA and showed that this transcript localizes to axons, where it is locally translated in association with endosomes, and the encoded protein contributes to endosomal homeostasis. This work expands our knowledge of the endosomal-associated transcriptome and links autophagy to the regulation of axonal endosomes.

## Results

### Characterization of the transcripts in proximity to endosomal membranes

**Figure 1:**
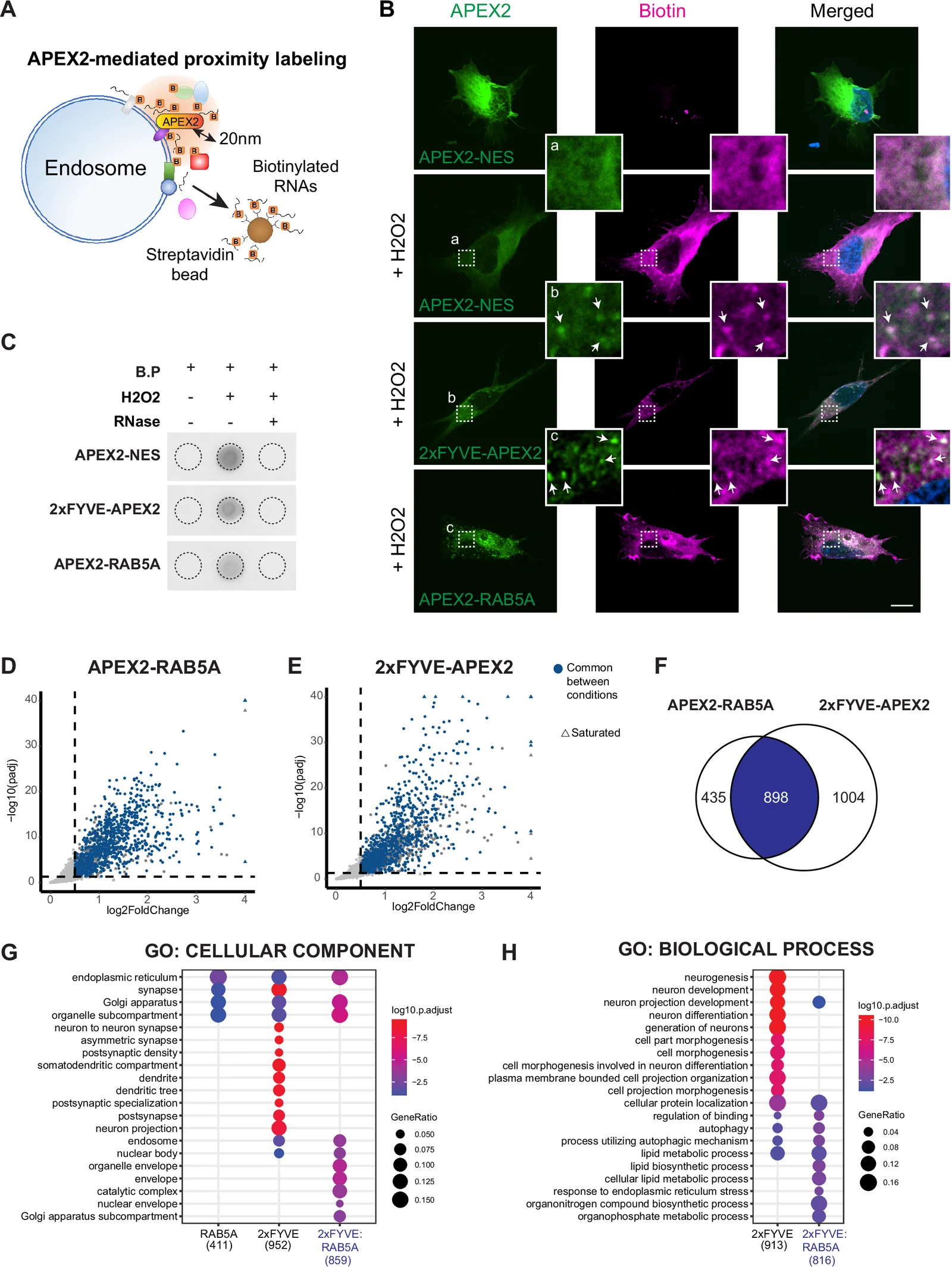
Identification of endosome-associated transcriptome by APEXseq. (**A**) Representation of APEX2-mediated proximity biotinylation and subsequent isolation of biotinylated RNAs. (**B**) IF performed on SH-SY5Y cells showing APEX2 construct localization (in green) and biotinylation activity (in magenta) in presence of H₂O₂. Arrows indicate biotinylation activity matching APEX2 localization. (**C**) Streptavidin-biotin RNA dot blot following APEX2 activation (B.P., Biotin Phenol, 3 exp). (**D**) Scatter plot showing mRNAs enriched following pulldown in APEX2-RAB5A line treated with or without H₂O₂ (4 exp). (**E**) Scatter plot showing mRNAs enriched following pulldown in 2xFYVE-APEX2 line treated with or without H₂O₂ (4 exp). (**F**) Venn diagram showing the number and overlap between the enriched mRNAs. (**G**, **H**) Gene Ontology (GO) enrichment analysis for Cellular Components and Biological Process of the identified transcripts. The number of transcripts analyzed is indicated for each condition. (**D**, **E**) Wald test. (**G**, **H**) Hypergeometric test. Scale bars: (**B**) 10 μm. Source data are available online for this figure.

Direct endosome-mRNA association has been reported for a few specific mRNAs, but many transcripts may engage in weak or transient associations that are difficult to capture by biochemical isolation of the organelle. To extend our knowledge of which mRNAs associate with endosomes, we employed APEX-seq, a technique that combines proximity labeling with RNA sequencing (Fig. 1A) (Fazal et al, 2019). To ensure sufficient starting material, we used human neuroblastoma SH-SY5Y cells as a neuron-like model. Labeling was restricted to endosomes by selecting two constructs previously used for proximity-based proteomic analysis. The first allows the expression of APEX2 fused to a double FYVE finger of Hrs (2xFYVE-APEX2) (Lobingier et al, 2017), a small zinc-binding domain that recognizes PI(3)P lipids enriched in EE membrane. The second allows the expression of APEX2 fused to the EE-associated small GTPase RAB5A (APEX2-RAB5A) (Del Olmo et al, 2019). Additionally, we used a cytoplasmic APEX2 construct (APEX2-NES) as a control. Both 2xFYVE-APEX2 and APEX2-RAB5A displayed punctate patterns within cells, whereas APEX2-NES showed a diffuse signal throughout the cytoplasm (Fig. 1B). As expected, immunofluorescence (IF) experiments revealed that biotinylation occurred only in the presence of biotin phenol (BP) and H₂O₂, with a pattern that mirrored APEX2 localization (Fig. 1B). To confirm that RNAs were biotinylated, we performed a dot blot assay on total RNA extracted following APEX2 activation. A positive streptavidin signal appeared only with BP and H₂O₂ and was RNase-sensitive, indicating efficient RNA biotinylation (Fig. 1C).

**Figure EV1:**
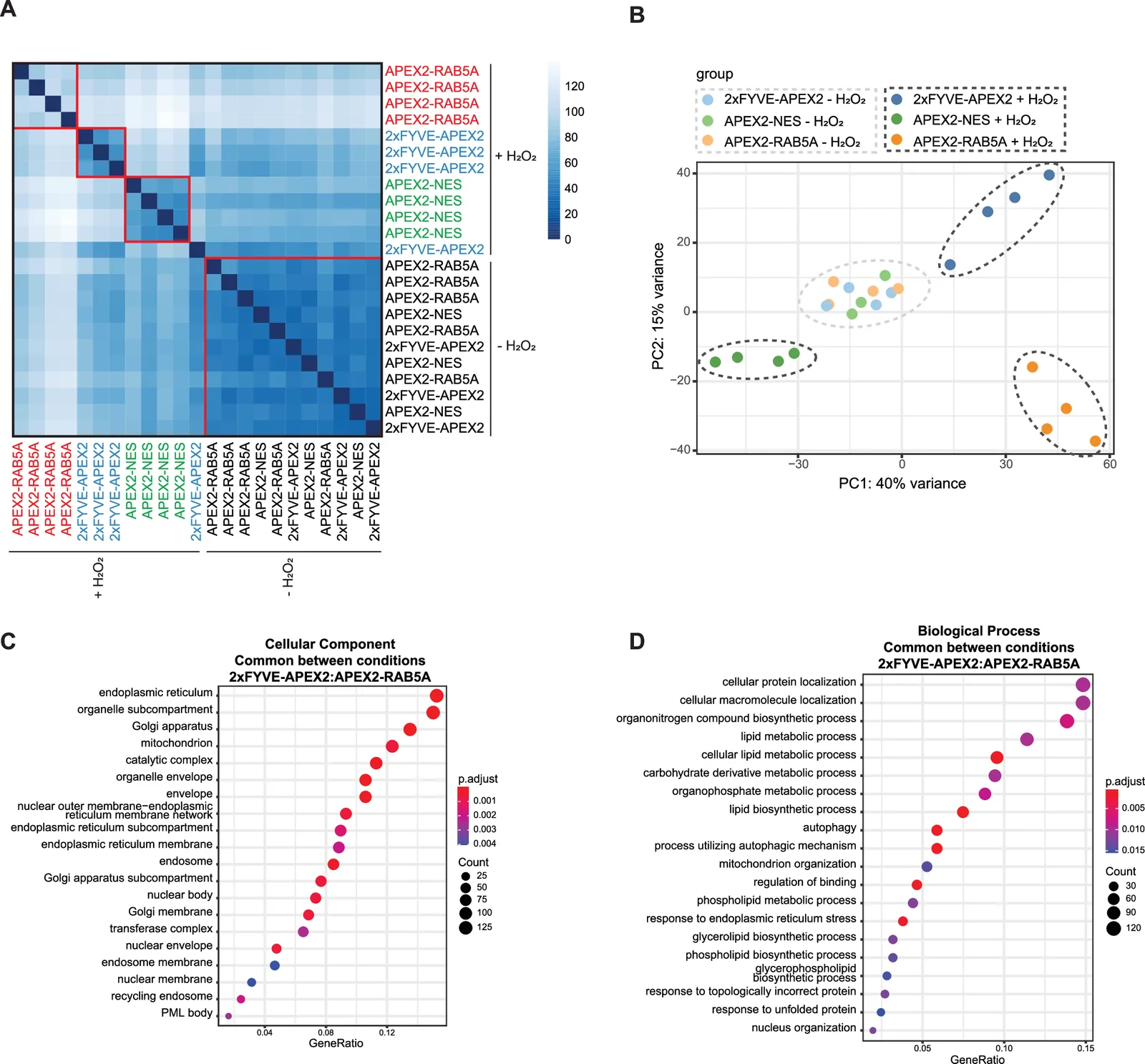
APEXseq identifies distinct transcriptomes in H₂O₂-treated and untreated samples. (**A**) Heatmap showing similarity and variance between RNA samples obtained from each APEX2 cell line treated with or without H₂O₂ in 4 independent biological replicates. Color scale indicates distances between samples. (**B**) Principal Component Analysis (PCA) showing the clustering of untreated samples from each APEX2 line together, and the separation between samples from different APEX2 cell lines treated with H₂O₂. (**C**) Gene Ontology Cellular Component analysis of mRNAs identified in both 2xFYVE-APEX2 and APEX2-RAB5A conditions, showing the top 20 enriched categories. (**D**) Gene Ontology Biological Process analysis of mRNAs identified in both 2xFYVE-APEX2 and APEX2-RAB5A conditions, showing the top 20 enriched categories. (**C**, **D**) Hypergeometric test.

To next identify transcripts biotinylated by APEX2, we performed streptavidin-based pulldown followed by RNA sequencing. Correlation plot of biological replicates revealed that the unbiotinylated samples (no H₂O₂) from all three conditions clustered together and were distinctly segregated from the biotinylated samples (Fig. EV1A,B). A comparative analysis was then performed to identify mRNAs enriched in biotinylated samples relative to all unbiotinylated samples combined (Dataset EV1). Only few mRNAs were significantly enriched in the APEX2-NES condition, suggesting that cytoplasmic APEX2 may weakly biotinylate a broad, heterogeneous set of mRNAs and/or that captured cytoplasmic mRNAs lack reproducibility across experiments. Conversely, we identified over a thousand significantly enriched transcripts in APEX2-RAB5A and 2xFYVE-APEX2 conditions (Fig. 1D,E). This abundance of recovered mRNAs was similar to the transcriptomes associated with other sub-compartments open to the cytosol (Fazal et al, 2019). Nevertheless, it is important to consider that our results may be influenced by the expression of APEX2 fusion proteins. While the overlap between the RAB5A- and 2xFYVE-associated transcriptomes supports their specificity (Fig. 1F), their differences also suggest occasional localization to distinct endosomal compartments (van der Beek et al, 2022).

Gene Ontology (GO) analyses of EE-enriched mRNAs for Cellular Component showed that a significant portion of the transcripts are related to specific neuronal compartments, including neuronal processes and the synaptic environment (Fig. 1G). By focusing on the mRNAs identified in both 2xFYVE-APEX2 and APEX2-RAB5A, several endosome-related mRNAs were present, supporting an enrichment for mRNAs encoding proteins that are directly localized on the organelle itself. Among them were regulators of endocytosis (e.g. *ap2a2*, *bin1*, *clta*, *epn2*) and endosomal trafficking (e.g. *rabep1*, *snx3*, *vps51*). Notably, the identification of *eea1* mRNA in our APEX2-RAB5A condition is consistent with findings showing its presence on EEs in 7 mammalian cell lines (preprint: Popovic et al, 2020).

Biological Process analysis further revealed the presence of a high number of mRNAs involved in neuronal development and differentiation in the 2xFYVE-APEX2 condition (Fig. 1H). Overlap between 2xFYVE-APEX2 and APEX2-RAB5A conditions revealed a substantial repertoire of mRNAs encoding regulators of lipid metabolism and membrane-remodeling factors (Figs. 1H and EV1D), raising the possibility that localized translation dynamically controls membrane identity and trafficking competence. Interestingly, we also found an enrichment for transcripts related to autophagy shared between the two conditions (Fig. 1H). The endosomal and autophagic pathways are tightly interconnected, sharing key molecular components and exhibiting strong functional interdependence (Birgisdottir and Johansen, 2020). Building on these connections, we decided to further investigate the relationship between these two pathways at the RNA level.

### *lc3b* mRNA localizes to RAB5-positive endosomes in neuronal-like cells and neuronal axons

**Figure 2:**
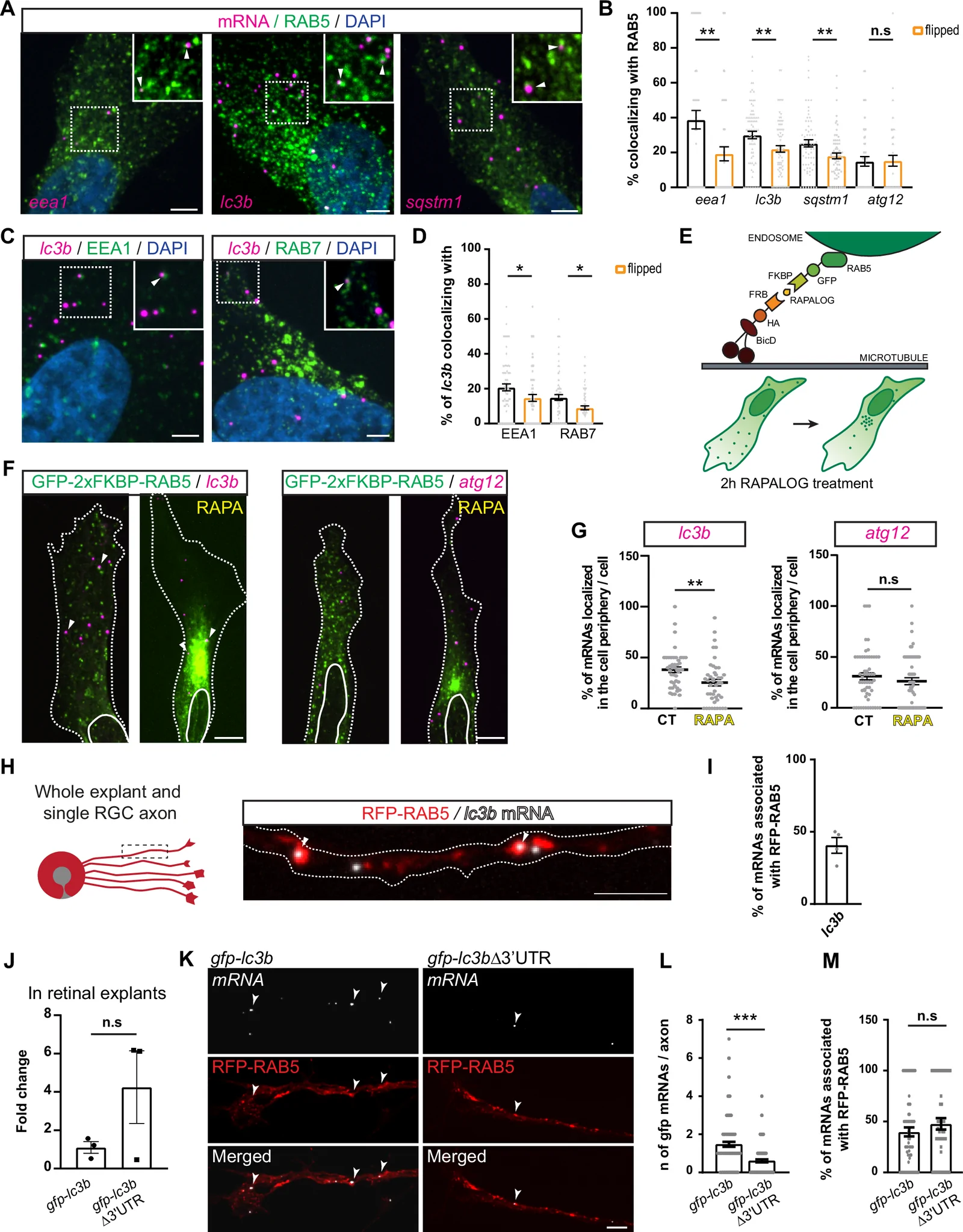
*lc3b* mRNA localizes to endosomes in neuronal-like cells and neuronal axons. (**A**) smFISH images of *eea1*, *lc3b*, and *sqstm1* mRNAs (in magenta) stained against RAB5 (in green) in SH-SY5Y cells. Arrows indicate mRNA signal colocalizing with RAB5 signal. (**B**) Colocalization analysis. Dots represent cells (*eea1:n* = 59 cells, *P* = 0.0087, 3 exp; *lc3b*: *n* = 75 cells, *P* = 0.0029, 3 exp; *sqstm1*: *n* = 68 cells, *P* = 0.0067, 3 exp; *atg12*: *n* = 48 cells, *P* = 0.8976, 3 exp). (**C**) smFISH images of *lc3b* mRNA (in magenta) stained against EEA1 or RAB7 (in green). Arrows indicate colocalizing mRNA signal. (**D**) Colocalization analysis. Dots represent cells (EEA1: *n* = 70 cells, *P* = 0.0211, 3 exp; RAB7: *n* = 82 cells, *P* = 0.0121, 3 exp). (**E**) Scheme of rapalog-based heterodimerization and predicted redistribution of RAB5-endosomes upon treatment. (**F**) smFISH images of *lc3b* and *atg12* mRNAs (in magenta) in SH-SY5Y cells expressing GFP-2xFKBP-RAB5 and HA-BICD-FRB treated with control (CT) or 100 nM rapalog (RAPA) for 2 h. Arrows indicate colocalizing mRNA signal. (**G**) Dots represent cells. Quantification of *lc3b* (CT: *n* = 45 cells, RAPA: *n* = 44 cells, *P* = 0.0010, 3 exp) and *atg12* (*n* = 57 cells, *P* = 0.2805, 3 exp) mRNAs subcellular distribution. (**H**) smFISH images of *lc3b* mRNAs (in white) in axons expressing RFP-RAB5. (**I**) Colocalization analysis. Dots represent mean per experiment. RAB5 colocalizing with *lc3b*: *n* = 62 fields of view, *n* ≥8 explants, 4 exp. (**J**) RT-qPCR analysis of *gfp* expression (*P* = 0.7000, 3 exp). (**K**) smFISH images showing *gfp-lc3b or gfp-lc3b-Δ3’UTR* mRNA signal (in white) in axons expressing RFP-RAB5 (in red). Arrows indicate mRNA signal colocalizing with RAB5 signal. (**L**) Quantification of *gfp* mRNA puncta in distal axons (*gfp-lc3b*: *n* = 145 axons, *gfp-lc3b-Δ3’UTR*: *n* = 146 axons, *P*  <  0.0001, *n* ≥6 explants, 3 exp). (**M**) Colocalization between mRNA puncta and RFP-RAB5 in axons (*gfp-lc3b*: *n* = 72 axons, *gfp-lc3b-Δ3’UTR*: *n* = 47 axons, *P* = 0.3076, *n* ≥8 explants, 4 exp). (**B**, **D**, **G**, **J**, **L**, **M**) Mann–Whitney test. Data is represented as mean ± SEM. Scale bars: (**A**, **C**, **H**, **K**) 3 µm, (**K**) 5 μm and (**F**) 10 µm. Source data are available online for this figure.

To validate our APEXseq results, we combined single molecule FISH (smFISH) with IF experiments in SH-SY5Y cells. EEA1 (Early Endosome Antigen 1) is a RAB5 effector that regulates EE tethering and fusion, and its encoding mRNA has previously been reported to localize to EEs (preprint: Popovic et al, 2020) and was identified in our APEX2-RAB5A condition. We therefore used it as a positive control and quantified its colocalization with RAB5-positive endosomes (Fig. 2A,B). We then examined the presence of mRNAs encoding well-known autophagy regulator. We focused on the transcript encoding LC3B (*lc3b*), a ubiquitin-like protein of the ATG8 family that regulates autophagic membrane dynamics and serves as a platform for recruitment of cargo receptors, and SQSTM1 (*sqstm1/p62*), a selective autophagy receptor that binds ubiquitinated cargo and LC3 to mediate cargo sequestration (Galluzzi et al, 2017; Siva Sankar and Dengjel, 2021). Owing to their central and validated functional roles, LC3B and SQSTM1 are widely used as canonical markers for monitoring autophagic activity, including in neurons (Stavoe and Holzbaur, 2019). Our smFISH results showed colocalization between these transcripts and RAB5 signal (Fig. 2A,B) and these associations were not random, as horizontally flipping one channel significantly reduced the colocalization (Fig. 2B). As a control, we also included the autophagy-related *atg12* mRNA, which was not detected in our APEX-seq experiment, and found no significant colocalization with RAB5 (Fig. 2B). The association between *lc3b* mRNA and EEs was further validated by using EEA1 as an additional endogenous marker for EEs (Fig. 2C,D). Moreover, these associations were not confined to EEs alone, as significant colocalization was also observed with the late endosome marker RAB7 (Fig. 2C,D).

Endosomes form dynamic contact sites with other organelles, and the biotinylation radius of APEX2 may capture transcripts from these adjacent structures. To further probe the spatial relationship between *lc3b* mRNA localization and RAB5-positive endosomes, we used an inducible heterodimerization system that allows rapid repositioning of specific organelles following treatment with a rapamycin analog (rapalog) (Fig. 2E) (Kapitein et al, 2010). Under control conditions, a subset of *lc3b* mRNAs were localized to the periphery of the cell where it can colocalize with RAB5 signal (Fig. 2F,G). Rapalog-induced repositioning of RAB5-positive endosomes toward the cell center caused a modest but significant shift in the spatial distribution of *lc3b* mRNAs, with fewer mRNAs localized peripherally and more transcripts accumulating in the perinuclear region (Fig. 2F,G). The effect is coherent with the proportion of transcripts associated with RAB5-positive vesicles and compatible either with *lc3b* mRNA co-redistributing with these organelles upon repositioning or with an increased perinuclear availability of endosomes providing more potential interaction sites. Importantly, this effect was specific, as rapalog treatment did not alter the spatial distribution of *atg12* mRNA (Fig. 2F,G). Together, these results indicate that redistribution of RAB5-positive endosomes selectively influences the localization of *lc3b* mRNA, consistent with binding of the transcript to the organelle.

**Figure EV2:**
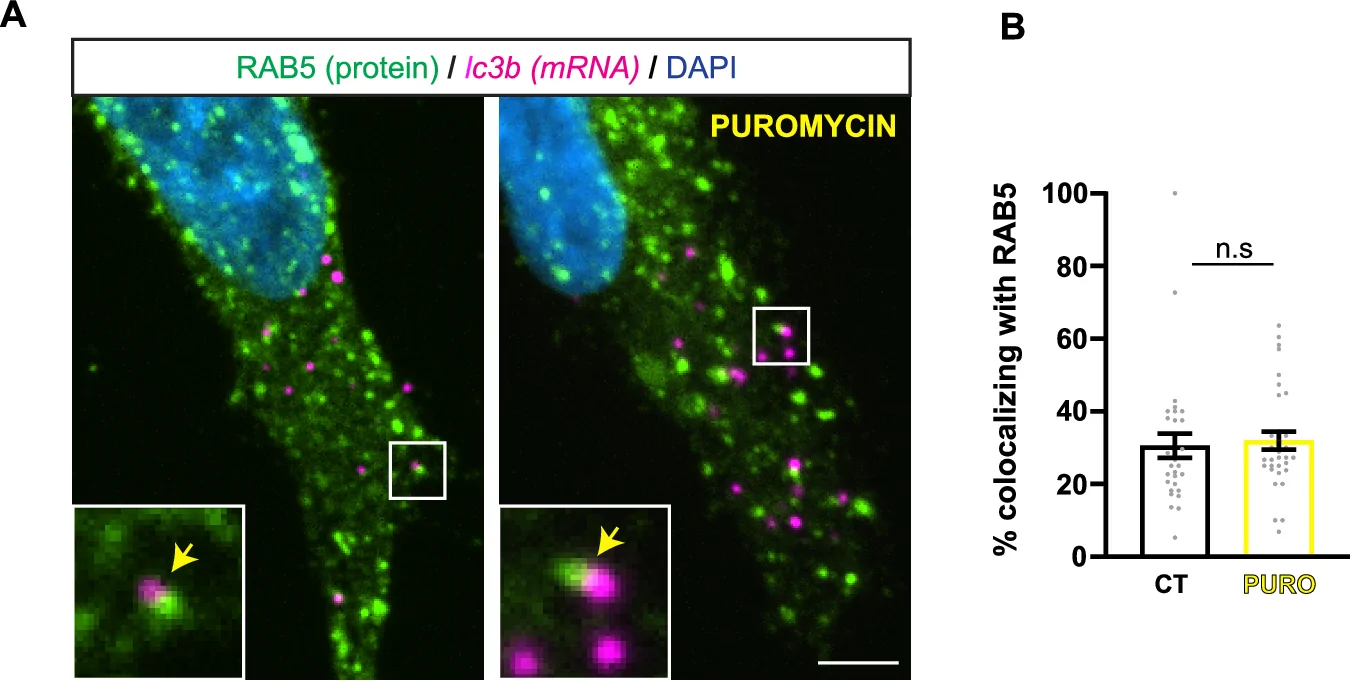
*lc3b* mRNA association with RAB5-positive endosomes is puromycin insensitive. (**A**) smFISH images of *lc3b* mRNA (in magenta) in SH-SY5Y cells treated with vehicle or 100 μM puromycin and stained against RAB5 (in green). Arrows indicate *lc3b* mRNA colocalizing with RAB5 signal. Scale bar: 3 μm. (**B**) Colocalization analysis. Dots represent cells (vehicle *n* = 30 cells, puromycin *n* = 33 cells, *P* = 0.2804, 3 exp). Mann–Whitney test. Mean ± SEM.

Previous studies have shown that the association of some mRNAs with EEs can depend on active translation (preprint: Popovic et al, 2020; Chouaib et al, 2020). To test this for *lc3b* mRNA, SH-SY5Y cells were treated with puromycin prior to smFISH analysis. Puromycin induces the dissociation of nascent peptide chains from ribosomes and associated mRNAs, thereby disrupting translation-dependent mRNA localization. Despite inhibition of protein synthesis, no significant change was observed in the colocalization between *lc3b* mRNA and RAB5-positive endosomes compared to control conditions (Fig. EV2). These findings indicate that the association of *lc3b* mRNA with endosomes does not require ongoing translation.

**Figure EV3:**
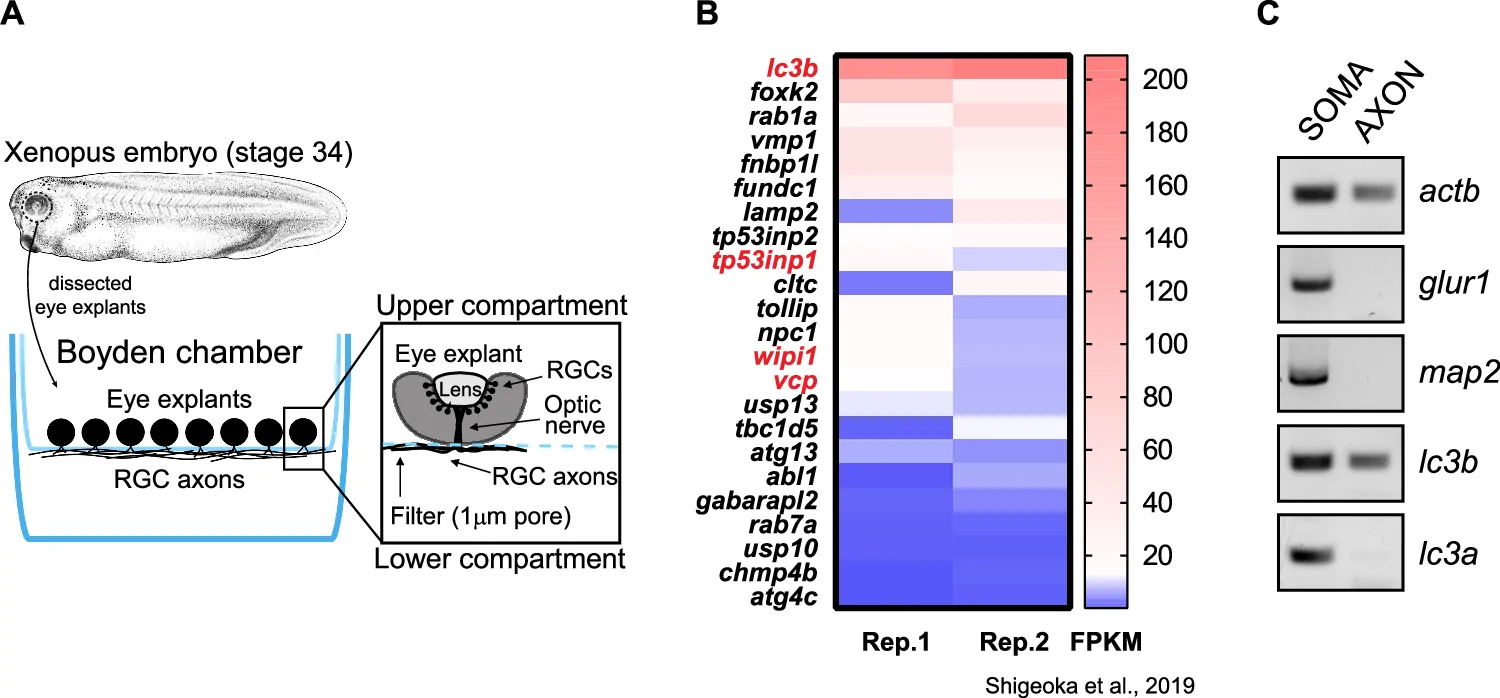
Autophagy-related mRNAs are present in *Xenopus* RGC axons. (**A**) Schematic representation of compartmentalized chambers (Boyden chambers) used to isolate somatodendritic and axonal compartments of *Xenopus* RGCs. (**B**) Heatmap showing the ranking of abundance (average FPKM) of autophagy-related mRNAs in *Xenopus* RGC axons (Shigeoka et al, 2019). Red indicates mRNA identified by APEXseq in Fig. 1. (**C**) RT-PCR analysis of cDNA synthesized from somatodendritic and axonal RNAs (*n* = 3 exp.). *actb*, *glur1* and *map2* mRNAs (on the left) show purity of axonal RNAs.

To extend our findings and investigate mRNA association with endosomes in neuronal compartments, we selected retinal ganglion cell (RGC) axons from *Xenopus laevis* as a model system. Analysis of previously generated axonal RNA-seq data obtained using compartmentalized chambers (Boyden chambers, Fig. EV3A), revealed the presence of several autophagy-related mRNAs, including some identified in our APEXseq (Fig. EV3B) (Shigeoka et al, 2019). *lc3b* mRNA was the most highly expressed in axons, and its presence was confirmed by RT-PCR (Fig. EV3C). In contrast, *lc3a* mRNA, encoding another member of the ATG8/LC3 family with overlapping but not identical functions to LC3B in autophagy, was not detected in axons (Fig. EV3B,C), supporting the specificity of *lc3b* mRNA axonal localization. We then investigated its association with RAB5-positive endosomes in axons. However, when coupled with IF, the sensitivity of our smFISH method decreases and we did not detect a reliable positive signal for *lc3b* transcript in axons. We therefore performed smFISH alone on RGC axons expressing RFP-tagged RAB5 (Fig. 2H). We obtained a positive signal for *lc3b* mRNAs along the axon shaft, and colocalization analyses revealed that approximately 40,6% of *lc3b* transcripts were localized to RAB5-labelled vesicles (Fig. 2I), confirming endosomal association in axons.

We next wanted to explore the regulatory mechanisms governing the axonal localization of *lc3b* mRNA and its association with EEs. mRNA distribution is often regulated by cis-regulatory elements in the 3’ untranslated region (3’UTR) (Mayr, 2019). To test if the subcellular localization of *lc3b* mRNA is regulated by its 3’UTR, we expressed equimolar levels of *gfp-lc3b* mRNA with or without its 3’UTR. First, we verified mRNA stability (Heraud-Farlow et al, 2013; Tushev et al, 2018), and RT-qPCR analysis showed that the overall levels of the 3’UTR-depleted (Δ3’UTR) mRNA were similar or higher in retinal explants (Fig. 2J). Then, we investigated axonal localization of the two mRNA variants by smFISH. Despite the higher expression in the retina, *gfp-lc3b-Δ3’UTR* mRNA was significantly less localized to axon terminals compared to the wild-type form (Fig. 2K,L). However, we observed no significant difference in the percentage of association with RFP-RAB5 between the two transcripts along the axon shaft (Fig. 2K,M), supporting that the coding region is sufficient for mRNA localization to endosomes. Our findings therefore suggest that the axonal distribution and endosome targeting of *lc3b* mRNA are two different processes, potentially governed by separate molecular mechanisms.

### *lc3b* mRNA is translated at RAB5-positive endosomes in neuronal axons

**Figure EV4:**
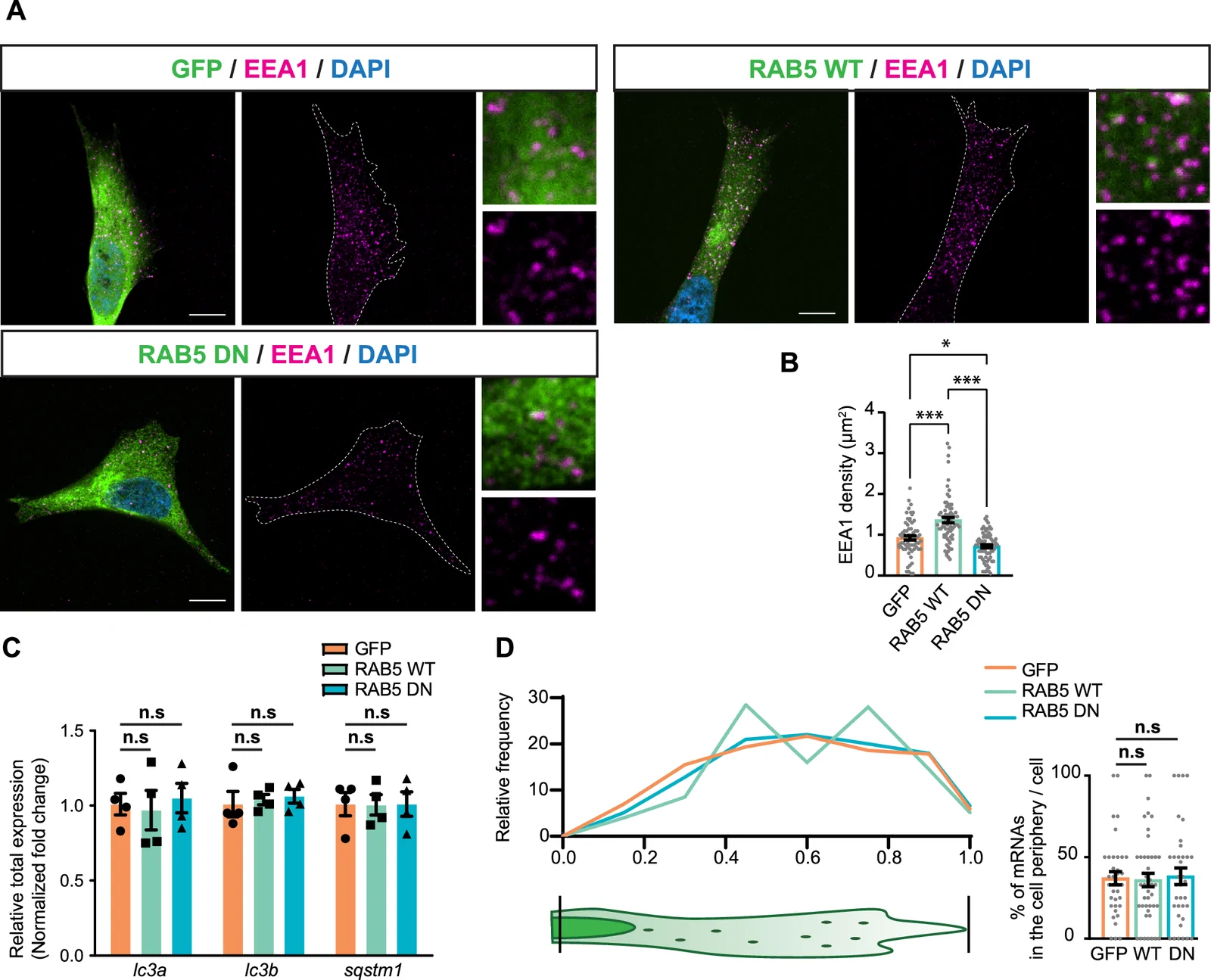
Alteration of RAB5 activity does not impair *lc3b* mRNA expression or its distribution in SH-SY5Y cells. (**A**) IF images of EEA1 signal (in magenta) in SH-SY5Y cells expressing the indicated constructs (in green). (**B**) Quantification of EEA1 signal density. Dots represent cells (GFP: *n* = 64 cells, RAB5WT: *n* = 74 cells; RAB5DN: 76 cells, *n* = 3 exp). (**C**) RT-qPCR analysis in SH-SY5Y cells expressing the indicated constructs (*n* = 4 exp). (**D**) *lc3b* mRNA distribution in SH-SY5Y cells expressing the indicated constructs. Quantification of the relative frequency distribution of mRNA distances from the nucleus relative to the maximum cell extension. Dots represent cells (GFP: *n* = 36 cells, RAB5WT: *n* = 43 cells; RAB5DN: 36 cells, *n* = 3 exp). Mean ± SEM. (**B**–**D**) Kruskal–Wallis test. Scale bar: (**A**) 10 μm.

To next investigate the functional significance of *lc3b* mRNA localization to RAB5-positive endosomes, we asked whether perturbing RAB5 activity, and thereby altering the endosomal pathway, could affect *lc3b* mRNA expression and/or distribution. To test this, we first overexpressed wild-type (RAB5WT) or dominant-negative (RAB5DN) RAB5 in SH-SY5Y cells. Quantification of EEA1 IF signal density revealed the expected decrease and increase in EEA1-positive endosomes in RAB5DN and RAB5WT cells, respectively, compared to GFP-expressing controls (Fig. EV4A,B). However, transcript levels of *lc3a*, *lc3b*, and *sqstm1* were unchanged across conditions (Fig. EV4C). Similarly, *lc3b* mRNA distribution was unaffected by RAB5WT or RAB5DN expression compared to GFP controls (Fig. EV4D), indicating that proper RAB5-dependent EE trafficking is not required for the transcription, stability and spatial distribution of these transcripts. Together with the effects observed upon forced EE repositioning (Fig. 2F,G), these results support a model in which EEs act as subcellular docking sites for *lc3b* mRNA rather than as active trafficking units.

**Figure 3:**
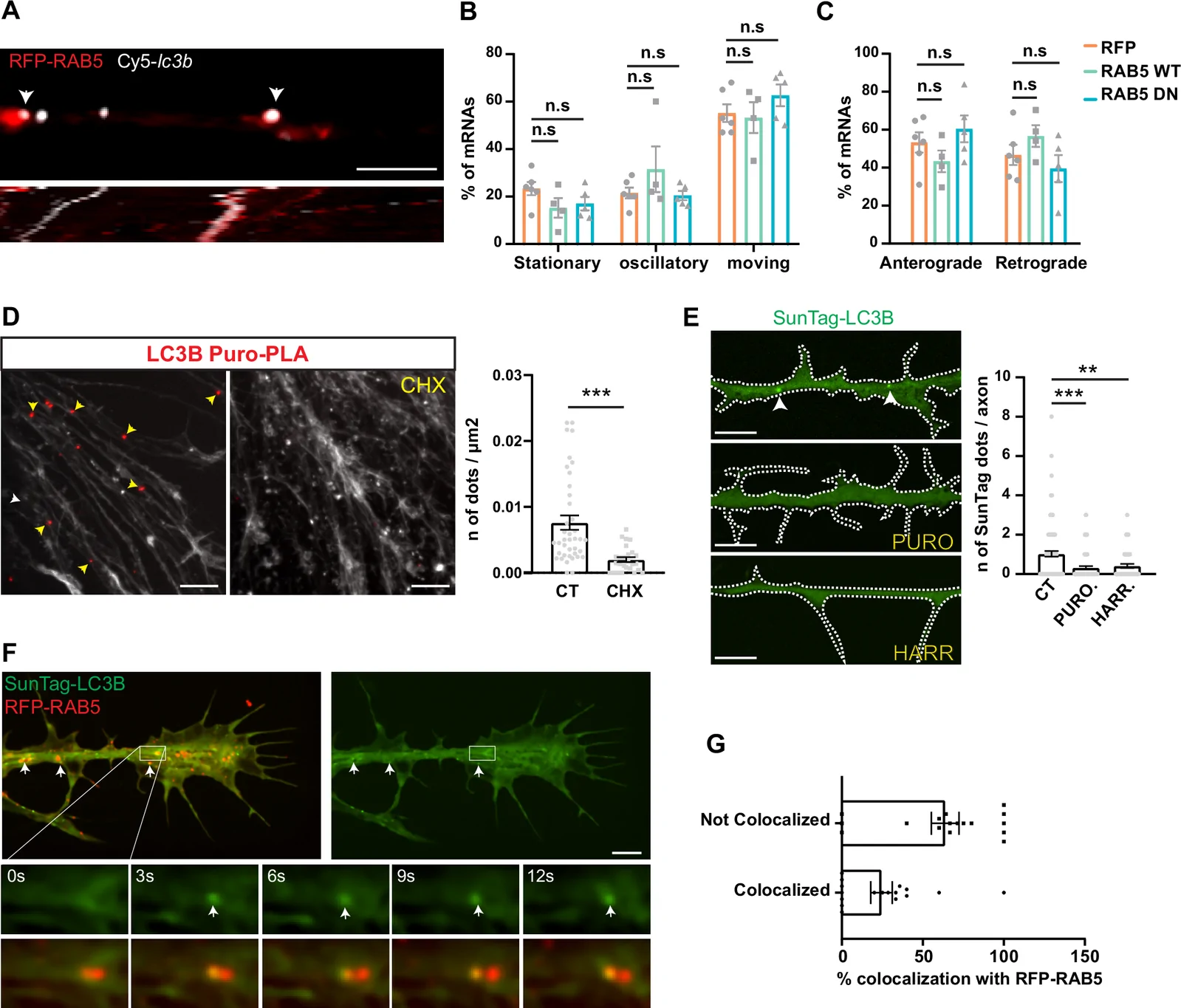
LC3B is synthesized on RAB5-positive endosomes in axons. (**A**) Image and kymograph (2 min) of time-lapse sequence showing Cy5*-lc3b* (in white) and RFP-RAB5 (in red) expression in axons. Arrows indicate mRNA puncta associated with RAB5. (**B**, **C**) Quantitative analysis of the motion-types or directionality of Cy5-*lc3b* mRNAs in axons expressing the indicated constructs (RFP: *n* = 79 axons; RAB5WT: *n* = 53 axons; RAB5DN: *n* = 71 axons). Dots represent mean/experiments (4 exp.). (**D**) Images showing LC3B Puro-PLA signal in axons and related quantification in the indicated conditions (CT (DMSO), *n* = 39 fields of view; CHX, *n* = 26 fields of view; *P*  <  0.0001, *n* ≥6 explants, 3 exp). Arrows indicate Puro-PLA dots (red) in axons (white). (**E**) Images showing SunTag-LC3B foci (indicated by arrows) in axons. Quantification of SunTag-LC3B foci in axons in Control (CT, *n* = 101 axons) or treated with Puromycin (PURO, *n* = 88 axons, *P*  <  0.0001) or Harringtonine (HARR., *n* = 48 axons, *P*  = 0.0092), *n* ≥6 explants, 3 exp. (**F**) Image and time-lapse sequence showing SunTag-LC3B foci (indicated by arrow) in axon expressing RFP-RAB5. Time is indicated in seconds (s). (**G**) Colocalization between RFP-RAB5 and SunTag-LC3B foci in axons. *n* = 17 axons, *n* ≥4 explants, 2 exp. Mean ± SEM. (**D**) Mann–Whitney test. (**B**, **C**, **E**) Kruskal–Wallis test. Scale bars: (**A**) 2.5 μm, (**D**) 10 μm, (**E**) 4 μm, and (**F**) 5 μm. Source data are available online for this figure.

We then turned to RGC neurons and conducted live imaging in axons expressing a fixed concentration of Cy5-labeled *lc3b* mRNAs. Co-expression with RFP-RAB5 revealed visible association events between RAB5 and *lc3b* mRNA signals, typically characterized by oscillatory movements and short-range displacements (Fig. 3A). Automated tracking analysis showed no significant changes in the global dynamics of Cy5-*lc3b* mRNA puncta in axons with altered RAB5 activity (Fig. 3B,C). Overall, our results are consistent with previous observations of RNA granule trafficking in the same system (Cioni et al, 2019) and do not support a significant role for EE trafficking in the axonal transport of *lc3b* mRNA.

Given the lack of strong evidence for a role in trafficking, we decided to explore alternative functional implications of *lc3b* mRNA association with EEs. Since ribosomes have been reported to be present on endosomes (Baumann et al, 2014; Higuchi et al, 2014), including in RGC axons (Cioni et al, 2019), we considered the possibility that RAB5-positive endosomes might serve as sites for *lc3b* mRNA translation. We first tested the axonal synthesis of the endogenous protein using puromycilation coupled with a proximity ligation assay (Puro-PLA) (tom Dieck et al, 2015). A positive Puro-PLA signal for LC3B was observed along the axon shaft (Fig. 3D). Moreover, the number of visible dots was significantly reduced by the protein synthesis inhibitor cycloheximide (CHX), confirming axonal protein synthesis. To determine whether *lc3b* mRNA is actively translated at RAB5-positive endosomes, we employed live single-molecule nascent polypeptide imaging using the SunTag fluorescent tagging system. In this assay, the repeated GCN4 epitope sequences engineered into LC3B coding region enables newly synthesized polypeptides to be rapidly decorated by GFP-fused scFv (scFv-GFP) upon emergence from the ribosome, resulting in localized fluorescent foci at sites of active translation (Wang et al, 2016; Wu et al, 2016; Yan et al, 2016). Co-expression of *SunTag-lc3b* with scFv-GFP led to the appearance of few scattered fluorescent foci along the axon shaft (Fig. 3E). As expected, the signal was inhibited when protein synthesis was blocked through exposure to a high concentration of puromycin (PURO) or harringtonine (HARR), confirming the presence of translating mRNAs (Fig. 3E). Further expression of RFP-RAB5 revealed the appearance of SunTag-LC3B signal at RAB5-positive endosomes (Fig. 3F), thereby supporting endosomal-associated protein synthesis. Interestingly, quantification revealed that 24,5% of the visible foci colocalized with the RAB5 signal (Fig. 3G), suggesting that RAB5-positive endosomes are not the exclusive sites of translation for this mRNA in axons. Taken together, these results indicate that *lc3b* mRNA does not require proper RAB5 activity for axonal localization, but rather, could dock to endosomes where translation of the mRNA can occur.

### RAB5-positive endosomes are targeted by autophagy in neuronal axons

Why is *lc3b* mRNA translated at RAB5-positive endosomes in axons? This question arises as LC3B protein is predominantly a marker for autophagosomes (APs), which are double-membraned vesicles primarily known for engulfing cargo, including large organelles, for degradation. Recently, LC3B has been shown to be involved in endosomal homeostasis in various cell lines. The molecular machinery involved in LC3B regulation can be recruited and activated at endosomes (Millarte et al, 2022), and impairing LC3B function leads to the accumulation of EEs (Fraser et al, 2019). Therefore, we hypothesized that, as it has previously been observed for other organelles such as mitochondria (Ashrafi et al, 2014) and the ER (Kuijpers et al, 2021), endosomes in axons could be locally regulated by LC3B-mediated autophagy.

**Figure 4:**
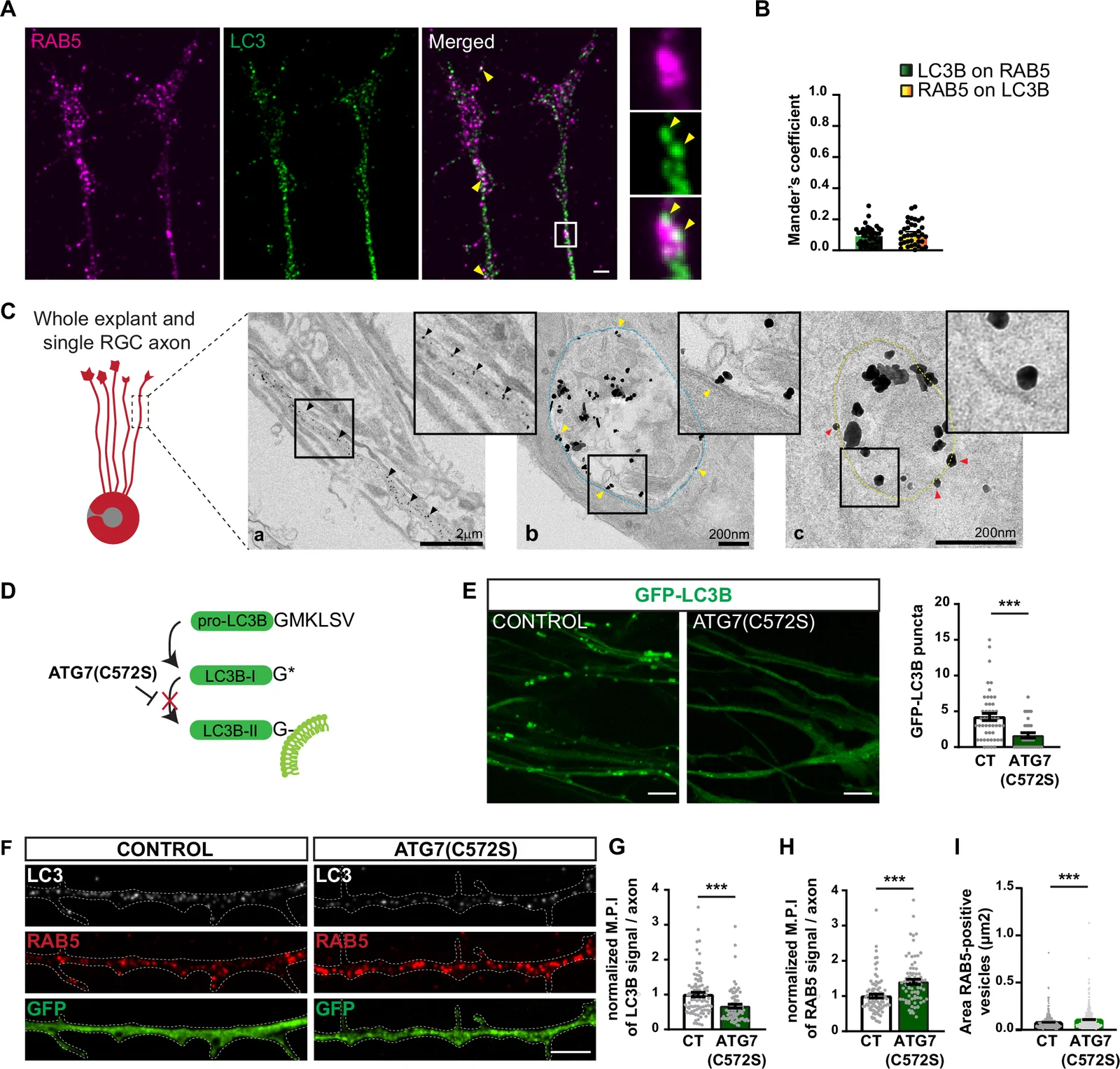
LC3B regulates RAB5-positive endosomes in axons. (**A**) IF image of RAB5 (in magenta) and LC3B (in green) stainings in RGC axons. Arrows indicate colocalization. (**B**) Mander’s colocalization coefficient. Dots represent axons. (*n* = 37 axons, n≥6 explants, 3 exp.). (**C**) Images of TEM coupled with immunogold labeling of GFP-LC3B in axons. Arrowheads indicate positive signals along (**a**) microtubules, (**b**) in and on double-membrane (blue dashed line), and (c) in and on single-membrane vesicles (yellow dashed line). (**D**) Scheme illustrating the dominant negative effect of ATG7(C572S). (**E**) Images and quantification of GFP-LC3B puncta. Dots represent axons (*n* = 48 in CT, *n* = 31 in ATG7(C572S), *P*  <  0.0001, *n* ≥4 explants, 2 exp). (**F**) Images of RAB5 IF signal in axons co-expressing GFP alone or GFP and ATG7(C572S). (**G**) Quantification of LC3B signal (mean pixel intensity). Dots represent axons. (*n* = 90 in CT, *n* = 78 in ATG7(C572S), *n* ≥6 explants, 3 exp), *P*  <  0.0001, *n* ≥6 explants, 3 exp. (**H**) Quantification of RAB5 signal (mean pixel intensity). Dots represent axons (*n* = 90 in CT, *n* = 78 in ATG7(C572S), *P*  <  0.0001, *n* ≥6 explants, 3 exp). (**I**) Area of RAB5 positive signal in axons. Dots represent vesicles. (*n* = 244 in CT, *n* = 273 in ATG7(C572S), *P*  <  0.0001, *n* ≥6 explants, 3 exp). Mean ± SEM. (**E**, **G**, **H**, **I**) Mann–Whitney test. Scale bars: (**A**) 2 μm, (Ca) 2 μm, (Cb and Cc) 200 nm, (**E**) 3 μm, and (**F**) 5 μm. Source data are available online for this figure.

To explore this idea, we first investigated LC3B localization. The protein can be lipidated and incorporated into membranes, forming distinct punctate structures. IF experiments revealed sparse but clear colocalization between RAB5 and LC3B signals in axons (Fig. 4A,B). These results may reflect the presence of the endosomal marker within APs or direct LC3B insertion into endosomal membranes. Indeed, endosomes can be regulated by both canonical and non-canonical autophagy. In canonical autophagy, they are sequestered into APs (Fraser et al, 2019), whereas in non-canonical pathways, LC3B is directly conjugated to single membranes (Heckmann et al, 2019). We therefore turned to transmission electron microscopy (TEM) to assess the morphology of the organelles labelled by GFP-LC3B in axons (Fig. 4C). Our analyses first revealed the presence of signals linearly distributed along axons on structures resembling microtubules (Fig. 4Ca). As expected, we also detected LC3B clusters on double-membrane structures (Fig. 4Cb), likely representing APs. Smaller vesicles with sizes that can range from 50 to 250 nm in diameter were clearly visible inside APs, consistent with, and suggestive of, the presence of endosomal structures. Intriguingly, we also occasionally detected LC3B signal on single-membrane vesicles (Fig. 4Cc), which appeared to correspond morphologically to multivesicular bodies and endosomes along the axon shaft. While we could not directly assess if these vesicles were labelled by locally synthesized LC3B, these results indicated that the protein was present on vesicles beyond APs in distal axons and are consistent with the possibility that both canonical and non-canonical autophagy pathways may be occurring.

**Figure EV5:**
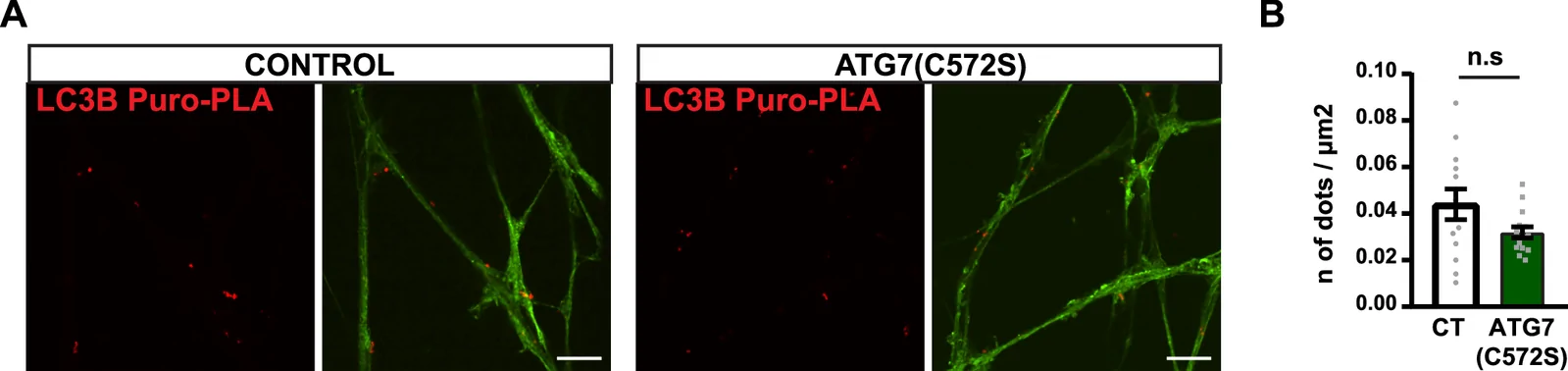
Expression of the ATG7(C572S) mutant does not affect LC3B synthesis in axons. (**A**) Images showing LC3B Puro-PLA signal in axons. Scale bar: 10 μm. (**B**) Quantification in the indicated conditions (CT, *n* = 13 fields of view; ATG7(C572S), *n* = 15 fields of view; *n* ≥6 explants, 3 exp). Mean ± SEM. (**B**) Unpaired *t* test.

If LC3B participates in endosomal turnover in axons, blocking this process should lead to their accumulation. LC3B function in autophagy depends on its lipidation and insertion into membranes, a process enabled by several ATG proteins, including ATG7. ATG7 is required for recruiting the ATG12-ATG5-ATG16L1 complex responsible for the conjugation of LC3B to phospholipids in membranes. To block LC3B function in RGC axons, we expressed a mutated form of ATG7 (C572S), which has been shown to block ATG12-ATG5 conjugation and thereby impair LC3B lipidation and membrane association (Nitta et al, 2019; Tanida et al, 2001) (Fig. 4D). Co-expression with GFP-LC3B revealed a significant reduction in LC3B-positive puncta in ATG7(C572S)-expressing axons (Fig. 4E), confirming the dominant negative effect of the mutation in our system. These results were further confirmed by IF analysis, which showed a decrease in LC3B signal in axons expressing ATG7(C572S) compared to control (Fig. 4F,G). Notably, PuroPLA analysis showed no significant change in LC3B axonal synthesis rates in axons expressing ATG7(C572S) (Fig. EV5), indicating a restricted effect of the mutant on LC3B lipidation. To then test whether blocking LC3B lipidation affects EEs in axons, we quantified RAB5 IF signal and found a significant increase in signal intensity and size in axons expressing ATG7(C572S) compared to control (Fig. 4F,H,I), supporting an accumulation of enlarged endosomes in these axons. Taken together, our results support a low but constitutive targeting of RAB5-positive endosomes by LC3B-mediated autophagy in axons and show that impairing LC3B function locally alters endosomal homeostasis.

### Defective RAB5-positive endosomes harboring *lc3b* mRNA can directly transition to LC3B-positive structures along the axon shaft

Enlarged endosomes have been associated with impaired function and can be experimentally induced in neurons by overexpressing RAB5, a condition shown to trigger degenerative features associated with Alzheimer’s disease (AD) (Pensalfini et al, 2020). Therefore, we hypothesized that increasing RAB5 activity would lead to dysfunctional endosomes in axons, which would then be targeted by LC3B for clearance. This would further support the role of this process in endosomal quality control in axons.

**Figure EV6:**
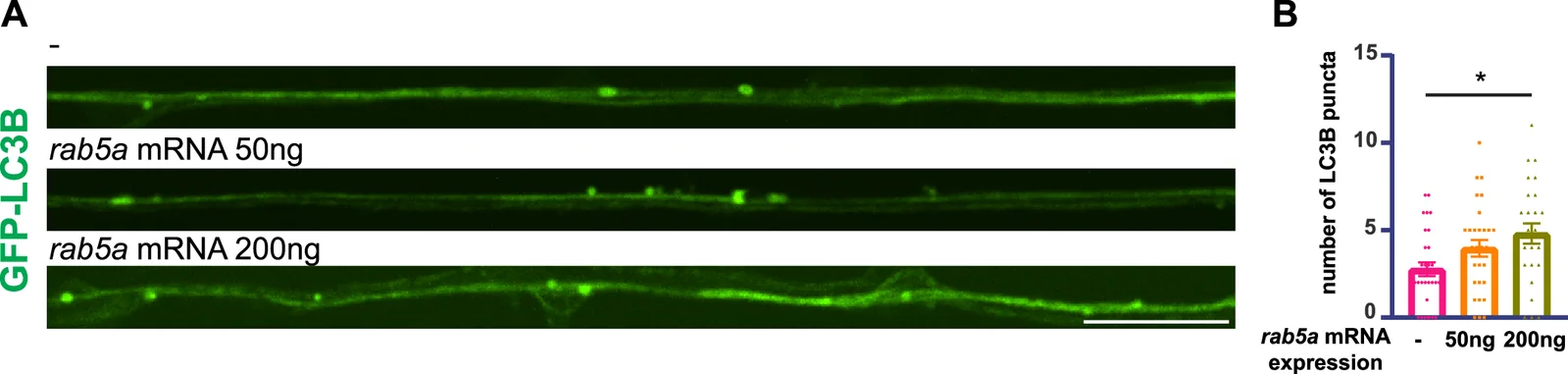
RAB5 overactivation drives an increase in GFP-LC3B puncta in axons. (**A**) Images showing GFP-LC3B signal in axons expressing increased concentration of *rab5a* mRNA. (**B**) Quantification of GFP-LC3B puncta. Dots represent axons (CT: *n* = 30; RAB5 50 ng: *n* = 29; RAB5 200 ng: *n* = 26, *P = 0.0107, *n* ≥4 explants, 2 exp). Kruskal–Wallis test. Mean ± SEM. Scale bar: (**A**) 10 μm.

**Figure 5:**
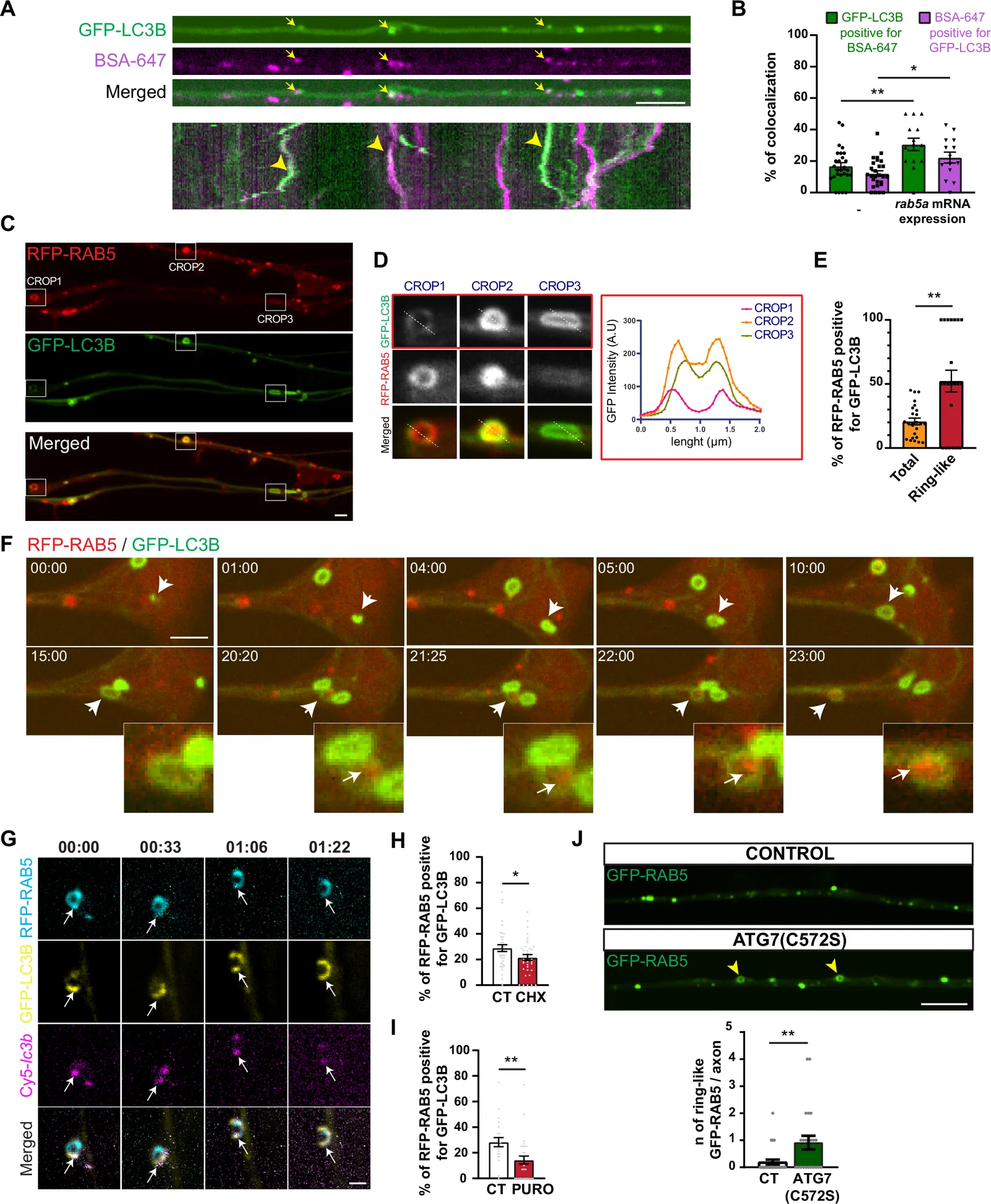
Dysfunctional endosomes generated by RAB5 overexpression become LC3B-positive, and LC3B function regulates their clearance in axons. (**A**) Image and kymograph (2 min) showing BSA-647 signal (in magenta) in axons expressing GFP-LC3B (in green). Arrows indicate colocalizing signals. (**B**) Colocalization analysis. Dots represent axons (CT: *n* = 27; RAB5 100 ng: *n* = 14, **P = 0.0021, *P = 0.0127, *n* ≥6 explants, 3 exp). (**C**) Representative image showing RFP-RAB5 and GFP-LC3B signals in axons. (**D**) Expanded views and intensity plot of the GFP-LC3B signal along the indicated line. (**E**) Colocalization analysis. Dots represent axons (*n* = 25, *P* = 0.0091, *n* ≥6 explants, 3 exp). (**F**) Time-lapse sequence showing an axon expressing RFP-RAB5 and GFP-LC3B. Arrows indicate nascent AP in the growth cone, in which RAB5 signal accumulates. (**G**) Time-lapse sequence of RFP-RAB5 (in blue), GFP-LC3B (in yellow) and Cy5-*gfp-lc3b* mRNA (in magenta) signals. Arrows indicate colocalization between signals. (**H**) Colocalization analysis. Dots represent axons. (CT: *n* = 38, CHX: *n* = 37, *P* = 0.0485, *n* ≥4 explants, 2 exp). (**I**) Colocalization analysis. Dots represent axons. (CT: *n* = 22, PURO: *n* = 29, *P* = 0.0011, *n* ≥6 explants, 3 exp). (**J**) Images and quantification of GFP-RAB5 signal. Arrows indicate ring-like endosomes. Dots represent axons (CT: *n* = 31, ATG7(C572S): *n* = 23, *P* = 0.0038, *n* ≥6 explants, 3 exp). Mean ± SEM. (**B**, **E**, **H**, **I**, **J**) Mann–Whitney test. Scale bars = (**A**, **J**) 5 μm, (**G**) 1 μm, and (**C**, **F**) 2 μm. Time is indicated as min:sec. Source data are available online for this figure.

We first globally assessed the effect of RAB5 overexpression on endosomal autophagy. Consistent with induction of autophagy, increased RAB5 expression led to a progressive increase in GFP-LC3B puncta in axons (Fig. EV6). To then examine the impact on endosomal autophagy, we decided to apply BSA-647 to axons, a fluorescent tracer that is internalized through endocytosis and accumulates within endocytic vesicles. This approach enabled us to visualize and track the endocytic compartment in axons expressing GFP-LC3B. Most of the endocytic vesicles colocalizing with GFP-LC3B exhibited either static or retrograde movement along the axon, consistent with their engagement in the retrograde autophagic degradative pathway previously described in axons (Maday et al, 2012) (Fig. 5A). In line with previous findings (Kulkarni et al, 2022), we found that 16.8% of GFP-LC3B–positive vesicles contained BSA-647, and 12.1% of BSA-647–labeled vesicles also had GFP-LC3B (Fig. 5A,B). This supports that a small fraction of endocytic vesicles is continuously regulated by autophagy in axons. When we overexpressed RAB5, both numbers increased significantly, 22.3% of BSA-647 vesicles colocalized with GFP-LC3B, and 30.6% of GFP-LC3B vesicles contained BSA-647 (Fig. 5B). Together, these results indicate that increased RAB5 expression, and therefore activity, enhances axonal autophagy and promotes increased autophagic targeting of endocytic vesicles.

**Figure EV7:**
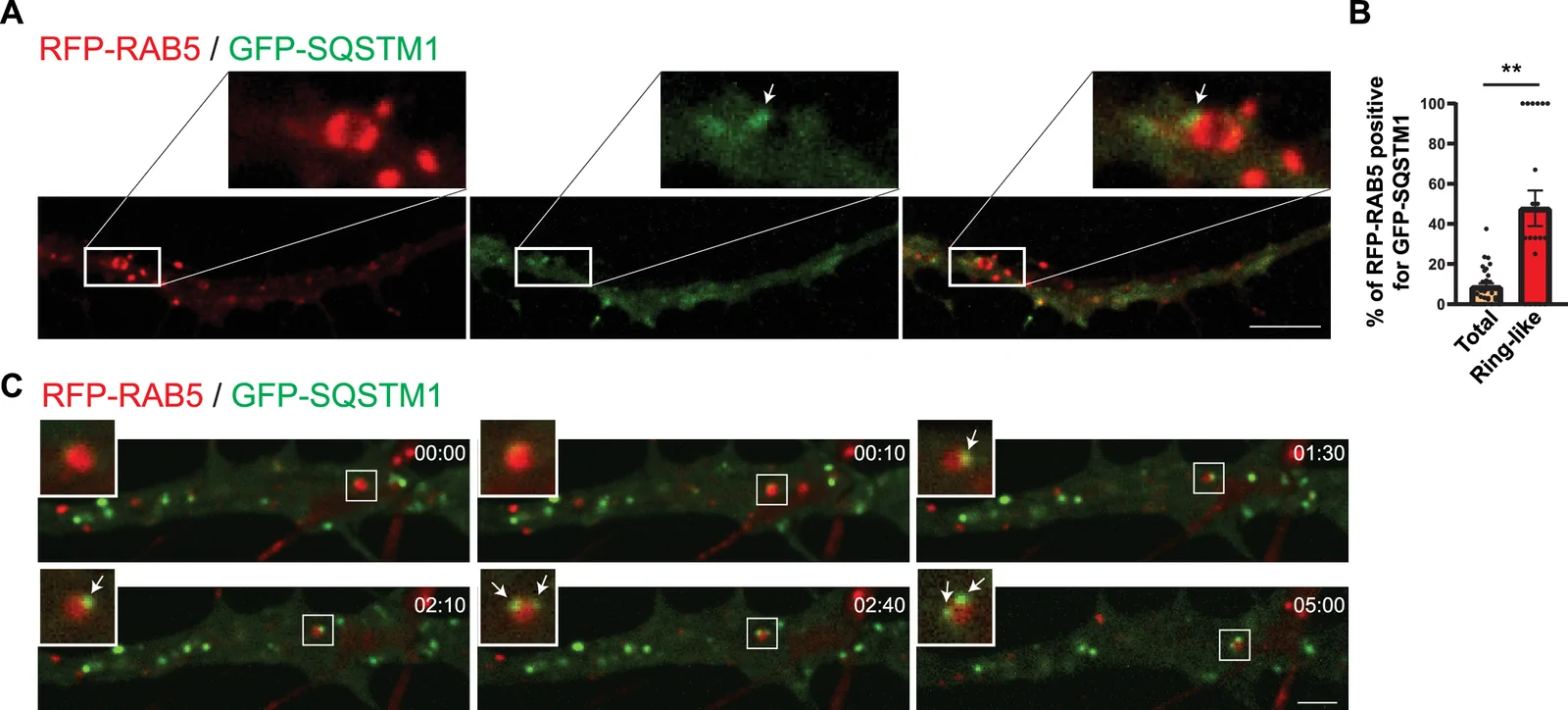
RAB5 overexpression induces enlarged endosomes positive for SQSTM1. (**A**) Image showing GFP-SQSTM1 signal colocalizing with ring-like RFP-RAB5 positive endosome in axons. Arrow indicates GFP-SQSTM1 signal on RFP-RAB5-endosome. (**B**) Colocalization between RFP-RAB5 and GFP-SQSTM1. Dots represent axons (*n* = 31, *n* ≥6 explants, *P* = 0.0011, 3 exp). Mann–Whitney test. Mean ± SEM. (**C**) Time-lapse sequence on axons expressing RFP-RAB5 and GFP-SQSTM1. Arrows indicate a RFP-RAB5-endosome becoming GFP-SQSTM1 positive. Time is indicated as min:sec. Scale bar: (**A**) 5 μm, and (**C**) 2 μm.

We next analyzed endosomal morphology in axons overexpressing RAB5. As previously reported, we observed a range of endosome sizes, including a distinct subset (6.4%) of enlarged, “ring-like” vesicles (Fig. 5C,D). In these axons, ~20% of the RAB5- vesicles were positive for GFP-LC3B, while 52% of these enlarged RAB5-positive endosomes displayed GFP-LC3B on their membrane (Fig. 5E), indicating a preferential targeting by LC3B. Interestingly, a detailed examination also revealed that the intensity of the GFP signal on these “ring-like” RAB5-positive membranes (Fig. 5D, CROP1) was approximately half when compared to GFP-LC3B-positive vesicles with the RAB5 signal inside their lumen (CROP2) or negatives for RAB5 (CROP3). Based on our TEM results, we hypothesized that this reflects the insertion of LC3B in single membranes compared to the signal resulting from its insertion into double-membraned APs. These enlarged endosomes were also positive for the autophagy receptor SQSTM1 (Fig. EV7), consistent with its role in linking ubiquitinated organelles to the autophagy machinery and supporting the idea that they represent dysfunctional endosomes.

To gain further mechanistic insights, we next analyzed time-lapse imaging sequences. We first observed that small RAB5-positive endosomes were gradually incorporated into nascent autophagosomes (APs) in distal axonal regions, ultimately accumulating within the AP lumen (Fig. 5F; Movie EV1). These results are consistent with canonical autophagy and likely reflect most of the colocalization between LC3B and endocytic markers in axons. We then restricted our attention to enlarged RAB5-positive endosomes to determine whether they could acquire LC3B along the axon shaft. Given that *lc3b* mRNA can be locally translated on RAB5-positive endosomes, we expressed a Cy5–*gfp–lc3b* transcript to simultaneously visualize mRNA localization and protein enrichment in real time. Strikingly, we were able to observe events of GFP-LC3B accumulating and expanding precisely at the organelle membrane where the mRNA signal was detected (Fig. 5G). These findings suggested that, in addition of being engulfed by nascent APs, dysfunctional RAB5-positive endosomes can acquire LC3B through locally synthesized protein. To test whether the colocalization between LC3B and RAB5 could depend on translation, we treated axons with the protein synthesis inhibitors cycloheximide and puromycin, both of which reduced the number of LC3B-positive, RAB5-positive endosomes (Fig. 5H,I). Finally, to assess the functional role of LC3B on these enlarged endosomes, we disrupted its lipidation by expressing the ATG7(C572S) mutant. This manipulation led to an accumulation of ring-like endosomes in axons overexpressing RAB5 (Fig. 5J), supporting a role for LC3B in the clearance of dysfunctional endosomal structures.

Together, these findings support the idea that autophagy contributes to the regulation of axonal endosomes and suggested that mRNA localization may participate in this process by targeting dysfunctional endosomes along the axon shaft.

### Chloroquine induces endosomal stress and LC3B expression on axonal Rab5-endosomes

**Figure 6:**
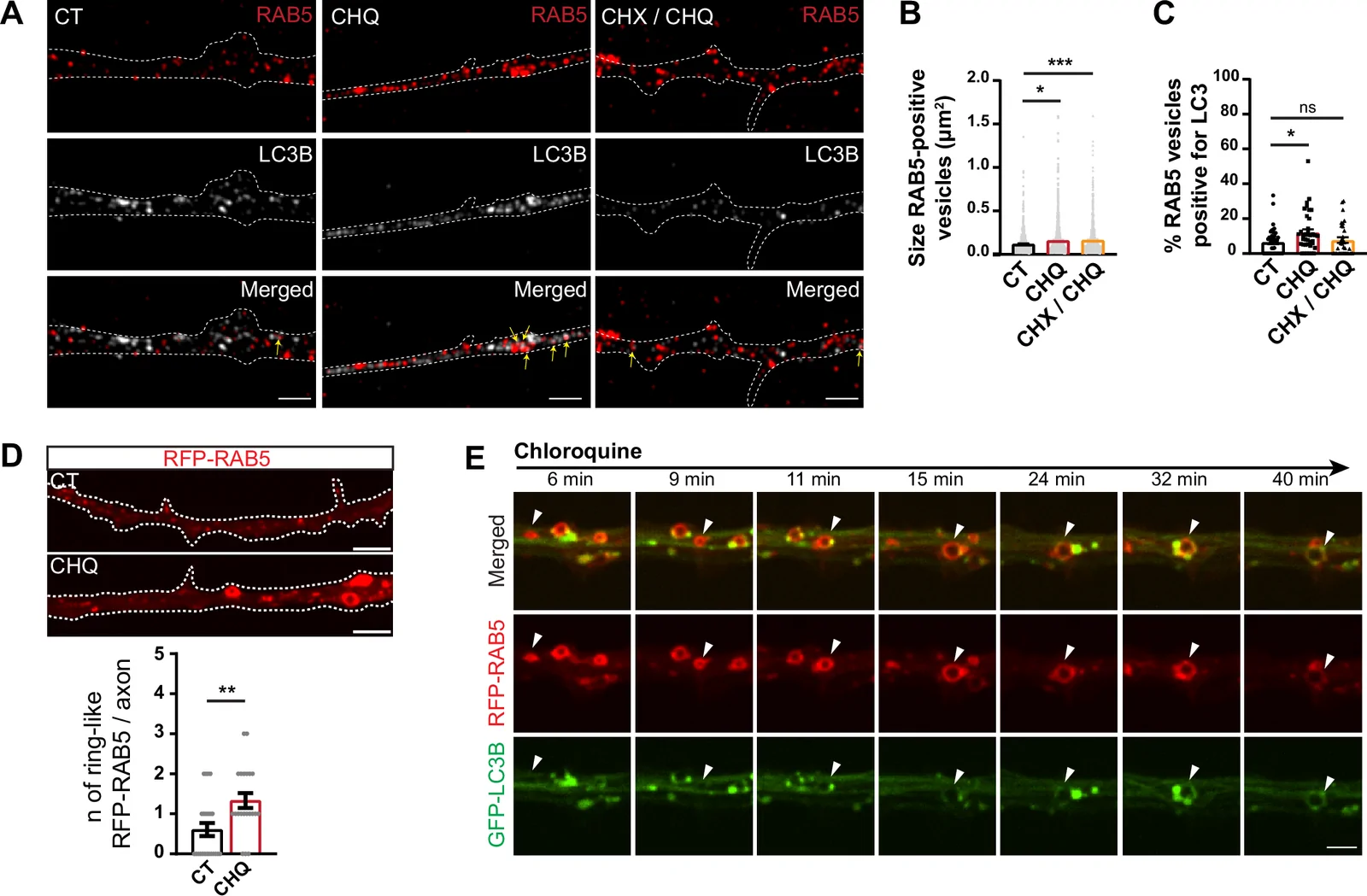
Chloroquine induces endosomal swelling and protein synthesis-dependent LC3B recruitment in axons. (**A**) IF images of RAB5 (in red) and LC3B (in white) stainings in RGC axons with indicated treatments. (**B**) Area of RAB5 positive signal in axons. Dots represent vesicles. (*n* = 774 in CT, *n* = 782 in CHQ and *n* = 757 in CHQ + CHX, ***P  <  0.0001, *P = 0.0159, *n* ≥6 explants, 3 exp). (**C**) Percentage of RAB5-positive vesicles also positive for LC3B. Dots represent axons (CT: *n* = 39; CHQ: *n* = 37; CHQ + CHX: *n* = 31, *P = 0.0407, *n* ≥6 explants, 3 exp). (**D**) Images and quantification of RFP-RAB5 signal. Dots represent axons (CT: *n* = 23, CHQ: *n* = 21, *P* = 0.0053, *n* ≥4 explants, 2 exp). (**E**) Time-lapse sequence showing axon expressing RFP-RAB5 and GFP-LC3B treated with CHQ. Arrow indicates a RAB5 vesicle that transitions to LC3B-positive. Mean ± SEM. (**B**, **C**) Kruskal–Wallis test. (**D**) Mann–Whitney test. Scale bars = (**A**, **E**) 2 μm, and (**D**) 3 μm. Time is indicated as minutes (min). Source data are available online for this figure.

To further determine whether endosomal autophagy can be induced locally within axons and to assess the contribution of local protein synthesis to this process, we triggered endosomal stress pharmacologically using chloroquine (CHQ). CHQ accumulates within endosomal vesicles, where it becomes protonated. This leads to an osmotic imbalance, water influx, and subsequent endosomal swelling (Millarte et al, 2022). First, we examined endosomal morphology in axons before and after CHQ treatment. As expected, RAB5 immunostaining revealed an increase in the number of enlarged endosomes along the axon shaft as early as 20 min after treatment (Fig. 6A,B). Additional staining for LC3B showed enhanced colocalization with RAB5-positive vesicles, consistent with the local induction of endosomal autophagy (Fig. 6A,C). Interestingly, co-treatment with cycloheximide (CHX) did not prevent CHQ-induced endosomal swelling (Fig. 6A,B), but it did prevent the increase in LC3B colocalization with RAB5-positive endosomes (Fig. 6A,C). These results indicate that protein synthesis is required for LC3B association with stressed endosomes.

We then dynamically assessed how CHQ influences endosomal autophagy. Although enlarged endosomes were already present as a consequence of RAB5 overexpression, quantification revealed a further significant increase in the number of ring-like endosomes along the axon shaft following CHQ exposure (Fig. 6D). We then co-expressed RFP–RAB5 and GFP–LC3B and monitored both signals in real time. Live imaging confirmed a gradual expansion of RAB5-positive endosomes over time during CHQ treatment, consistent with progressive organelle swelling (Fig. 6E). Remarkably, this enlargement coincided with the emergence of LC3B signal on the endosomal membrane, which gradually expanded until the entire structure became decorated with LC3B. These enlarged endosomes were frequently observed in close proximity to additional LC3B-positive vesicles (Fig. 6E). Such dynamics are reminiscent of the autophagic response described after plasma membrane injury (Sønder et al, 2021) and supported the notion that, upon stress, RAB5-positive endosomes in axons can rapidly and locally transition to LC3B-positive compartments.

### *lc3b* mRNA localization to damaged organelles extends beyond early endosomes

**Figure 7:**
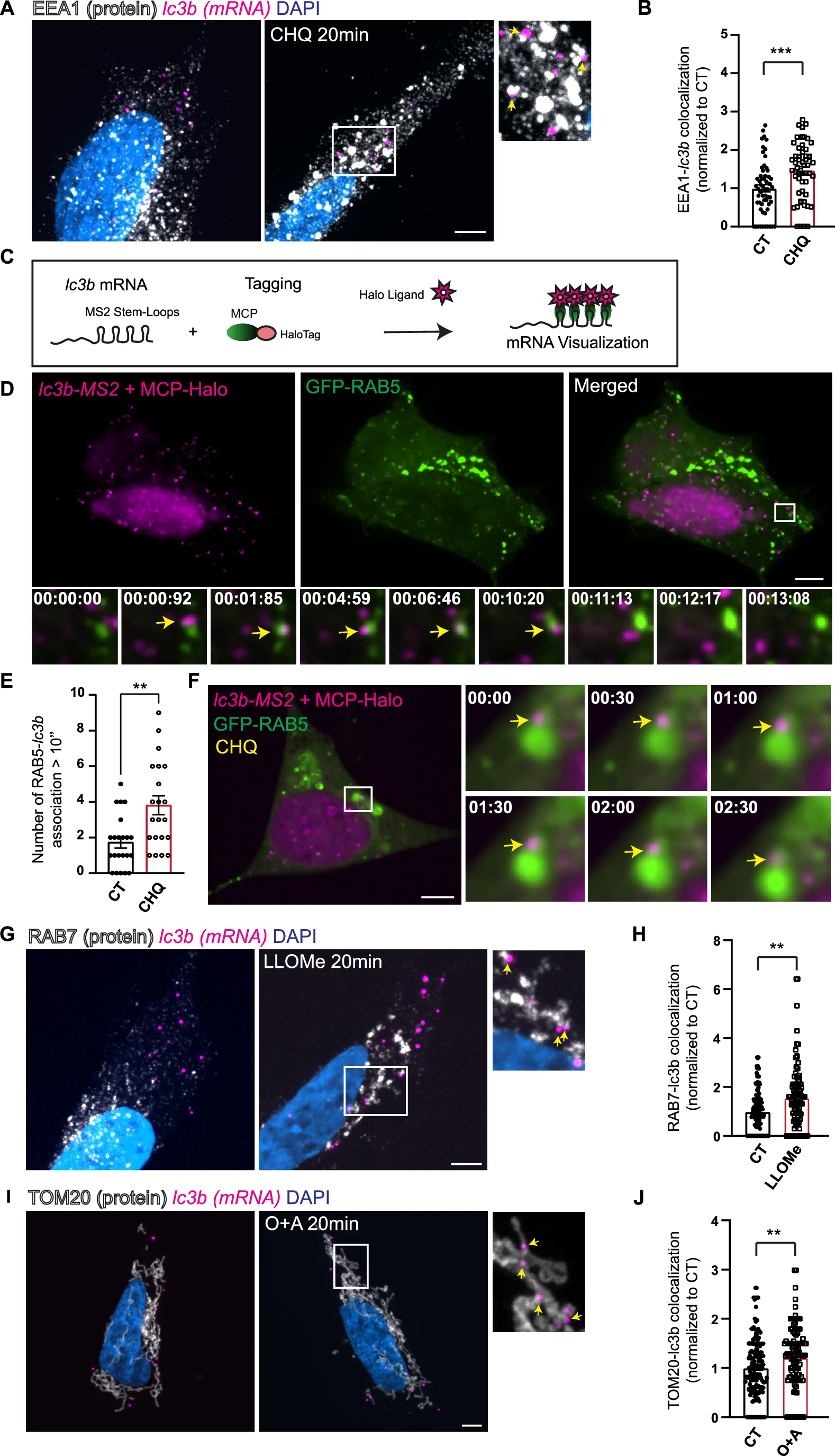
*lc3b* mRNA is recruited on damaged organelles upon organelle-specific stress. (**A**) smFISH images of *lc3b* mRNAs (in magenta) stained against EEA1 (in white) in SH-SY5Y cells treated with control (CT) or 50 μM chloroquine (CHQ) for 20 min. Arrows indicate mRNA signal colocalizing with EEA1 signal. A magnified view of the highlighted region is shown in the inset; the inset image has been rotated by 90°. (**B**) Colocalization analysis. Dots represent cells (CT: *n* = 58 cells; CHQ: *n* = 55 cells, *P* = 0.0005, 3 exp). (**C**) Scheme of the MS2 reporter system. (**D**) Example of a cell expressing *lc3b-MS2* reporter mRNA labeled by MCP-Halo (in magenta) and GFP-RAB5 (in green). Time-lapse sequence indicate association between the two signals (arrows). Time is indicated as min:sec:ms. A magnified view of the highlighted region is shown in the inset. (**E**) Quantification of the number of RAB5-lc3b mRNA reporter associations lasting for longer than 10 s. Dots represent cells (CT: *n* = 22 cells; CHQ: *n* = 21 cells, *P* = 0.0022, 3 exp.). (**F**) Example of a cell expressing *lc3b-MS2* reporter mRNA labeled by MCP-Halo (in magenta) and GFP-RAB5 (in green) treated with CHQ. Time-lapse sequence indicate association between the two signals (arrows). Time is indicated as min:sec. A magnified view of the highlighted region is shown in the inset; the inset image has been rotated by 90°. (**G**) smFISH images of *lc3b* mRNAs (in magenta) stained against RAB7 (in white) in SH-SY5Y cells treated with control (CT) or 250 μM LLOMe for 20 min. Arrows indicate mRNA signal colocalizing with RAB7 signal. A magnified view of the highlighted region is shown in the inset; the inset image has been rotated by 90°. (**H**) Colocalization analysis. Dots represent cells (CT: *n* = 116 cells; LLOMe: *n* = 96 cells, *P* = 0.0013, 4 exp). (**I**) smFISH images of *lc3b* mRNAs (in magenta) stained against TOM20 (in white) in SH-SY5Y cells treated with control or with 1 μM oligomycin A plus 1 μM antimycin (O + A) for 20 min. Arrows indicate mRNA signal colocalizing with TOM20 signal. A magnified view of the highlighted region is shown in the inset. (**J**) Colocalization analysis. Dots represent cells (CT: *n* = 162 cells; O + A: *n* = 104 cells, *P* = 0.0019, 4 exp). Mean ± SEM. (**B**, **E**, **H**, **J**) Mann–Whitney test. Scale bar = (**A**, **F**, **G**, **I**) 4 μm, and (**D**) 5 μm. Source data are available online for this figure.

We next asked whether *lc3b* mRNA could be actively recruited to impaired RAB5-positive endosomes, and whether this might represent a more general response to organelle stress. To start addressing these questions, we first tested whether EE-specific stress affects mRNA localization in SH-SY5Y cells. Cells were treated with CHQ for 20 min, and *lc3b* mRNA distribution was analyzed by smFISH in combination with EEA1 immunostaining. We observed a significant increase in the fraction of mRNAs colocalizing with EEA1-positive endosomes compared to control conditions (Fig. 7A,B), supporting rapid recruitment of *lc3b* mRNA to stressed EEs. To directly investigate *lc3b* mRNA dynamics, we employed the MS2 imaging system, which relies on the specific interaction between MS2 RNA stem-loop sequences and the MS2 coat protein (MCP) (Bertrand et al, 1998). *lc3b* mRNA was engineered to contain MS2 stem-loops and co-expressed with a construct encoding MCP fused to a tandem HaloTag. Upon addition of a fluorescent HaloTag ligand, this setup enabled visualization of the labeled transcripts in living cells (Fig. 7C). Under control conditions, we observed transient associations between GFP-RAB5-positive vesicles and *lc3b*-MS2 mRNA reporters throughout the cytoplasm. These associations were typically characterized by static or oscillatory co-movements (Fig. 7D). Importantly, we did not observe evidence of co-directed transport, further supporting the notion that EEs are not involved in the long-range cytoplasmic trafficking of *lc3b* mRNA. Upon CHQ treatment, the number of associations between *lc3b*-MS2 reporters and GFP-RAB5-positive endosomes significantly increased over the course of the 3-minute time-lapse recordings, consistent with enhanced recruitment of *lc3b* mRNA to stressed EEs (Fig. 7E). Under these conditions, stable associations between *lc3b*-MS2 reporters and RAB5-positive structures were also more frequently observed (Fig. 7F). Together, these findings suggest that organelle stress promotes and possibly stabilizes the association between *lc3b* mRNA and EEs.

Our previous findings showed that *lc3b* mRNA localization is not restricted to EEs, as significant colocalization was also observed with RAB7-positive late endosomes (Fig. 2C,D). To determine whether damage-induced mRNA recruitment extends to these compartments, we treated cells with L-leucyl-L-leucine methyl ester (LLOMe), which selectively disrupts late endosome/lysosome acidic vesicles through cathepsin C–dependent membrane permeabilization. We performed smFISH experiments in cells labeled for RAB7, and after 20 min of treatment, quantification revealed a significant increase in the fraction of *lc3b* mRNAs associated with RAB7-positive compartments (Fig. 7G,H).

We finally asked whether this response extends beyond the endosomal system to other membrane-bound organelles. Given the role of autophagy in mitochondrial quality control, we examined *lc3b* mRNA localization to mitochondria under stress conditions, using TOM20 as a mitochondrial marker. Induction of mitochondrial damage with antimycin A1 and oligomycin (O + A), which impair mitochondrial respiration and promote mitophagy, resulted in a significant increase in mRNA association with mitochondria after 20 min of treatment (Fig. 7I,J).

Collectively, these results indicate that *lc3b* mRNA is dynamically recruited to stressed organelles, including early and late endosomes as well as mitochondria, in response to damage.

## Discussion

In summary, we here report a rich set of different transcripts localized to endosomes, highlighting these organelles as platforms for mRNA localization. Consistent with prior studies, we identified multiple transcripts encoding endosomal proteins as well as regulators of lipid metabolism. A common feature across organelles is the localization of mRNAs encoding organelle-destined proteins at their surface. These associations often depend on ongoing translation, consistent with a broadly conserved mechanism of co-translational mRNA targeting (Chouaib et al, 2020). Similar observations have been reported for *EEA1* and *AP1S2* mRNAs at endosomes (preprint: Popovic et al, 2020, Chouaib et al, 2020), although the molecular mechanisms underlying this nascent peptide-mediated co-translational targeting remain unclear. We unexpectedly found the presence of *lc3b* mRNA associated with EEs, that led us to identify a link between autophagy and endosomal homeostasis in axons. The molecular factors governing this mRNA recruitment remain to be discovered. Unlike previously characterized endosome-associated transcripts, *lc3b* mRNA localization was not primarily dependent on active translation, suggesting instead that specific sequence or structural determinants within the transcript mediate this association. Previous studies support a post-transcriptional control of autophagy through regulation of *lc3b* mRNA localization and translation by RNA-binding proteins (RBPs). In epithelial cells, the RBP IMP1 keeps *lc3b* translationally repressed during homeostasis. Following cellular stress, however, IMP1 releases the mRNA for subcellular redistribution and translation (Parham et al, 2024). Additionally, SIGMA-1 receptor was recently identified as responsible for recruiting *lc3b* mRNA to the ER during AP formation. This process allows for the coupling of the protein’s synthesis, lipidation, and membrane insertion at specific sites (Knupp et al, 2024). Both IMP1 and SIGMA-1 have been reported to bind the 3’ UTR of the mRNA while mediating different effects. Our data suggest that the 3’UTR of *lc3b* is involved in the axonal targeting of the transcript, while the coding region alone is sufficient for its localization to endosomes once in the axonal compartment. Although these experiments do not exclude a modulatory role for the 3′UTR in endosomal recruitment, they indicate that distinct mechanisms regulate the recruitment of the mRNA to different organelles and/or in different contexts. We further found that *lc3b* mRNA can be recruited to late endosomes and mitochondria in a damage-dependent manner. Whether this recruitment drives local synthesis of LC3B and its incorporation into a nascent AP and/or directly into the organelle membranes remains unclear and may depend on the specific organelle and stress context. Nevertheless, these findings point to a broader cellular response in which organelle stress promotes the localized enrichment of autophagy-related transcripts.

Because of their complex morphology and long lifespan, neurons are particularly dependent on selective autophagy to prevent the accumulation of damaged organelles and protein aggregates in distal cellular compartments. Several forms of selective autophagy have been described in axons, including mitophagy and ER-phagy, and generally involve local AP biogenesis in proximity to the targeted organelle (Stavoe and Holzbaur, 2019). LC3B plays a central role in this process by promoting phagophore membrane expansion, recruiting cargo receptors and adaptor proteins through interactions with LC3-interacting region (LIR)-containing proteins, and facilitating AP trafficking and fusion with lysosomes for the degradation of their content (Sidibe et al, 2022). This study reports that constitutive endophagy occurs in distal axons, a process previously observed in cell lines (Fraser et al, 2019; Millarte et al, 2022). Moreover, our data also suggest a potentially faster, energy-efficient mechanism where LC3B directly binds endosomal membranes, acting as a molecular switch that promotes rapid local endosomal turnover. A similar principle has been described in non-canonical autophagy pathways such as LC3-associated phagocytosis (LAP), where LC3 conjugation onto single-membrane endocytic compartments promotes their maturation and fusion with lysosomes (Pena**-**Martinez et al, 2022). Although this process occurs only occasionally under basal conditions, it may become crucial during specific instances of stress or organelle damage. Given that enlarged endosomes induced by RAB5 overactivation have been linked to neuronal loss in AD (Pensalfini et al, 2020), our results may provide valuable insights into the pathogenic mechanisms associated with defective autophagic and endolysosomal trafficking in this disorder. Moreover, the observed response to local damage induced by chloroquine opened the possibility that stressors like reactive oxygen species, protein aggregates, or viral infections may similarly affect endosomes in axons. A comprehensive molecular understanding of endosomal clearance following damage could reveal how axons manage stress in health and disease and provide new perspectives for therapeutic interventions.

## Methods

**Table:** Reagents and tools table

Reagent/resource	Reference or source	Identifier or catalog number
**Experimental models**		
*Xenopus laevis*		
Human neuroblastoma SH-SY5Y cells	SIGMA	Cat#94030304
**Recombinant DNA**		
NES-APEX2	Lobingier et al, 2017	N/A
2xFYVE-APEX2	Lobingier et al, 2017	N/A
RAB5A-APEX2	Del Olmo et al, 2019	N/A
peGFP-hRAB5	This paper	N/A
peGFP-hRAB5^S34N^ (DN)	Addgene	Cat#28045
pCS2-mRFP-xRab5	Gift from Holt lab	N/A
pCS2-mRFP-xRAB5^S35N^ (DN)	This paper	N/A
T7-HA-mBiCD-FRB	Gift from Farias lab	N/A
pCS2-GFP-2xFKBP-RAB5	This paper	N/A
pCS2-GFP-xLC3b.S-3’UTR	This paper	N/A
pCS2-GFP-xLC3b.S	This paper	N/A
pCS2-scFv-sfGFP	Cioni et al, 2019	N/A
pCS2-xLC3b.S-24xSunTag-*3’UTR*	This paper	N/A
pCS2-hATG7CS	This paper	N/A
2xMCP-2xHalo	Addgene	Cat#104999
5’UTR-hLC3b-3’UTR-24xMS2	This paper	N/A
**Antibodies**		
Mouse monoclonal anti-RAB5 (D-11)	Santa Cruz	Cat#sc-46692
Rabbit monoclonal anti-RAB5A	Abcam	Cat#ab218624
Rabbit polyclonal anti-LC3b	Proteintech	Cat#14600-1-AP
Mouse monoclonal antibody anti-EEA1	Thermo Fisher Scientific	Cat#MA5-31575
Rabbit monoclonal anti-RAB7	Abcam	Cat#ab137029;
Rabbit polyclonal anti-TOM20	Proteintech	Cat#11802-1-AP;
Mouse monoclonal anti-GFP	Abcam	Cat#Ab1218;
Rabbit polyclonal anti-biotin	Abcams	Cat#ab53494;
Mouse anti-Puromycin(12D10)	Sigma	Cat#MABE343
Goat anti-rabbit Alexa Fluor 488	Abcam	Cat#ab150077;
Goat anti-mouse Alexa Fluor 488	Abcam	Cat#ab150113;
Goat anti-mouse Alexa Fluor 647	Thermo Fisher Scientific	Cat#A21235;
Goat anti-rabbit Alexa Fluor 594	Abcam	Cat#ab150080;
**Oligonucleotides and other sequence-based reagents**		
Primers for molecular cloning	This study	Methods section
Primers for qPCR	This study	Methods section
Primers for PCR	This study	Methods section
ViewRNA® RNA probes	Thermo Fisher Scientific	CVX-1 and VX-1
**Chemicals, enzymes and other reagents**		
JetPRIME transfection reagent	Polyplus Transfection	Cat#114-15
Lipofectamin2000	Thermo Fisher Scientific	Cat#11668019
Antibiotic–antimycotic	Thermo Fisher Scientific	Cat#15240062
Tricaine methanesulfonate	Sigma	Cat#A5040
L-15 medium	Gibco	Cat#11415-049
Poly-L-lysine	Sigma	Cat#P1274
Laminin	Sigma	Cat#L2020
Biotin Phenol	Chemodex	Cat#B0270
Dynabeads MyOne Streptavidin T1	Invitrogen	Cat#65601
Yeast RNA	Invitrogen	Cat# AM7118
Proteinase K	Thermo Fisher Scientific	Cat#25530-049
RiboLock RNase Inhibitor	Thermo Fisher Scientific	Cat# EO0382
RNaseA	Sigma	Cat#R5000
Cyanine5-UTP	PerkinElmer	Cat# NEL58300
A/C heterodimerizer (Rapalog)	Takara	Cat#635056
Chloroquine	Sigma	Cat# C6628
L-Leucyl-L-Leucine methyl ester(LLOMe)	Cayman	Cat#16008
OligomycinA	Sigma	Cat#75351
Antimycin	Sigma	Cat#A8674
Cycloheximide	Sigma	Cat#C1988
Puromycin	Sigma	Cat#P8833
Fluorsave mounting medium	Sigma	Cat#345789
Albumin from Bovine Serum (BSA), Alexa Fluor™ 647 conjugate	Thermo Fisher Scientific	Cat#A34785
**Software**		
Graphpad Prism	GraphPad Software, 10.6.0	https://www.graphpad.com
FIJI	v2.14/1.54 f	https://fiji.sc
NIS elements AR software	Nikon	
**Other**		
NEBuilder® HiFi DNA Assembly Cloning Kit	New England Biolabs	Cat# E5520S
Q5® Site-Directed Mutagenesis Kit	New England Biolabs	Cat# E0554S
Wizard SV-gel Clean up system	Promega	Cat#A9281
mMESSAGE mMACHINE SP6 transcription kit	Thermo Fisher Scientific	Cat# AM1340
Poly(A) Tailing Kit	Invitrogen	Cat# AM1350
RNeasy Mini Kit	Qiagen	Cat#74104
RNA Clean and Concentrator kit	Zymo Research	Cat# R1013
RNeasy plus mini kit	Qiagen	Cat#74136
HiScript III 1st Strand cDNA Synthesis Kit	Vazyme	Cat# R312
Luna Universal qPCR Master Mix	New England Biolabs	Cat# M3003
ViewRNA® Cell Plus Assay kit	Invitrogen	Cat#881900099
duolink in situ detection reagents red	Sigma	Cat#DUO92008
duolink in situ PLA probe anti-rabbit min	Sigma	Cat#DUO92005
duolink in situ PLA probe anti-mouse plus	Sigma	Cat#DUO92001

### Mammalian cell lines and culture conditions

Human neuroblastoma SH-SY5Y cells (Sigma, 94030304) were cultured in RPMI-1640 Medium (Sigma, R8758) supplemented with 1% MEM non-essential amino acids (EuroClone, ECB3054D), 1% penicillin/streptomycin (EuroClone, ECB3001D) and 10% heat inactivated fetal bovine serum (EuroClone, ECS0180LH). Cells were split twice a week, using 1× trypsin-EDTA (EuroClone, ECB3052D) and used up to passage 35. All cells were maintained in a 5% CO_2_, 37 °C humidified environment.

### Cell transfection

SH-SY5Y were transfected using jetPRIME transfection reagent (Polyplus Transfection, 114-15) according to manufacturer’s instructions, 1–2 days after plating. Transfection was performed overnight, and cells were analyzed within 48 h. For rapalog-induced heterodimerization experiments, SH-SY5Y cells were transfected with pCS2-GFP-2xFKBP-RAB5 and t7-HA-BICD-FRB DNA plasmids at a 1:2 ratio. To generate stable lines expressing APEX2 constructs, SH-SY5Y cells were plated 1 or 2 days before transfection. The day after transfection, culture medium was substituted completely with new medium supplemented with 0.5 mg/mL geneticin (Gibco, 10131035). Antibiotic selection was kept for 10 days, after which the concentration of geneticin in the medium was decreased to 0.1 mg/mL. For MS2 system experiments, SH-SY5Y cells were transfected with eGFP-hRAB5, LENTI-5′-hLC3B-3′_MS2, and LENTI-NLS-HA-2xMCP-2xHalo plasmids at a 40:1 ratio of MS2-tagged mRNA to MCP-Halo. Transfections were performed using Lipofectamine™ 2000 (Thermo Fisher Scientific), according to the manufacturer’s instructions, 1 day after plating. Forty-seven hours after transfection, cells were incubated with 50 nM HaloTag ligand JF635 (Lavis Lab, Lot: NF-3-137) for 1 h. Cells displaying visible MS2 reporter puncta were selected for imaging.

### *Xenopus laevis* embryos maintenance

*Xenopus laevis* embryos were obtained by in vitro fertilization, raised in Modified Barth’s Saline (MBS) 0.1× (88 mM NaCl, 1 mM KCl, 2.5 mM NaHCO_3_, 5 mM HEPES, 1 mM MgSO_4_, 0.7 mM CaCl_2_ in H_2_O) at 14–20 °C and staged according to Nieuwkoop and Faber (1994). All animal experiments were approved by the local Institutional Animal Care and Use Committees (IACUC) and the Italian Ministry of Health (under the authorization number 652/2021-PR).

### Blastomere microinjection

Two-cell stage *Xenopus* embryos were de-jellied by replacing MBS 0.1× with 2% L-cysteine (Sigma, W326305) in MBS 1× for 2 min or until visible removal of jelly-like coating, and then washed 3–5 times in MBS 0.1×. Needles for blastomere microinjection were prepared by pulling glass capillaries (outer diameter: 1.0 mm; inner diameter: 0.5 mm, Harvard Apparatus 30-0032) with a needle puller set at 62 and 68 °C. Needles were loaded with in vitro synthesized mRNA mixtures by using microloader pipette tips and mounted on a micromanipulator-held capillary holder connected to a pressurized microinjector set at 100 psi. Capillary tip was broken with fine forceps and pulse duration of the microinjector was adjusted to deliver 5 nL of injection mixture per pulse, calibrated by measuring drop diameter in mineral oil. Four-cell stage embryos were transferred in a gridded dish containing Ficoll solution [4% Ficoll (Merck, F4375), 1% antibiotic–antimycotic, in MBS 0.1× and adjusted to pH 7.5], and the dorsal blastomeres of the embryos were microinjected. Following injection, embryos were left in Ficoll solution for at least 1 h at 20–22 °C, then washed three times in MBS 0.1× and moved in an incubator set at the desired temperature (14 °C or 22 °C according to the development speed needed).

### Primary *Xenopus* retinal cultures

Under sterile conditions, stage 35/36 embryos of both sexes were washed three times in MBS 0.1× supplemented with 1% antibiotic–antimycotic (Life Technologies, 15240062), anaesthetized for at least 1 min in MS-222 solution [0.04% tricaine methanesulfonate (Sigma, A5040), 1% antibiotic–antimycotic in MBS 0.1×] and transferred to a Sylgard dish containing MS-222 solution for dissection. Eye primordia were dissected using 0.1 mm Minutien pins (FST, 26002-10) attached to pin holders and kept in *Xenopus* culture medium [60% L-15 medium (Gibco, 11415-049), 1% antibiotic–antimycotic, in H₂O]. Dissected eyes were mounted lens-up on coverslips or Mattek (P35G-1.5-14-C) previously coated with 10 μg/mL poly-L-lysine (Sigma, P1274) in sterile H₂O for at least 2 h or overnight at room temperature, washed three times with sterile H₂O, allowed to dry and re-coated with 10 μg/mL laminin (Sigma, L2020) in L-15 medium for 1–4 h. Eye explants were then cultured in *Xenopus* culture medium at 20 °C for 18–24 h. For live imaging experiments, culture medium was prepared using L-15 medium without phenol red (Gibco, 21083-027). For axon-specific analysis, eye explants were plated on the top compartment of six-well hanging inserts with 1 mm membrane pores (Greiner Bio-One, 657610). The bottom side of the membrane was coated as previously described for *Xenopus* primary cultures, while the top side was coated with poly-L-lysine only. In total, 250 eyes were plated in each chamber and cultured for 72 h.

### Constructs

APEX2-NES and 2xFYVE-APEX2 plasmids have been previously generated (Lobingier et al, 2017) and were kindly donated by von Zastrow Lab (University of California, San Francisco, USA). APEX2-RAB5 construct has been previously generated (Del Olmo et al, 2019) and was kindly donated by Steve Jean Lab (Université de Sherbrooke, Sherbrooke, QC, Canada). GFP-hRAB5 DN (Addgene #28045) and hRab5tdTomato (Addgene #58126) were purchased from Addgene. peGFP-hRAB5 was obtained by point mutation with Q5® Site-Directed Mutagenesis Kit (E0554S). The constructs pCS2-mRFP-xRab5, pCS2-eGFP-xRab5, pCS2-mRFP-xRab7 were previously reported. pCS2-mRFP-xRAB5S35N (DN) was obtained by introducing a point mutation in pCS2-RFP-xRab5 with Q5® Site-Directed Mutagenesis Kit (E0554S) to obtain S35N amino acid change. Cloning experiments have been performed using NEBuilder® HiFi DNA Assembly Cloning Kit (E5520S). T7-HA-BiCD-FRB was kindly donated by Farias Lab (Utrecht University, The Netherlands). pCS2-GFP-2xFKBP-RAB5 was obtained by PCR of 2xFKBP from GFP-2xFKBP-RTN4a (Farias lab) and inserted in pCS2-EGFP-RAB5 (Cioni et al, 2019) digested by XhoI. pCS2-GFP-xLC3b.S-3’UTR was obtained by PCR of xLC3b.S-3’UTR from pCMVSPORTxLC3b.S-3’UTR (Horizon Discovery MGC68744) and inserted in pCS2-eGFP backbone digested by XhoI, pCS2-GFP-xLC3b.S was obtained by PCR of xLC3b.S from pCMVSPORTxLC3b.S-3’UTR (Horizon Discovery MGC68744), and inserted in pCS2-eGFP backbone digested by XhoI, pCS2-scfvGFP construct was previously reported (Cioni et al, 2019). In all, 24-SunTag tandem repeats were obtained from *lmnb2 5’UTR-24xSunTag-lmnb2 CDS-lmnb2 3’UTR* (Cioni et al, 2019) and inserted into pCS2+ vector. xLC3b.S CDS was cloned into the 5’ end of the SunTag repeats, while the 3’UTR was cloned into 3’ end of the SunTag repeats, generating the pCS2-xLC3b.S-Suntag-3’UTR. pCS2-xSqsm1-eGFP was obtained by PCR of xSqstm1 from *Xenopus* cDNA and inserted in pCS2-eGFP (donated by Holt Lab) digested by AgeI. pCS2-hATG7CS was obtained by PCR of hATG7CS from pCMV-hATG7CS (addgene # 87868) and inserted in pCS2+ digested by BamHI. The constructs pCS2-eGFP and pCS2-mRFP were previously reported. The *hLC3B* mRNA with 5’ and 3’UTRs was amplified by PCR from SHSY5Y cDNA and inserted in a Lentiviral vector containing 24xMS2 loops. The 2xMCP-2xHalo plasmid was purchased by Addgene (#104999).

### in vitro mRNA synthesis

In total, 15 μg of pCS2+ plasmids were linearized with 3U enzyme/μg DNA of Not1 HF (NEB, R3189L) in rCutSmartTM Buffer (NEB, B6004S) at 37 °C for 3 h. The linearized plasmid was checked by electrophoresis on a 1.2% Agarose gel (Merck, A9539) in 1× TAE (Merck, T9285) and purified with Promega Wizard SV-Gel and PCR Clean-Up System (Promega, A9281). mRNAs were then synthesized using the mMESSAGE mMACHINE SP6 transcription kit (ThermoFisher, AM1340) according to manufacturer’s instructions and polyadenylated with the Invitrogen poly(A) Tailing Kit (Invitrogen, AM1350). Polyadenylated mRNAs were then purified with the RNeasy Mini Kit (Quiagen, 74104). Polyadenylation was checked by running RNAs on a 1.2% Agarose gel in 1× TAE and RNA concentrations were quantified using a Nanodrop ND-1000 Spectrophotometer (Euroclone). RNA samples were then stored at −80 °C. For in vitro synthesis of cy5-labeled mRNAs, 1 μL of Cyanine5-UTP (PerkinElmer, NEL583) was added during mMESSAGE mMACHINE SP6 transcription kit reaction.

### APEX2 biotinylation

SH-SY5Y cells were incubated in 500 μM biotin phenol (Chemodex, B0270) in their original medium for 30 min. To favor a proper diffusion, biotin phenol was first diluted in half of the medium and then added onto the cells. 1 mM H₂O₂ (Sigma, H1009) was added in the same way to the cells and left in incubation for 2 min with gentle agitation. To block APEX2 reaction all medium was then removed, and cells were washed with quencher solution [10 mM Sodium Azide (Sigma, S2002), 10 mM Sodium Ascorbate (Sigma, A4034), 5 mM Trolox (Sigma, 238813) in PBS]. For immunofluorescence protocols, cells were washed three times for 5 min with warm quencher solution, then fixed and stained as described below. For immunoprecipitation of biotinylated RNAs, cells were quickly washed one time with ice-cold quencher solution and then three times for 5 min with ice-cold quencher solution without Sodium Azide.

### Immunoprecipitation of biotinylated RNAs

Following biotinylation, cells were scraped in 600 μL of buffer RLT Plus supplemented with 10 μl/ml β-mercaptoethanol (Sigma, M6250). Once collected, samples were mechanically lysed passing them three times through a 25-gauge needle, fitted to a RNase-free syringe. Homogenized samples were then processed following RNeasy Plus Mini Kit manufacturer’s instructions (QIAGEN, 74134). After RNA extraction, RNA concentration and purity were quantified using a Nanodrop ND-10000 Spectrophotometer (Euroclone). To immunoprecipitate bioinylated RNAs, 17 μl of magnetic beads were used for 40 μg of RNA sample. Before use, beads were washed three times in B&W buffer (100 μL per 10 μL beads, 5 mM Trizma pH 7.5, 0.5 mM EDTA, 1 M NaCl, 0.1% Tween-20, in water), two times in solution A (0.1 M NaOH, 0.05 M NaCl, in water) and one time in solution B (0.1 M NaCl in water). Beads were finally resuspended in PBS + 0.05% Tween-20 (Sigma, P1379) and blocked with yeast RNA (100 μg per 100 μl beads, Invitrogen, AM7118) for 1 h at 4 °C. After blocking, beads were resuspended in 150 μl of solution B and then incubated with 125 μl of RNA previously diluted in water for 2 h at 4 °C on a rotator. Beads were then washed three times in B&W buffer (100 μL per 10 μL beads), then resuspended in 54 μl of water, adding 33 μl of 3× digestion buffer (6% N-Lauryl sarcosine sodium solution, 30 mM EDTA, 1.5 mM DTT, in PBS 3×), 10 μl of proteinase K solution (ThermoFisher Scientific, 25530-049) and 3 μL of RiboLock RNase Inhibitor (ThermoFisher Scientific, EO0382), and incubated for one hour at 42 °C and then one hour at 55 °C with shaking (Eppendorf, ThermoMixer C). Supernatants were finally collected and cleaned up with RNA Clean and Concentrator kit – 5 (Zymo Research, R1013), following manufacturer’s instructions.

### Dotblot

For RNAse-treated samples, 3 μg of RNA were diluted in 99 μL of water, adding 1 μL of RNAse A (100 μg/mL, Sigma, R5000) in the lid of the tube. To start the reaction at the same time, all tubes were spined together and then incubated at 37 °C for 30 min. Digested RNA was purified using RNA Clean and Concentrator Kit according to manufacturer’s instructions, and samples were finally eluted in 6 μL. In total, 2 μL then were spotted on a nitrocellulose membrane (Amersham, 10600002) and allowed to dry at room temperature. After few minutes, RNA was crosslinked to the membrane using a UV Stratalinker 2400 (Stratagene) and 2500 μJ Energy. The membrane was then washed for 5 min with PBS-T (PBS + 0.2% Tween-20) at room temperature and then incubated with 1:1000 HRP-conjugated streptavidin (Invitrogen, S911) diluted in PBS-T for 30 min at room temperature. After incubation, the membrane was washed three times for 10 min with PBS, and incubated for 2 min in the dark with SuperSignal west Pico PLUS Chemiluminescent Substrate (ThermoFisher Scientific, 34577). Finally, signal was detected using a Chemidoc MP Imaging System (BIORAD).

### RNA extraction and RT-qPCR

SH-SY5Y were washed in PBS and then scraped and lysed in RLT plus buffer with β-mercatopethanol. *Xenopus* embryos eyes were washed once in PBS and then lysed in RLT plus buffer with β-mercaptoethanol. For tissue disruption, eyes were mechanically disrupted by pipetting. Samples were then centrifuged at max speed for 15 min and supernatants were collected. RNA extraction was performed by using RNeasy plus mini kit (Qiagen, 74136) following manufacturer’s instructions. RNA concentrations were measured with Nanodrop ND-10000 Spectrophotometer (Euroclone) and cDNA was prepared by retrotranscription of equal amount of RNA from different samples using the HiScript III 1st Strand cDNA Synthesis Kit (Vazyme, R312). cDNA samples were diluted (from 1:2 to 1:5) and used to prepare triplicate reactions for quantitative RT-PCR using Luna Universal qPCR Master Mix according to manufacturer’s instructions (NEB, M3003). Plates were centrifuged shortly and run on a CFX Connect Real-Time PCR System (Eppendorf).

For compartmentalized analysis, somatodendritic fraction was isolated by detaching retina explants from membrane through gentle pipetting over them. Eyes explants were then collected, quickly washed in PBS and lysed in RLT plus buffer with β-mercaptoethanol. Before isolation of the axonal fraction, the upper part of the membrane was scrubbed with UV-cleaned cotton buds for ten times, alternating the direction, to remove any residual cells. Membrane was then cut out from the insert with a scalpel, quickly washed in a drop of PBS, placed on a drop of RLT plus buffer with β-mercaptoethanol and incubated at 4 °C with slow shaking for 10 min. RNA extraction was performed using RNeasy plus mini kit (Qiagen, 74136) for somatodendritic fraction and RNeasy plus micro kit (Qiagen, 74034) for axonal fraction. cDNA was prepared by retrotranscription of equal volumes of somatodendritic and axonal RNA using LunaScript RT SuperMix Kit (NEB, E3010). Then, PCR was performed using Phusion high fidelity DNA polymerase (NEB, M0530L) and run with a Mastercycler X50s (Eppendorf) for 35 cycles. PCR amplicons were then examined by electrophoresis on a 2,5% agarose gel in TAE 1× buffer with 1:10,000 SyBR Safe DNA Gel Stain (Invitrogen, S33102) (loading buffer: BioLabs, B7024A; DNA ladder: NEB, 1 kb, N3232S).

The following primers were used for RT-qPCR:

**Table:** 

Species	Gene	Fwd
Human	*MAP1LC3A*	FWD: ATGTCAACATGAGCGAGTTG REV: TTCTCCTGCTCGTAGATGTC
Human	*MAP1LC3B*	FWD: CCGGTGATAATAGAACGATACAA REV: CCTGATTAGCATTGAGCTGTAA
Human	*SQSTM1*	FWD: AATTTCCTGAAGAACGTTGG REV: GGAACTCTCTGGAGAGACG
Human	*ACTB*	FWD: CCTTCTACAATGAGCTGCG REV: GGATAGCAACGTACATGGC
Xenopus	*actb.L*	FWD: CCAGAAGAACACCCAGTGCT REV: CAGGGACAACACAGCTTGGA
	*EGFP*	FWD: GAAGGCTACGTCCAGGAG REV: ATGCCGTTCTTCTGCTTGTC

The following primers were used for PCR analyses:

**Table:** 

Gene	Primers
*actb.L*	FWD: CCAGAAGAACACCCAGTGCT REV: CAGGGACAACACAGCTTGGA
*glur1*	FWD: GGGATTGGCCATGCTTGTTG REV: CATTCCTGCACTGTGGCTCA
*map2*	FWD: CATCCTTGCCAAGACCTTCCTC REV: CGACCTGGAGATTGGGTGATG
*lc3b.L*	FWD: GCAACTGCCAGTCCTCGATA REV: GAGTGGATACGCTCACCATG
*lc3a.L*	FWD: AGAGCGATACAAGGGGGAGAAAC REV: CATGCTGTGCTGGTTTACAAGC

### Pharmacological treatments

For rapalog-induced heterodimerization experiments, SH-SY5Y cells were treated with 100 nM rapalog (A/C heterodimerizer, Takara, 635056) for up to 2 h. To block protein synthesis, RGC axons were treated with 10 μM cycloheximide (Sigma, C1988) or with 100 μM puromycin (Sigma, P8833). To induce organelle-specific stress, cells were treated with 50 μM chloroquine (CHQ, Sigma, C6628), 250 μM L-Leucyl-L-Leucine methyl ester (hydrochloride) (LLOMe, Cayman, 16008), or 1 μM oligomycin A (Sigma, 75351) plus 1 μM antimycin (Sigma, A8674). Control conditions were treated with equal amounts of H_2_O or DMSO according to drug diluent.

### Immunocytochemistry

SH-SY5Y cells were briefly washed in warm PBS and then fixed in warm 4% paraformaldehyde (ThermoFisher Scientific, 28908), 4% sucrose (Sigma, S7903) in PBS for 20 min at room temperature. Coverslips were washed three times with PBS for 10 min. Cells were permeabilized for 4 min in 0.1% Triton-X-100 (Sigma, X-100) in PBS and then washed three times with PBS for 10 min. Cells were blocked in 2.5% goat serum (Gibco, 16210-064) in PBS for at least 45 min. Primary antibody was diluted in 2.5% goat serum in PBS and incubated overnight at 4 °C. Coverslips were washed three times with PBS for 10 min, and then incubated with an appropriate secondary antibody for 1 h at room temperature. Coverslips were washed three times with PBS for 10 min and, if needed, 1:10,000 DAPI (Thermofisher D1306) was added in PBS during the first wash. Coverslips were mounted on microscope slides (ThermoFisher Scientific, J1800AMNZ) using a drop of Fluorsave mounting medium (Merck, 345789), then dried overnight at room temperature and stored at 4 °C.

*Xenopus* RGC axonal cultures were fixed for 20 min by adding an equal amount of volume of fixing solution [4% paraformaldehyde (Life Technologies, 28908), 15% sucrose (Sigma, S7903) in PBS] directly onto cell culture medium, and then washed six times with wash buffer [0.001% Triton-X-100 (Sigma, X-100) in PBS] for a total time of 30 min. Axons were permeabilized using 0.1% Triton in PBS for 5 min and then washed three times for 10 min with wash buffer. Blocking was performed using 5% goat serum (Gibco, 1610-064) in wash buffer for at least 45 min. Primary antibodies were diluted in blocking buffer and incubated at 4 °C overnight. Coverslips were then washed three times for 10 min with wash buffer, incubated with an appropriate secondary antibody for 1 h at room temperature, washed three times for 10 min with wash buffer and finally mounted on microscope slides using Fluorsave mounting medium. Slides were left overnight at room temperature to dry and then stored at 4 °C.

The following primary and secondary antibody were used for immunofluorescence:

**Table:** 

Antibody	Dilution	Source and identifier
Mouse monoclonal anti-RAB5 (D-11)	1:50	Santa Cruz Biotechnology, sc-46692
Rabbit monoclonal anti-RAB5A	1:500	Abcam, ab218624
Rabbit polyclonal anti-LC3	1:300	ProteinTech, 14600-1-AP
Mouse monoclonal anti-EEA1	1:500	ThermoFisher, MA5-31575
Rabbit monoclonal anti-RAB7	1:500	Abcam, ab137029
Rabbit polyclonal anti-TOM20	1:500	Protein Tech, 11802-1-AP
Mouse monoclonal anti-GFP	1:500	Abcam, ab1218
Rabbit polyclonal anti-biotin	1:500	Abcam, ab53494
Goat anti-rabbit Alexa Fluor 488	1:1000	Abcam, ab150077
Goat anti-mouse Alexa Fluor 488	1:1000	Abcam, ab150113
Goat anti-mouse Alexa Fluor 647	1:1000	ThermoFisher, A21235
Goat anti-rabbit Alexa Fluor 594	1:1000	Abcam, ab150080

### Puro-PLA

*Xenopus* RGC axons were incubated for 5 min with 2 µM puromycin. Subsequently, the axons were fixed in PFA-sucrose, permeabilized with PBS containing 0.1% Triton-X-100, and blocked in PBS supplemented with 4% goat serum, following standard immunocytochemistry procedures. The axons were then incubated for 2 h at room temperature with primary antibodies diluted in 4% goat serum: mouse anti-puromycin (1:500, MABE343, Sigma-Aldrich) and rabbit anti-LC3. Finally, Duolink puro-PLA reagents (Sigma DUO92002) were applied according to the manufacturer’s protocol.

### Single molecule fluorescence in situ hybridization (smFISH)

SH-SY5Y cells or *Xenopus* RGC primary cultures were fixed and permeabilized according to respective protocols, but all steps were performed under RNase free conditions. Solutions for fixation, permeabilization and washing were supplemented with RNase inhibitors from ViewRNA® Cell Plus Assay kit (Invitrogen, 881900099), prepared with RNase free PBS from kit, RNase free water (Invitrogen, 10977-035), and filtered through a 0.22 μm PES filter. Then, smFISH was performed according to ViewRNA® Cell Plus Assay kit manufacturer’s instructions. Cells were incubated with probes for 2 h at 40 °C, and subsequently with pre-amplifier, amplifier, and label solution each for 1 h at 40 °C in a ProBlot hybridization oven (Labnet). Between each step cells were washed with wash buffer from the kit, five times for SH-SY5Y cells and three times for axons. If needed, after smFISH protocol was completed, cells were immunostained as previously described. Otherwise, cells were washed for 10 min in PBS and coverlips were mounted, dried, and stored at 4 °C.

### Electron microscopy

RGC axons were fixed for 1 h at room temperature (RT) by adding an equal volume of the fixing solution (8% paraformaldehyde electron microscopy grade, 0.1% glutaraldehyde and 15% sucrose in 0.1 M HEPES buffer) to the culture media. Following three washes with 1× PBS, the samples were incubated for 10 min in 50 mM glycine, then permeabilized for 10 min with 0.25% saponin and 0.1% BSA in 1× PBS. Next, they were blocked for 1 h in blocking buffer (5% goat serum, 0.2% BSA, 0.1% saponin, 50 mM NH4Cl, 20 mM PO4 buffer, 150 mM NaCl). After blocking, the samples were incubated with the anti-GFP primary antibody (AB 6556) for 1.5 h at room temperature. Following washes in a solution containing 0.1% BSA and 0.1% saponin in 1× PBS, they were incubated for 1 h at room temperature with nanogold-conjugated secondary antibodies (Nanoprobes). Finally, the samples were fixed with 1% glutaraldehyde for 30 min, and the nanogold was enlarged using a gold enhancement solution (Nanoprobes), following the manufacturer’s instructions. Samples were then post-fixed in a solution containing 1% OsO₄, 1.5% K₄Fe(CN)₆, and 0.1 M sodium cacodylate (OsO₄ 75632, Sigma-Aldrich; potassium ferricyanide 702587, Sigma-Aldrich) for 1 h at 4 °C, protected from light. After post-fixation, samples were washed three times for 5 min each with 0.1 M cacodylate buffer, followed by five washes with distilled water. The samples were stained with 0.5% uranyl acetate at 4 °C overnight, protected from light. Following five additional washes with distilled water, samples were dehydrated in ethanol at the following concentrations (5 min per step): 30%, 50%, 70%, 80%, 90%, and 96%, followed by three washes of 5 min each in 100% ethanol. Samples were then infiltrated with a 1:1 mixture of ethanol and epoxy resin and placed on a shaker for 2 h at RT. This was followed by two changes of pure epoxy resin (1 h per change) at RT. Finally, the samples were embedded in fresh epoxy resin and polymerized at 45 °C overnight, followed by 24 h at 60 °C. Ultrathin sectioning (70 nm) was performed using a Leica EM UC6 ultramicrotome. Sections were collected onto formvar-coated slot grids. The thin sections were contrasted with 2% aqueous uranyl acetate for 5 min, followed by three washes in filtered distilled water (1 min each). The grids were then incubated in Sato lead stain for 2 min, followed by three rinses with distilled water and drying with Whatman filter paper. Images were collected using a FEI Tecnai-120 transmission electron microscope.

### Image acquisition

Fixed or live cells were imaged with a Nikon CSU-X1 Spinning Disk, Nikon TE2 inverted microscope equipped with an ORCA-Flash4.0 digital camera C13440 Hamamatsu and 100X oil objective (1.4 NA). Acquisition was performed using NIS Elements acquisition software (Nikon). For quantitative analysis of signal intensity, exposure time and laser power were kept constant and adjusted to keep signal below pixel saturation for all images belonging to the same experiment. During live imaging experiments, focus was maintained with a Nikon TiE Perfect Focus System and cells were kept under appropriate cell culture conditions using a stage incubator (OKOlab). For axonal imaging, isolated axons with noncollapsed growth cones were selected. Axons from at least 2–3 different retinal explants, and therefore different embryos, were analyzed in each experiment.

### Image analyses

All analyses were performed using the NIS elements AR software (Nikon), except for analyses of mean pixel intensities and colocalization that were performed on ImageJ/Fiji software. When blind analyses were performed, image names were blinded using the “Blind Analysis Tool” plugin of ImageJ/FiJi. Representative images were denoised by NIS elements AR software.

#### mRNA-organelle colocalization analyses

Isolated or manually separated cells with at least two probe puncta were considered for the analyses. For a consistent visualization of signal intensities between different images and conditions, LUTs settings were established a priori for each analyzed channel, and the same settings were applied to all images. When treatments were performed, images were analyzed in blind. The total amount of probes puncta was quantified in each cell, considering all the focal planes of the image. Then, colocalization analysis was performed by visual inspection of signal overlap, considering colocalization as a 25% minimum overlap. Probe puncta colocalizing with organelle marker signal were counted and the percentage of mRNAs localized on organelle for each cell was quantified. The same method was used for analysis on *Xenopus* axons. When the mRNA signal was too low, probes puncta located in proximal axons, distal axons, or growth cones were included in the analysis, and the colocalization percentage was measured by considering all mRNAs detected in each experiment.

#### mRNA spatial distribution in cells

To analyze the spatial distribution of mRNA in SH-SY5Y cells, which are slightly elongated rather than round, we first assessed cell morphology to establish the cell elongation direction. An ROI was automatically generated on the nuclear DAPI signal using an auto-detect tool. A reference axis was then drawn through the centroid of the ROI, perpendicular to the cell elongation axis. Using this reference axis, we measured the distances of individual mRNAs from the nucleus within the analyzed portion of the cell. We also measured the maximum distance from the nucleus to the cell membrane along this axis. Each mRNA distance was then normalized to this maximum distance, yielding values between 0 (nucleus) and 1 (cell periphery). Using this method, up to two different portions of cells were analyzed. Analysis was performed in blind.

#### mRNA tracking (and kymograph)

Time-lapse acquisitions of 2 min with a timeframe of 3 s were used to track mRNA along the axons. For each axon analyzed, the images were rotated so that the visible growth cone located on the right and the cell body on the left. In each axon, the analysis was performed in the same region, that is, in the 50 μm preceding the axon terminal. A proper threshold was applied to identify discrete mRNA dots. mRNA tracks were then generated using the tracking module system (Particle tracking) of the NIS elements AR software. Tracking parameters were set to obtain the best fitting and kept constant between different conditions and experiments (motion model=constant speed; centroid calculation=intensity_bright; maximum gap size between frames=3; standard deviation multiplication factor=3). Tracks with mRNAs detected for 5 or less frames were excluded from the analysis. Particle tracking analysis was then performed using a custom-made add-on MATLAB script. For each trajectory, the following parameters were defined: net displacement (ND, the difference in x-coordinates between the first and last points of the trace), lateral maximal displacement (LMD, the absolute value of the maximum difference in x-coordinates along the trace), and mean square displacement (MSD, calculated with the following equation: MSD(τ) ≤ [x(t + τ) − x(t)]2 + [y(t + τ) − y(t)] 2 >). ND and LMD were used to determine motion direction of each particle as follows: anterograde (ND > 5 μm) and retrograde (ND < −5 μm). Kymograph were created by drawing segmented lines on distal axon segments and using “kymograph by line” function in NIS elements AR software.

#### Mean pixel intensity

For the analysis, segments of isolated distal axons were selected excluding the growth cone area and analyzed using the best-focused individual image from a Z-stack. A segmented line 0.6 μm wide and 20–30 μm long was drawn along the axon segment based on the GFP signal to ensure unbiased analysis. Mean pixel intensity was then measured in the corresponding channel. Background signal mas measured in an axon-free area close to the analyzed segment and subtracted to the measured values.

#### MCP-MS2 system

In live-imaging experiments, *lc3b* mRNA was visualized through fluorescently tagged MCP binding to MS2 stem loops. RAB5-positive vesicles and *lc3b*-MS2 reporter puncta were scored as colocalizing when spatial overlap between the two fluorescent signals persisted for >10 s in time-lapse image sequences. Colocalization events were manually quantified.

### APEXseq and data analysis

Library preparation with ultra-low input RNA and 2 × 150-bp sequencing were performed on an Illumina HiSeq by GENEWIZ (Azenta Life Sciences). FASTQ sequencing reads were checked for their overall quality using FastQC v0.11.8. Raw reads were adaptor-trimmed and quality-filtered with Trimmomatic 0.39 (Bolger et al, 2014) before mapping to the hg38 (GRCh38) mouse reference genome (https://www.gencodegenes.org/human/) with STAR_2.5.3a (--outFilterMismatchNmax 10, Dobin and Gingeras, 2016). Gene counts were obtained using featureCounts v1.6.4 (Liao et al, 2014) and the GENCODE annotation release 31 (wgEncodeGencodeBasicV31). Data analysis have been performed using Bioconductor version 3.13 (BiocManager 1.30.16, R 4.1.1). Overall, 24,233 genes with more than 5 mean counts per condition were included in the analysis. Batch effects derived from the usage of different cell lines were moderated through the Combat-seq method (sva package 3.40.0, Zhang et al, 2020). To visually inspect the similarity between the samples in our dataset, we applied the DESeq2 (version 1.32.0, Love et al, 2014) variance stabilizing transformation (VST), performed Principal Component Analysis (PCA), and calculated the distance between each pair of samples. APEX-specific genes were assessed by differential gene expression (DGE) analysis using DESeq2, comparing samples originating from each APEX cell line (plus H₂O₂) against the ensemble of the pooled controls (minus H₂O₂). In each comparison, only the protein-coding genes (annotated using bioMart package 2.48.3 “hsapiens_gene_ensembl”, gene_biotype “protein_coding”) with at least 10 read counts and 1 FPKM in all samples of the same condition were tested, leading to the analysis of 9854, 9824, and 9531 genes for 2xFYVE-APEX, RAB5A-APEX, and NES-APEX, respectively. *P* value adjustment was performed using Benjamini–Hochberg (“BH”) false discovery rate (FDR) correction procedures. Gene ontology enrichment analyses were conducted through the clusterProfiler package 4.0.5 (Yu et al, 2012). Only non-redundant processes (function simplify of the package clusterProfiler, similarity cutoff used = 0.6) with *P*-adjusted lower than 0.1 were visualized. Plot and images were created using ggplot2 3.3.5.

### Statistical analyses

Parametric or non-parametric analyses were used after having tested Gaussian distribution of data with D’Agostino–Pearson omnibus normality test or Shapiro–Wilk normality test. To compare data distribution of two groups, the Kolmogorov–Smirnov test was used. The *n* number for each experiment and details of statistical analysis are described in the figure legends or main text. Statistical significance is defined as follows: n.s. (not significant), *P < 0.05, **P < 0.01, ***P < 0.001. Statistical analysis was performed using the GraphPad Prism software.

## Supplementary information

Peer Review File

Dataset EV1

Movie EV1

Source data Fig. 1

Source data Fig. 2

Source data Fig. 3

Source data Fig. 4

Source data Fig. 5

Source data Fig. 6

Source data Fig. 7

Expanded View Figures

## Data Availability

The genomic data of this publication have been deposited to the ArrayExpress database and assigned the following accession number: E-MTAB-15783. This is the link for having access: https://www.ebi.ac.uk/biostudies/ArrayExpress/studies/E-MTAB-15783?key=6a222aff-ab90-4af0-a7fd-800f8ea6d98b. The source data of this paper are collected in the following database record: biostudies:S-SCDT-10_1038-S44319-026-00867-5.
