# Supplementary material for: Local translation of endosome-associated lc3b mRNA in axons contributes to endosomal clearance
Source: EMBO Rep. 2026 Jul 17;27(18):5233–61. doi: 10.1038/s44319-026-00867-5 (PMC13601600; doi:10.1038/s44319-026-00867-5)
Supplement: Supplementary file 1 [file 44319_2026_867_MOESM11_ESM.pdf]

Expanded View Figures

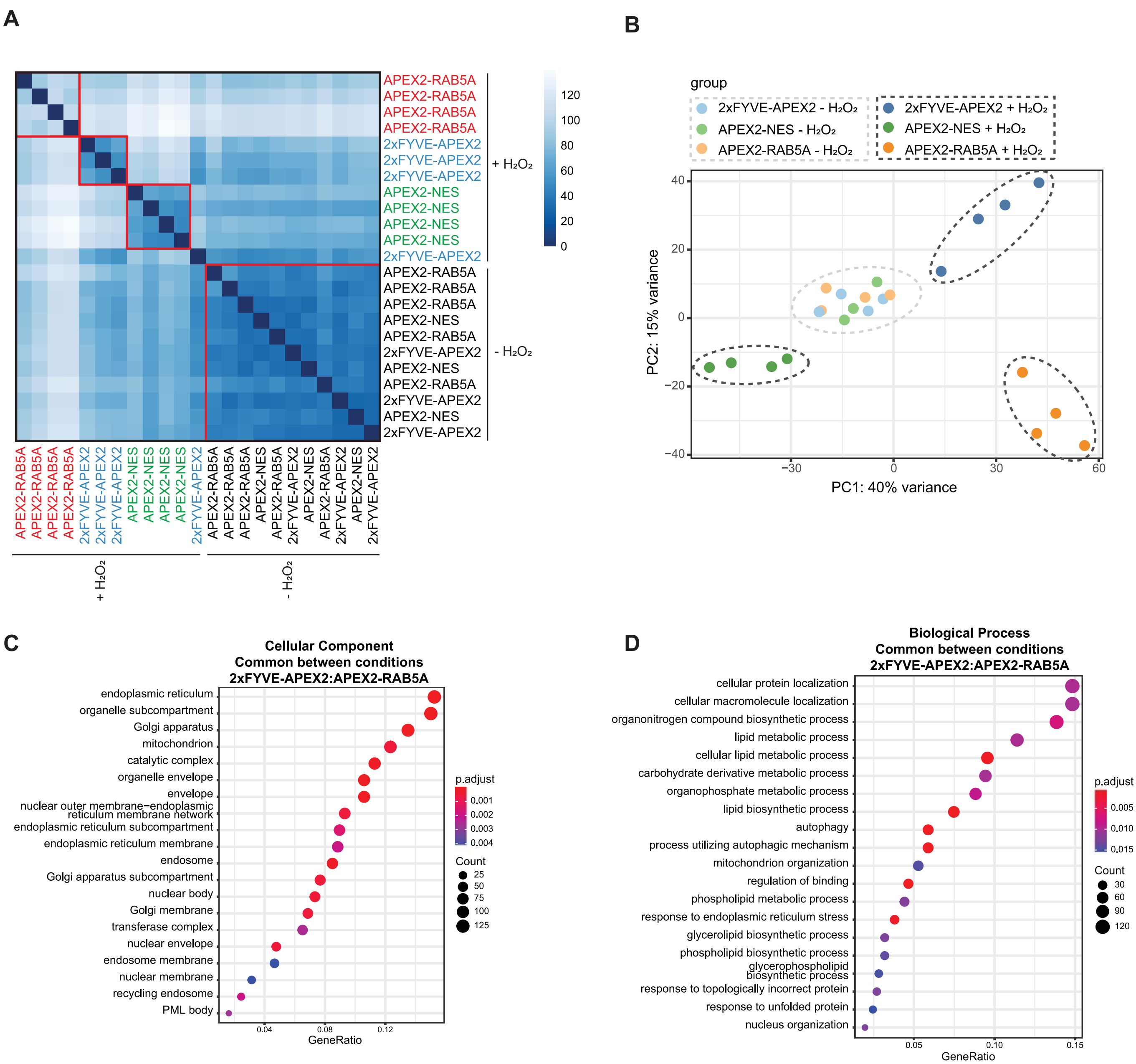

Figure EV1. APEXseq identifies distinct transcriptomes in H_2O_2-treated and untreated samples.

(**A**) Heatmap showing similarity and variance between RNA samples obtained from each APEX2 cell line treated with or without H_2O_2 in 4 independent biological replicates. Color scale indicates distances between samples. (**B**) Principal Component Analysis (PCA) showing the clustering of untreated samples from each APEX2 line together, and the separation between samples from different APEX2 cell lines treated with H_2O_2. (**C**) Gene Ontology Cellular Component analysis of mRNAs identified in both 2xFYVE-APEX2 and APEX2-RAB5A conditions, showing the top 20 enriched categories. (**D**) Gene Ontology Biological Process analysis of mRNAs identified in both 2xFYVE-APEX2 and APEX2-RAB5A conditions, showing the top 20 enriched categories. (**C**, **D**) Hypergeometric test.

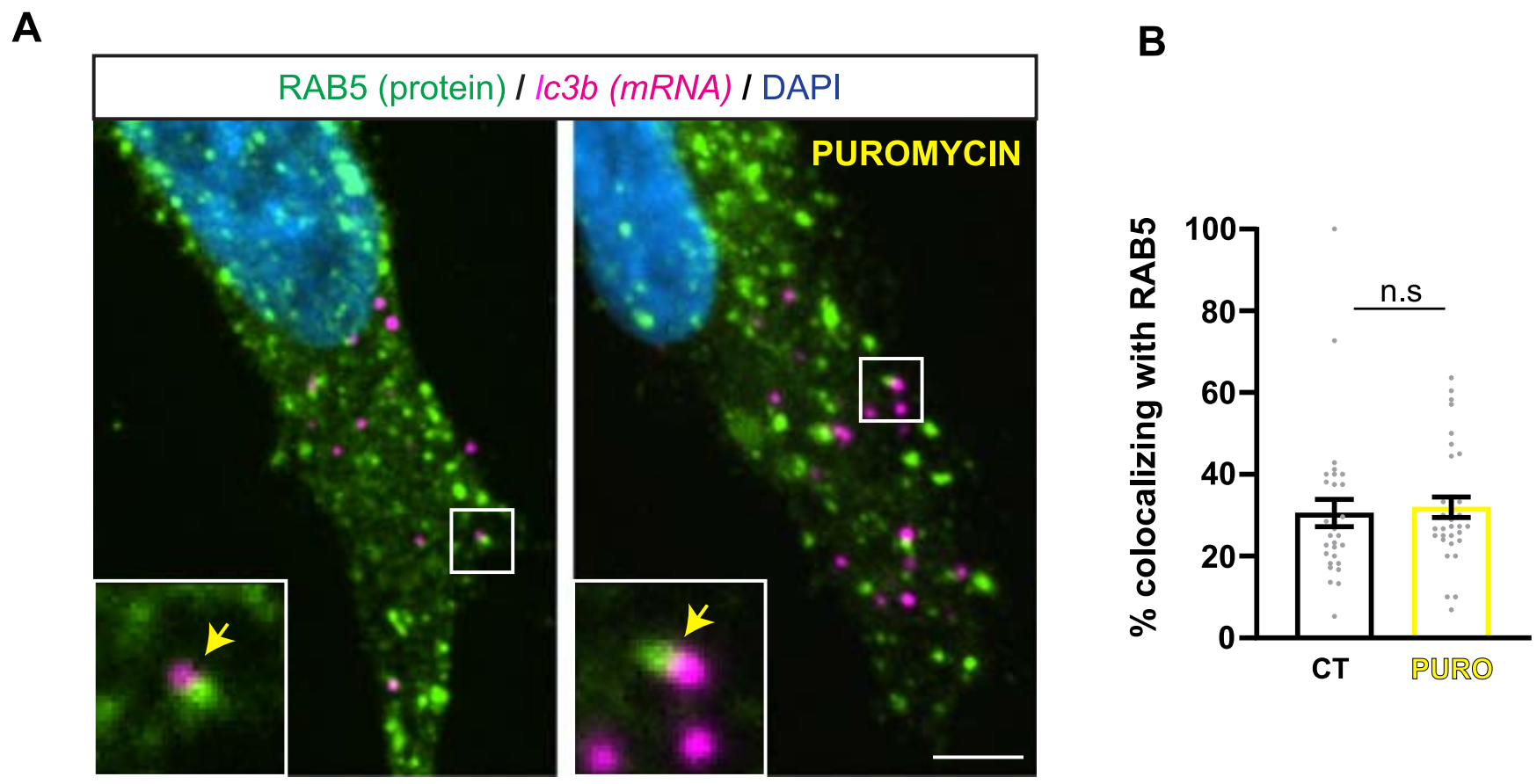

Figure EV2. ***lc3b* mRNA association with RAB5-positive endosomes is puromycin insensitive.**

(**A**) smFISH images of *lc3b* mRNA (in magenta) in SH-SY5Y cells treated with vehicle or 100 µM puromycin and stained against RAB5 (in green). Arrows indicate *lc3b* mRNA colocalizing with RAB5 signal. Scale bar: 3 µm. (**B**) Colocalization analysis. Dots represent cells (vehicle $n = 30$ cells, puromycin $n = 33$ cells, $P = 0.2804$, 3 exp). Mann–Whitney test. Mean ± SEM.

 © The Author(s)

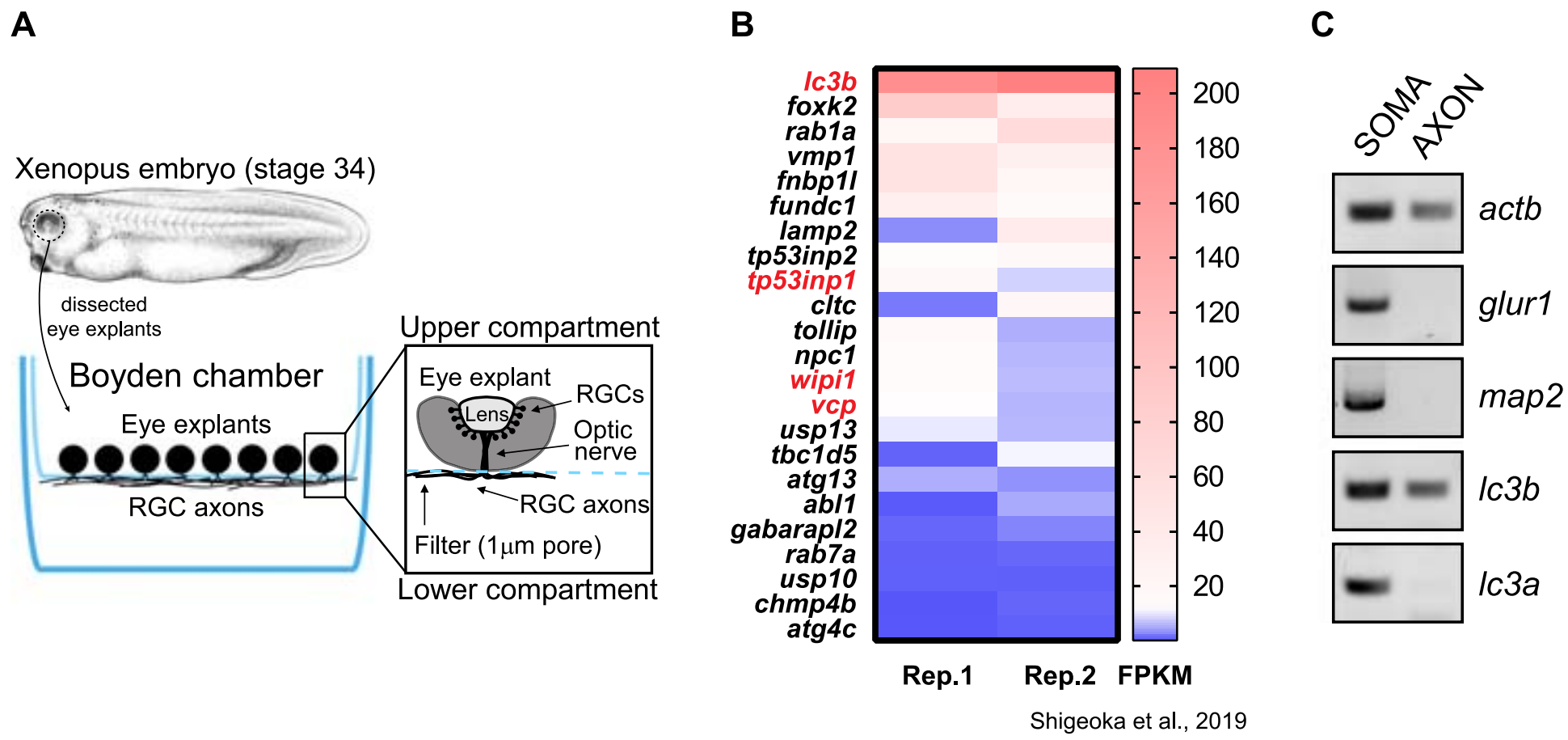

Figure EV3. Autophagy-related mRNAs are present in *Xenopus* RGC axons.

(**A**) Schematic representation of compartmentalized chambers (Boyden chambers) used to isolate somatodendritic and axonal compartments of *Xenopus* RGCs. (**B**) Heatmap showing the ranking of abundance (average FPKM) of autophagy-related mRNAs in *Xenopus* RGC axons (Shigeoka et al, 2019). Red indicates mRNA identified by APEXseq in Fig. 1. (**C**) RT-PCR analysis of cDNA synthesized from somatodendritic and axonal RNAs ($n = 3$ exp.). *actb*, *glur1* and *map2* mRNAs (on the left) show purity of axonal RNAs.

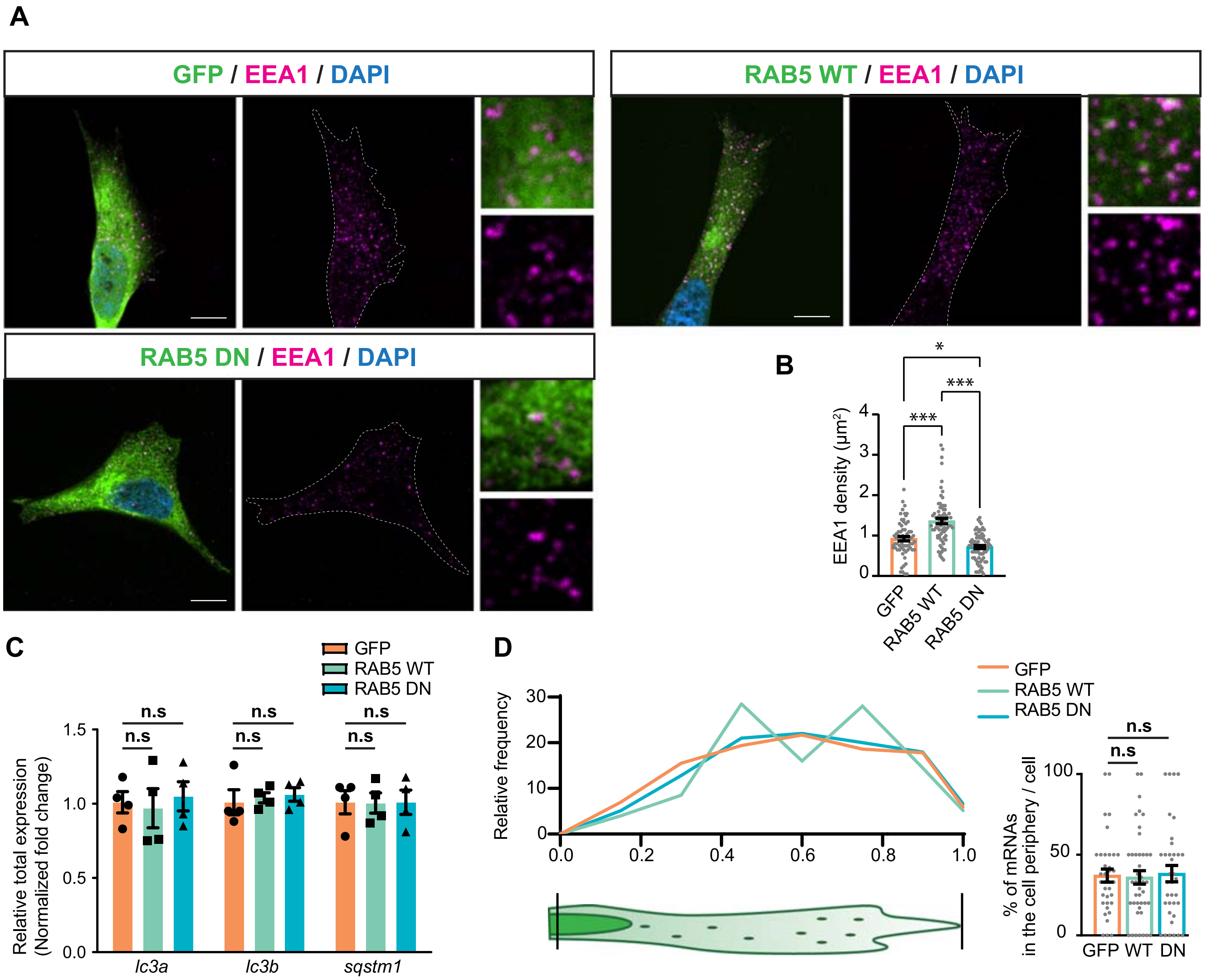

Figure EV4. Alteration of RAB5 activity does not impair *lc3b* mRNA expression or its distribution in SH-SY5Y cells.

(**A**) IF images of EEA1 signal (in magenta) in SH-SY5Y cells expressing the indicated constructs (in green). (**B**) Quantification of EEA1 signal density. Dots represent cells (GFP: $n = 64$ cells, RAB5WT: $n = 74$ cells; RAB5DN: 76 cells, $n = 3$ exp). (**C**) RT-qPCR analysis in SH-SY5Y cells expressing the indicated constructs ($n = 4$ exp). (**D**) *lc3b* mRNA distribution in SH-SY5Y cells expressing the indicated constructs. Quantification of the relative frequency distribution of mRNA distances from the nucleus relative to the maximum cell extension. Dots represent cells (GFP: $n = 36$ cells, RAB5WT: $n = 43$ cells; RAB5DN: 36 cells, $n = 3$ exp). Mean ± SEM. (**B–D**) Kruskal–Wallis test. Scale bar: (**A**) 10 μm.

 © The Author(s)

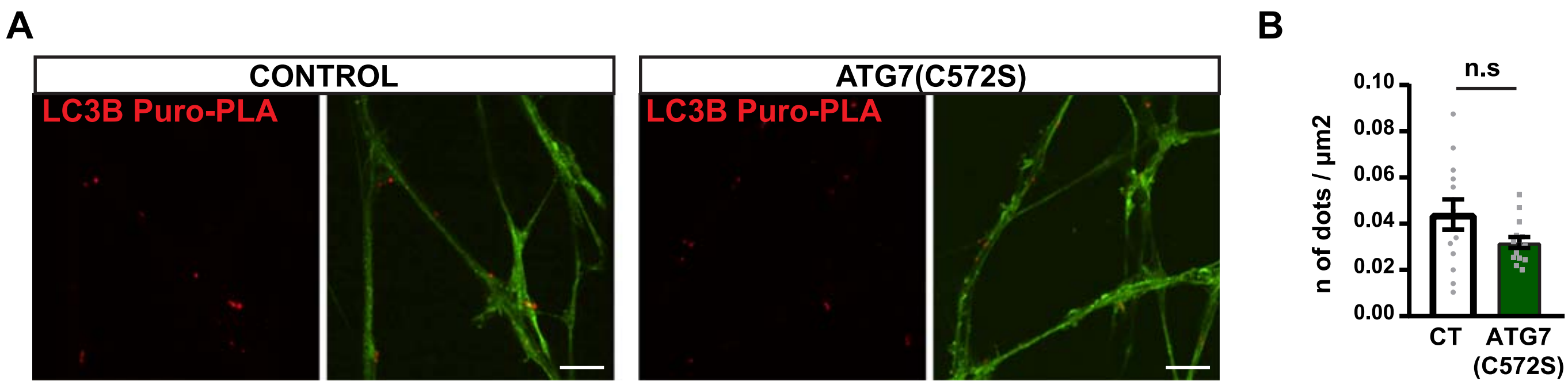

Figure EV5. Expression of the ATG7(C572S) mutant does not affect LC3B synthesis in axons.

(**A**) Images showing LC3B Puro-PLA signal in axons. Scale bar: 10 µm. (**B**) Quantification in the indicated conditions (CT, $n = 13$ fields of view; ATG7(C572S), $n = 15$ fields of view; $n \geq 6$ explants, 3 exp). Mean ± SEM. (**B**) Unpaired *t* test.

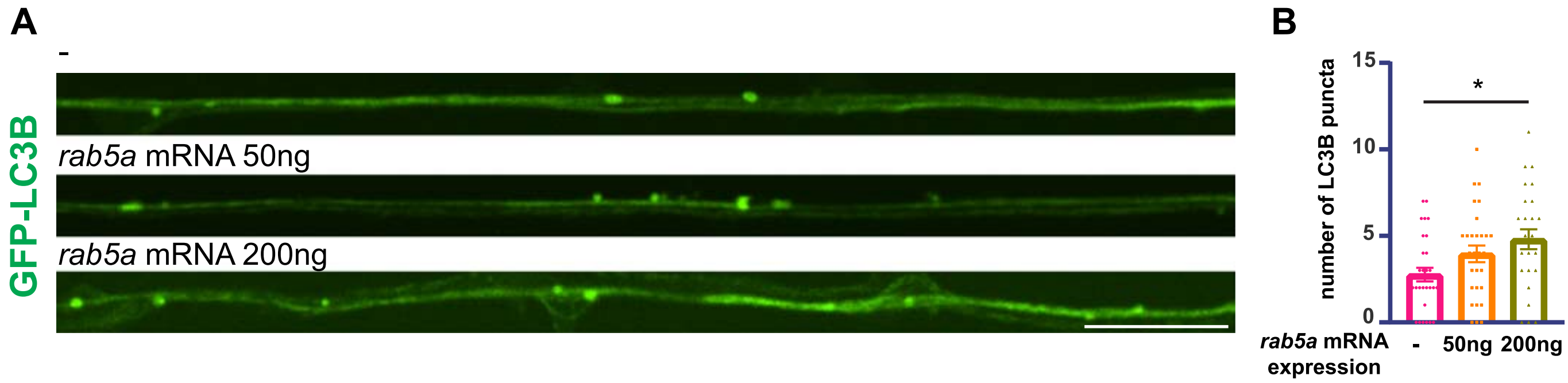

Figure EV6. RAB5 overactivation drives an increase in GFP-LC3B puncta in axons.

(**A**) Images showing GFP-LC3B signal in axons expressing increased concentration of *rab5a* mRNA. (**B**) Quantification of GFP-LC3B puncta. Dots represent axons (CT: $n = 30$; RAB5 50 ng: $n = 29$; RAB5 200 ng: $n = 26$, $^*P = 0.0107$, $n \geq 4$ explants, 2 exp). Kruskal–Wallis test. Mean ± SEM. Scale bar: (**A**) 10 µm.

 © The Author(s)

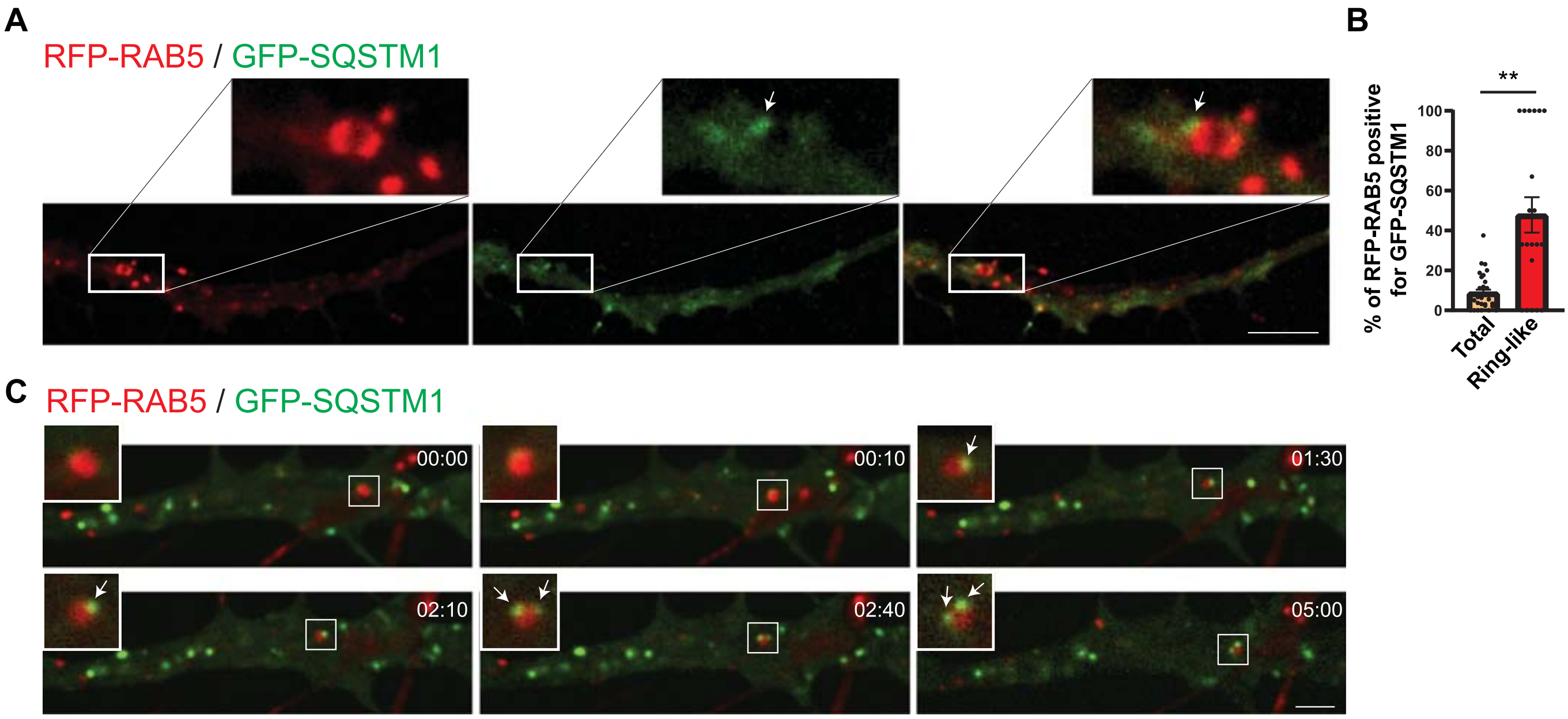

Figure EV7. RAB5 overexpression induces enlarged endosomes positive for SQSTM1.

(**A**) Image showing GFP-SQSTM1 signal colocalizing with ring-like RFP-RAB5 positive endosome in axons. Arrow indicates GFP-SQSTM1 signal on RFP-RAB5-endosome. (**B**) Colocalization between RFP-RAB5 and GFP-SQSTM1. Dots represent axons ($n = 31$, $n \geq 6$ explants, $P = 0.0011$, 3 exp). Mann–Whitney test. Mean ± SEM. (**C**) Time-lapse sequence on axons expressing RFP-RAB5 and GFP-SQSTM1. Arrows indicate a RFP-RAB5-endosome becoming GFP-SQSTM1 positive. Time is indicated as min:sec. Scale bar: (**A**) 5 μm, and (**C**) 2 μm.

© The Author(s)
