# Supplementary material for: Local translation of endosome-associated lc3b mRNA in axons contributes to endosomal clearance
Source: EMBO Rep. 2026 Jul 17;27(18):5233–61. doi: 10.1038/s44319-026-00867-5 (PMC13601600; doi:10.1038/s44319-026-00867-5)
Supplement: Supplementary file 2 [file 44319_2026_867_MOESM1_ESM.pdf]

EMBO reports

Local translation of endosome-associated lc3b mRNA in axons contributes to endosomal clearance

Jean-Michel Cioni, Francesca Pappacena, Cinzia Caterino, Stefano de Pretis, Cristina Sironi, Michela Bruni, Alessandro Esposito, and Giuliana Cesare

Corresponding author(s): Jean-Michel Cioni (cioni.jeanmichel@hsr.it)

Review Timeline:

Submission Date:	15th Oct 25
Editorial Decision:	21st Oct 25
Appeal Received:	18th Nov 25
Editorial Decision:	17th Dec 25
Revision Received:	12th May 26
Editorial Decision:	12th Jun 26
Revision Received:	20th Jun 26
Accepted:	26th Jun 26

Editor: Martina Rembold

Transaction Report:

(Note: With the exception of the correction of typographical or spelling errors that could be a source of ambiguity, letters and reports are not edited. Depending on transfer agreements, referee reports obtained elsewhere may or may not be included in this compilation. Referee reports are anonymous unless the Referee chooses to sign their reports.)

1st Editorial Decision **21st Oct 2025**

Dear Dr. Cioni,

Thank you for the submission of your manuscript to EMBO reports. I apologize for the delay in handling your manuscript, but I have now read and discussed your work with my colleagues here, and I regret to say that we all agree that it is not well suited for our journal.

We appreciate that your study reports that mRNAs encoding autophagy-related genes, such as lc3b and sqstm1, localize to endosomes in neuroblastoma cells and Xenopus RGCs. You find that LC3B is locally translated on endosomes. Your data further suggest that LC3B lipidation is required for endosomal homeostasis

We acknowledge that your findings indicate that autophagy-related proteins are locally translated at endosomes, which is an interesting observation. We however also note that the manuscript as it stands, does not provide further evidence that locally translated and lipidated LC3B mediates endosomal quality control, given that ATG7 inhibition would globally affect LC3B and autophagy within the cell. Taking this consideration into account, we overall feel that the manuscript as it stands does not represent the kind of advance we are looking for in an EMBO reports paper. We would need more evidence that the endosomal translation of LC3B has functional significance. We have there decided not to proceed with in-depth peer review.

That said, your work is an excellent candidate for our partner journal Life Science Alliance (http://www.life-science-alliance.org/; our broad scope Open Access journal published in partnership between the EMBO-, Rockefeller University-, and Cold Spring Harbor Laboratory Presses). The editors of Life Science Alliance would be pleased to send your manuscript for in-depth peer review; no reformatting is required.

We very much hope you will be interested in this option: please follow the link below for transfer. Tim Fessenden, Executive Editor of Life Science Alliance (t.fessenden@life-science-alliance.org), will be pleased to answer any questions.

Yours sincerely,

Martina Rembold, PhD
Senior Editor
EMBO reports

** As a service to authors, EMBO Press provides authors with the ability to transfer a manuscript that one journal cannot offer to publish to another journal, without the author having to upload the manuscript data again. To transfer your manuscript to another EMBO Press journal using this service, please click on
Link Not Available

Jean-Michel Cioni, Ph.D.,
Head of RNA Biology of the Neuron laboratory
IRCCS San Raffaele Scientific Institute, Division of Neuroscience
Via Olgettina, 58 - 20132 Milano
Tel: 02 26439481 / 02 26434110
Email: cioni.jeanmichel@hsr.it

Milano, November 17th, 2025

Dear Editor,

We are pleased to submit a revised version of our manuscript, entitled "**Endosome-associated lc3b mRNA is locally translated in axons, where the protein contributes to endosomal clearance**" for consideration in EMBO Reports. In accordance with your guidance, we performed additional experiments to further extend and reinforce our findings. Notably, these new data strengthen our conclusion that locally damaged endosomes in axons can be targeted by LC3B, providing increased support for the model we propose. We believe that the expanded dataset enhances the rigor and impact of the study, and we are delighted to present this updated version for your evaluation.

RNA localization is an evolutionarily conserved process that plays vital roles in large and compartmentalized cells like neurons. In such cells, tightly regulated protein synthesis, both in time and space, is necessary to ensure correct neuronal development, function, and long-term maintenance. Disruption of this mechanism is increasingly linked to neurodevelopmental and neurodegenerative diseases, highlighting the need to understand how mRNA localization regulates neuronal function.

Multiple organelles are emerging as key hubs for regulating mRNA trafficking and subcellular translation across various cell types, including neurons. Recent studies, including our own, have identified ribosomes and mRNAs closely associated with endosomes in both axons and dendrites. Additionally, lysosomes have been shown to transport specific mRNAs and support local protein synthesis essential for mitochondrial function and axonal integrity. Together, these findings point to an increasingly recognized direct link between the endo-lysosomal system and mRNA localization and translation. **Despite this progress, a comprehensive knowledge of the specific transcripts that can associate with these organelles, and the functional significance of these interactions, remains to be achieved.**

In this study, we investigated the transcriptome associated with endosomes by combining proximity labeling with RNA sequencing (APEX-seq). This approach uncovered a vast set of mRNAs localized to endosomes,

I.R.C.C.S. Ospedale San Raffaele
Gruppo San Donato

including several linked to autophagy. We validated the association of *lc3b* mRNA with Rab5-positive endosomes in both cultured cells and *Xenopus* axons. Furthermore, we obtained evidence supporting that Rab5-endosomes can display LC3B produced via local protein synthesis in axons. This led us to identify a role for LC3B in regulating endosomal number and size within axons, including during the clearance of damaged endosomes caused by Rab5 overactivation, a process implicated in neurodegenerative pathways such as Alzheimer's disease, or following Chloroquine application.

In summary, this manuscript presents a **characterization of the transcripts associated with endosomal compartments** in a neuronal cell line, laying the foundation for future investigations. By revealing **why a specific transcript, *lc3b* mRNA, associates with these organelles**, our findings uncover new fundamental aspects of neuronal cell biology. Moreover, our demonstration of **LC3B's role in regulating endosomal clearance** in axons offers valuable insights into how axons maintain organelle turnover under both physiological and pathological conditions.

We therefore believe that our study will be of broad interest to the scientific community and we hope that *EMBO reports* will give the manuscript serious consideration.

We look forward to hearing from you.

Yours sincerely,

Jean Michel Cioni, PhD
Head of RNA Biology of the Neuron laboratory
IRCCS Ospedale San Raffaele
Via Olgettina, 58 - 20132 Milano
Tel: 02 26439481 / 02 26434110
Email: cioni.jeanmichel@hsr.it

Appeal - Editorial Decision **17th Dec 2025**

Dear Dr. Cioni

Thank you for the submission of your manuscript to EMBO reports. We have now received the full set of referee reports that is pasted below.

As you will see, the referees acknowledge that the findings are potentially interesting and consider the data overall solid, but they also have a number of suggestions to strengthen the manuscript, that should be addressed.

Given the constructive and supportive comments, I would like to invite you to revise your manuscript with the understanding that the referee concerns must be fully addressed and their suggestions taken on board. Please address all referee concerns in a complete point-by-point response. Acceptance of the manuscript will depend on a positive outcome of a second round of review. It is EMBO reports policy to allow a single round of major revision only and acceptance or rejection of the manuscript will therefore depend on the completeness of your responses included in the next, final version of the manuscript.

We realize that it is difficult to revise to a specific deadline. In the interest of protecting the conceptual advance provided by the work, we recommend a revision within 3 months (19th Mar 2026). Please discuss the revision progress ahead of this time with the editor if you require more time to complete the revisions.

IMPORTANT NOTE: we perform an initial quality control of all revised manuscripts before re-review. Your manuscript will FAIL this control and the handling will be DELAYED if the following APPLIES:
1) A data availability section providing access to data deposited in public databases is missing. If you have not deposited any data, please add a sentence to the data availability section that explains that.
2) Your manuscript contains statistics and error bars based on n=2. Please use scatter blots in these cases. No statistics should be calculated if n=2.

When submitting your revised manuscript, please review the submission guidelines (https://link.springer.com/journal/44319/submission-guidelines#cms-Revised-submissions) and carefully review the instructions that follow below. Failure to include requested items will delay the evaluation of your revision.

1) a .docx formatted version of the manuscript text (including legends for main figures, EV figures and tables). Please make sure that the changes are highlighted to be clearly visible.

2) individual production quality figure files as .eps, .tif, .jpg (one file per figure). See hhttps://media.springernature.com/original/springer-cms/rest/v1/content/27825798/data/v1 for more info on how to prepare your figures.

3) We replaced Supplementary Information with Expanded View (EV) Figures and Tables that are collapsible/expandable online. A maximum of 5 EV Figures can be typeset. EV Figures should be cited as 'Figure EV1, Figure EV2" etc... in the text and their respective legends should be included in the main text after the legends of regular figures.

- For the figures that you do NOT wish to display as Expanded View figures, they should be bundled together with their legends in a single PDF file called *Appendix*, which should start with a short Table of Content. Appendix figures should be referred to in the main text as: "Appendix Figure S1, Appendix Figure S2" etc. See 'Expanded View' section of the submission guidelines.

- Additional Tables/Datasets should be labeled and referred to as Table EV1, Dataset EV1, etc. Legends have to be provided in a separate tab in case of .xls files. Alternatively, the legend can be supplied as a separate text file (README) and zipped together with the Table/Dataset file.

4) a .docx formatted letter INCLUDING the reviewers' reports and your detailed point-by-point responses to their comments. As part of the EMBO Press transparent editorial process, the point-by-point response is part of the Review Process File (RPF), which will be published alongside your paper.

5) a complete author checklist, which you can download from our author guidelines. Please insert information in the checklist that is also reflected in the manuscript. The completed author checklist will also be part of the RPF.

6) Please note that all corresponding authors are required to supply an ORCID ID for their name upon submission of a revised manuscript (), which can be linked to your profile in the manuscript submission system.

7) Before submitting your revision, primary datasets produced in this study need to be deposited in an appropriate public database. Please remember to provide a reviewer password if the datasets are not yet public. The accession numbers and database should be listed in a formal "Data Availability" section placed after Methods. Please note that the Data Availability

Section is restricted to new primary data that are part of this study. * Note - All links should resolve to a page where the data can be accessed. *

If your study has not produced novel datasets, please mention this fact in the Data Availability Section.

8) At EMBO Press we ask authors to provide source data for the main manuscript figures. You will receive a separate email with instructions for providing source data with your revised manuscript, including how to upload and organize the files.

9) Our journal also encourages inclusion of *data citations in the reference list* to directly cite datasets that were re-used and obtained from public databases. Data citations in the article text are distinct from normal bibliographical citations and should directly link to the database records from which the data can be accessed. In the main text, data citations are formatted as follows: "Data ref: Smith et al, 2001" or "Data ref: NCBI Sequence Read Archive PRJNA342805, 2017". In the Reference list, data citations must be labeled with "[DATASET]". A data reference must provide the database name, accession number/identifiers and a resolvable link to the landing page from which the data can be accessed at the end of the reference.

10) Regarding data quantification, the following points must be specified in each figure legend:

- the name of the statistical test used to generate error bars and P values,

- the number (n) of independent experiments (please specify technical or biological replicates) underlying each data point,

- the nature of the bars and error bars (s.d., s.e.m.),

- If the data are obtained from n Program fragment delivered error ``Can't locate object method "less" via package "than" (perhaps you forgot to load "than"?) at //ejpvfs23/sites23b/embor_www/letters/embor_decision_revise_and_review_sr_any.txt line 55.' 2, use scatter blots showing the individual data points.

Discussion of statistical methodology can be reported in the materials and methods section, but figure legends should contain a basic description of n, P and the test applied.

- Please also include scale bars in all microscopy images.

11) The journal requires a statement specifying whether or not authors have competing interests (defined as all potential or actual interests that could be perceived to influence the presentation or interpretation of an article). In case of competing interests, this must be specified in your disclosure statement.

12) All Materials and Methods need to be described in the main text using our 'Structured Methods' format, which is required for all research articles. According to this format, the Methods section includes a Reagents and Tools Table (listing key reagents, experimental models, software and relevant equipment and including their sources and relevant identifiers) followed by a Methods and Protocols section describing the methods using a step-by-step protocol format. The aim is to facilitate adoption of the methodologies across labs. More information on how to adhere to this format as well as a downloadable template (.docx) for the Reagents and Tools Table can be found in our author guidelines.

We would also welcome the submission of cover suggestions, or motifs to be used by our Graphics Illustrator in designing a cover.

As part of the EMBO publication's Transparent Editorial Process, EMBO reports publishes online a Review Process File (RPF) to accompany accepted manuscripts. This File will be published in conjunction with your paper and will include the referee reports, your point-by-point response and all pertinent correspondence relating to the manuscript.

You are able to opt out of this by letting the editorial office know (emboreports@embo.org). If you do opt out, the Review Process File link will point to the following statement: "No Review Process File is available with this article, as the authors have chosen not to make the review process public in this case."

I look forward to seeing a revised form of your manuscript when it is ready.

Kind regards,

Martina Rembold, PhD
Senior Editor
EMBO reports

=================

Referee #1:

Summary
The manuscript addresses the interesting question how localization and function of LC3b mRNA in axons is connected to Rab5-positive endosomes. The study is presents interesting and relevant observations and is conducted well. However, several key findings require some further clarification or additional controls by the authors, particularly experiments concerning lc3b RNA localization, the wildtype Rab5 and Rab5DN mutant overexpression, as well as potential confounding effects of chloroquine on lysosomes/autophagy.
Additionally, some figures and quantitative analyses need to be improved for consistency and clearer presentation, and several sections in the text would benefit from further explanation.
Overall the manuscript is very promising, but addressing the stated points will strengthen the conclusions and improve clarity.

Major points
• Is the association between Rab5-positive endosomes and lc3b and sqstm1 RNA translation dependent, so driven by ribosome association to Rab5-positive endosomes, similarly to the FERRY complex? Is the FERRY complex responsible for the association of lc3b RNA?
• Does Rab5DN expression alter endosomal distribution as the Rapamycin experiment does? Although Rab5DN presumably interferes with endogenous Rab5 function, endogenous Rab5 protein is still present and may complicate the interpretation of lc3b mRNA localization, which may not rely on its GTPase function.
• Also, does overexpression of Rab5wt alter the cellular distribution pattern of lc3b mRNA?
• To confirm findings of TEM, it would be nice to have an IF staining of autophagosomes compared to the abundance of LC3B. If LC3B is really localized to structures different from APs, this should also show up in IF analysis.
• The idea that locally translated lc3b directly decorates damaged endosomes is very intriguing. Could this approach also be used by other organelles? Is there an colocalization of lc3b mRNA/translation to mitochondria or lysosomes, potentially even after treatment with compounds that damage these organelles (CCCP, Choloroquine!?) Both possibilities are interesting, either this being an endosome specific pathway or a generalizable concept to enable autophagy in axons.
• Overexpressing ATG7 (C572S) blocks ATG12-ATG5 conjugation and thus autophagy in general. Similarly, chloroquine (CHQ) inhibits autophagy as it prevents fusion of autophagosomes with lysosomes. It is therefore not surprising to find more/damaged Rab5-endosomes in axons in both conditions. Yet what happens to lc3b mRNA and its local translation? Is the mRNA still present and does translation still occur in ATG7 mutant cells, but fails to target endolysosomes?

Minor points
• Please maintain consistency in how you position the labels in the figure legends, ensuring they are placed uniformly either before or after the corresponding description.
• The representative colors for the immunofluorescence are very confusing. Single channel images should stay in one color (either white, green, or red), 2-channel images should also stay in 2 colors (e.g. magenta-green to make this manuscript also accessible for red-green color blindness). Especially within a figure, the two channels for colors should remain the same (e.g. figure 2A).
• Line 129-130: The authors write "streptavidin-based immunoprecipititation". Since streptavidin is a protein not an antibody, it is rather only a precipitation or pulldown.
• Line 167-169: Please indicate a short statement, why eea1 was used (as the authors did for lc3b RNA).
• Fig. 2A should be separated in 2 visually separate panels to emphasize the distinct data:
o Fig 2A: colocalization between eea1, lc3, sqstm1 RNA and Rab5.
o Fig 2B: colocalization between lc3 and eea1 and Rab7 (early vs. late endosomes)
• Fig. 2F: please indicate what CT abbreviates
• Figure 2F: lc3 and sqstm1 should have same number of experiments and comparable numbers of cells. Please add a 3rd replicate to lc3 localization.
• Figure 2G,H: please have equal no. of experiments
• In all plots it should be indicated whether results are non-significant or if there has not been a statistical analysis. Please add the significance bars for figures 2F,H,I,L; 3B,C,D,G; 4B; 5E; EV3A,B; EV4.
• Please include a reference image for fig. 2L, where its visible where RAB5 localizes with LC3 mRNA
• The kymograph of figure 3A looks like its pieced together (left side). The authors should make sure it is one graph with the same settings.
• Fig. 3C: please indicate what A.R. means. (Potentially, the graph should be rearranged so that all Rs are grouped together and compared and all As are.)
• Please indicate in a short statement what scfvGFP is used for.
• Fig 3E: Please include a reference picture of a harringtonine treated axon.
• Fig 3F: Please include the lc3b Suntag signal in the image. Also, please include what the arrows indicate.
• Line 272: "endosomes could be locally targeted by LC3B in axons". The context of this statement is missing. Targeted by LC3B for what? Please elaborate.
• Fig 4A: a zoom in would be helpful to assess colocalization better

• Fig 4B: what does M1 and M2 refer to? Please indicate.
• Line 286-287: "autophagy is not limited to ER and mitochondria in developing axons". Please elaborate this statement or add a reference who claimed that autophagy in developing axons was limited in that way.
• Line 298: The text discusses ATG12-ATG5 conjugation, whereas Figure 4D depicts LC3B lipidation. Please adjust the wording to ensure consistency between the text and the figure content.
• It is not clear what data was used for fig 4E. If the same images were used as for fig. 4F, fig. 4E should be part of fig. 4F.
• Fig 5B, C: in the manuscript it is BSA-647 in the figure it is BSA-657. Please correct.
• Fig 5H: Colocalization is hard to detect with the colors magenta, green, red. I would suggest cyan, magenta, yellow or other more distinct colors.
• Fig 6D: since CHQ affects autophagy, LC3-GFP should also be shown in this panel.
• Line 420: please elaborate: "Local regulation" of/for what?
• Fig. EV1 A: x-axis labelling would be helpful
• Fig. EV1 B: -H2O2 samples should have a similar color pattern (e.g. cold colors) and the +H2O2 should have also a similar one (e.g. warm colors) to help distinguishing the samples
• Fig. EV2A: It would be helpful to mention in the text that figure EV2 depicts the schematic of the Boyden chamber
• Fig EV4: attached is a quantification that includes quantification of colocalization of ring-like Rab5 and sqstm1. Therefore, reference pictures of ring-like rab5 colocalizing with sqstm1 should be included in the image as well.

Referee #2:

In this manuscript the authors study the association of mRNAs with early endosomes in axons. Using proximity labeling in a cell culture model system they were able to identify numerous mRNAs that are associated with endosomes. Choosing lc3b mRNA as an example, they reveal a novel mechanistic link to autophagy. The author provide evidence showing that transport of lc3b mRNA and endosomal localization are two distinct processes, suggesting that mRNAs are transported towards early endosomes. Studying translation in vivo revealed local protein synthesis on the surface of endosomes functions most likely to target the translation product to the surface of endosomes. Since LCB3 protein is involved in autophagy, the authors hypothesis that LCB3 might be important for local degradation of endosomes by autophagy. To support this hypothesis the authors demonstrate that inhibition of LC3B by preventing its lipidation results in enlarged endosomes in developing axons. Furthermore, overexpression of the endosomal GTPase RAB5 resulted in larger endosomes and interestingly, lcb3 mRNA and protein accumulate at growing endosomes. Hence, local translation might be important to target dysfunctional endosomes. Finally, they demonstrate that stress treatment using chloroquine alters endosomal homeostasis and this correlates with the accumulation of LC3B most likely by local translation.
In summary, the authors combine a number of sophisticated in vivo approaches such as proximity labeling, life cell imaging of mRNA localization and translation as well as EM imaging. They compare cell culture models with growing vertebrate axons to demonstrate that the autophagy component LCB3 localize to stressed endosomes. They provide compelling evidence for a new mechanistic link of endosome-coupled translation and quality control by autophagy. Thus, the study merits publication in EMBO reports. However, prior to publication I would like to suggest some improvements.

Major points:
1. Since LC3B and autophagy is a main focus of the manuscript, it would be very helpful to include the state-of-the art on LC3B in the introduction of the manuscript.
2. I had difficulties to understand the rational that mRNA co-localization was verified by rapalog experiment targeting motile endosomes, whereas later it is shown that mRNA motility is independent of endosomes. Please, explain the rational of these experimental series in more detail.

Minor points:
1. For consistence, I would recommend to call the constructs: APEX2-RAB5A, APEX2-NES and APEX2-FYVE2 instead of 2xFYVE-APEX2.
2. Line 133: "A differential expression analysis..". The term "differential expression" is misleading, because the expression did not change.
3. Line 146: For the GO analysis the overlap of APEX2-RaB5A and 2xFYVE-APEX2 is most important. Focus on the 898 mRNAs that were found in both datasets.
4. Line 158: It is unclear how the "stress response mechanism" was identified.
5. Line 169: well known autophagy regulator LC3B and SQSTM1 are used but the proteins are not introduced. More details and citations are missing (see Major point 1).
6. Line 226: lc3a is mentioned but not introduced.
7. Line 320: Introduce BSA-647 as labeling tool.
8. Line 411: please compare the results on the coding region to the study by the Pelham lab
9. Discussion: provide an overview on the endosome-associated translation: which examples are known and what are the various functions.
10. A graphical model would be helpful
Final points: Could the authors comment on the aspect that the H2O2 treatment during APEX2 labeling might trigger a stress

response in the cells and therefore stressed endosomes are also part of the analysis. This would nicely explain the detection of LCB3 mRNA.

Referee #3:

The manuscript of Pappacena et al. presents a very intriguing research study, which identifies the pool of RNAs localized at endosomes and further shows that the mRNA lc3b is translated within neurons in SH-SY5Y neuronal cell culture and the RGCs of Xenopus. They took an APEX-seq approach to biotinylate RNAs close to the endosome using the FYVE domain and Rab5 as markers. They identified several RNAs involved in endocytosis, endosomal trafficking, neuronal processes, synaptic environment, and autophagy and stress response pathways. Authors then identified lc3b was locally being translated at endosomes, which may be needed for endosomal autophagy. Endosomal stressors such as Rab5 overexpression and chloroquine lead to previously described large endosomal rings, which the authors have found actively translate LC3B until structures are encased. This is a very exciting work that identifies a novel non-conventional autophagic pathway that maintains endosomal integrity through local translation. I would support the publication of this manuscript after the following minor concerns are addressed.

In Figure 5 B, C, the authors show that LC3b-positive vesicles partially colocalize with endocytosed BSA647 in the steady states, and the colocalization increased upon Rab5 overexpression. Together with ATG7 dominant mutants' data (Figure 4), the authors interpret these results as endocytic vesicles being constitutively targeted for autophagy through de novo LC3 lipidation on the Rab5-positive vesicle's membrane. However, an alternative interpretation could be, the synthesized LC3 protein incorporates into the nascent autophagosome, followed by fusion with Rab5-positive vesicles. Similar to the atypical autophagy pathway LC3-endocytic-associated-pathway (LEAP), which shows a similar phenotype of LC3 colocalization with Rab5 endosomes (Coelho et al 2022 Nat Comm).

In Figure 2J, K, L, the authors show that the deletion of the 3'-UTR leads to a decrease in the number of lc3b mRNA puncta in the distal axon, but that the association with Rab5 endosomes remains unchanged. Does the number of Rab5 endosomes change in the distal axon? A quantification of this data would also be helpful.

Minor suggestions:
Figure 2K-L: Please provide the corresponding images for this quantification.

Figure 3E - I would suggest adding the analysis of scfvGFP and including an image of harringtonine treatment to create a more complete figure.

Figure 3G - An axis label would be beneficial for this graph.

Figure 4B - Indicate what M1 and M2 (i.e., M1 is LC3B) either on the graph or in the legend.

Figure 4C - It would be beneficial to have labeling of structures within the TEM images to go with the text descriptions.

Figure 5A - Corresponding images for this graph would be a good addition to the supplement.

Figure 5B - The dynamics of the LC3B and BSA647 endosomes are noted in the kymograph, but no description or graph of the results is provided.

Figure 5G - Please provide the video that the stills are from to the supplement. Additionally, add stills between 1 - 10 minutes of the autophagic structure developing; it is not clear that there is an appearance of the ring structure after a single puncta. It would be helpful to see the RFP channel by itself for these stills as well to show that Rab5 is being incorporated into these autophagic compartments.

Referee #1:

Summary
The manuscript addresses the interesting question how localization and function of LC3b mRNA in axons is connected to Rab5-positive endosomes. The study is presents interesting and relevant observations and is conducted well. However, several key findings require some further clarification or additional controls by the authors, particularly experiments concerning lc3b RNA localization, the wildtype Rab5 and Rab5DN mutant overexpression, as well as potential confounding effects of chloroquine on lysosomes/autophagy.
Additionally, some figures and quantitative analyses need to be improved for consistency and clearer presentation, and several sections in the text would benefit from further explanation.
Overall the manuscript is very promising, but addressing the stated points will strengthen the conclusions and improve clarity.

We sincerely thank the reviewer for the positive feedback and the very helpful suggestions. In response, we carried out additional experiments and revised the manuscript to improve the clarity of both the text and the figures.

Major points

• Is the association between Rab5-positive endosomes and lc3b and sqstm1 RNA translation dependent, so driven by ribosome association to Rab5-positive endosomes, similarly to the FERRY complex? Is the FERRY complex responsible for the association of lc3b RNA?

This is a very interesting point. To assess the dependency on translation, we performed additional smFISH experiments and analyzed the association of *lc3b* mRNA with RAB5 following puromycin treatment. Puromycin is a protein synthesis inhibitor that disrupts ribosome-mRNA interactions and is commonly used to test whether mRNA localization depends on ribosome association.

Our results revealed no significant change in the percentage of *lc3b* mRNA colocalizing with the RAB5 immunofluorescent signal (Figure EV2). These findings suggest that endosomal association is not mediated by the nascent peptide.

As suggested, we also investigated whether *lc3b* or *sqstm1* mRNAs have been reported to interact with the FERRY complex (Schuhmacher et al.) and found no evidence supporting such associations. It will be important in future studies to identify the RNA-binding protein(s) responsible for targeting *lc3b* mRNA to organelle membranes.

• Does Rab5DN expression alter endosomal distribution as the Rapamycin experiment does? Although Rab5DN presumably interferes with endogenous Rab5 function, endogenous Rab5 protein is still present and may complicate the interpretation of lc3b mRNA localization, which may not rely on its GTPase function.

We thank the reviewer for this comment. To investigate the effect of RAB5WT and RAB5DN on endosomal distribution, we performed immunocytochemistry experiments using EEA1 as

a marker of early endosomes (EEs). Our results show that expression of RAB5WT increases the density of the EEA1 signal, whereas expression of RAB5DN produces the opposite effect. These findings are consistent with previous studies demonstrating that modulation of RAB5 GTPase activity affects early endosome biogenesis and distribution. These results are now presented in Figure EV3A and B.

Importantly, these changes in early endosome abundance do not affect the distribution of *lc3b* mRNA (Figure EV3D), supporting the idea that this transcript is broadly distributed throughout the cell independently of RAB5 activity and early endosome trafficking. This conclusion is further reinforced by new live-imaging experiments using the MS2 system, which reveal associations between *lc3b* mRNA and oscillating/static RAB5-positive endosomes, without evidence of coordinated long-range directed movement (Figure 7D). Together, these findings support that early endosomes function as platforms for transient interaction rather than as transport units for the transcript.

• Also, does overexpression of Rab5wt alter the cellular distribution pattern of lc3b mRNA?

This is an important point, as overexpression of RAB5 leads to an increased number of EEA1-positive endosomes in cells (Figure EV3A and B) and could therefore potentially affect *lc3b* mRNA distribution. However, analysis of *lc3b* mRNA localization in cells overexpressing RAB5WT revealed no significant changes (Figure EV3D). These results support that a homogeneous increase in early endosome abundance does not alter *lc3b* mRNA localization.

• To confirm findings of TEM, it would be nice to have an IF staining of autophagosomes compared to the abundance of LC3B. If LC3B is really localized to structures different from APs, this should also show up in IF analysis.

As noted by the reviewer, our TEM analyses support the conclusion that the majority of LC3B localizes to double-membrane autophagosomal structures in axons, whereas a smaller fraction associates with single-membrane vesicles. Although our study provides, to our knowledge, the first evidence of this phenomenon in neurons, and specifically in axons, LC3B localization to single-membrane compartments has previously been described in other cell types, where it has been linked to non-canonical autophagy-related processes such as LC3-associated phagocytosis (LAP).

We agree with the reviewer that the inclusion of additional markers for autophagosomes, particularly those that would allow their exclusive identification, would be highly valuable to accurately quantify the relative distribution of LC3B on these compartments. However, to our knowledge, no currently available marker allows the unequivocal discrimination of canonical autophagosomes from other LC3-positive vesicular structures.

• The idea that locally translated lc3b directly decorates damaged endosomes is very intriguing. Could this approach also be used by other organelles? Is there an colocalization of lc3b mRNA/translation to mitochondria or lysosomes, potentially even after treatment with compounds that damage these organelles (CCCP, Choloroquine!?) Both possibilities are interesting, either this being an endosome specific pathway or a generalizable concept to enable autophagy in axons.

We agree with the reviewer that this is a very exciting question. To address whether *lc3b* mRNA recruitment in response to damage represents a generalizable process across different organelles, we performed a series of experiments analyzing organelle-mRNA association over time by smFISH in SH-SY5Y cells.

Specifically, we used chloroquine to perturb endosomal compartments, LLOMe to disrupt lysosomes, and oligomycin A plus antimycin A (O+A) to impair mitochondrial function. Remarkably, we observed a significant increase in *lc3b* mRNA colocalization with the corresponding damaged organelles across all conditions after 20 minutes of treatment. These findings suggest that *lc3b* mRNA recruitment represents a rapid and dynamic cellular response to organelle damage that is not restricted to early endosomes, but may instead reflect a broader mechanism to locally supply autophagy components at sites of damage. It is important to note that the molecular mechanisms governing *lc3b* mRNA recruitment may vary depending on the organelle involved and the type of damage/stress incurred. Furthermore, our findings raise the possibility that locally synthesized LC3B protein could be incorporated not only into nascent autophagosomes but also directly into damaged membranes. Addressing these possibilities across all organelles and damage contexts would require extensive investigation and falls beyond the scope of this study.

We thank the reviewer for this insightful suggestion. These new results have been included in the revised manuscript (Figure 7A, 7B, 7G, 7H, 7I, 7J).

• Overexpressing ATG7 (C572S) blocks ATG12-ATG5 conjugation and thus autophagy in general. Similarly, chloroquine (CHQ) inhibits autophagy as it prevents fusion of autophagosomes with lysosomes. It is therefore not surprising to find more/damaged Rab5-endosomes in axons in both conditions. Yet what happens to lc3b mRNA and its local translation? Is the mRNA still present and does translation still occur in ATG7 mutant cells, but fails to target endolysosomes?

We agree with the reviewer that investigating whether LC3B axonal translation still occur in presence of ATG7 (C572S) mutant is an interesting point. We therefore expressed ATG7mutant in axons and perform Puro-PLA experiments to test if *lc3b* mRNA axonal translation was affected.

Results revealed no significant change in LC3B axonal synthesis rate in axons expressing ATG7 (C572S) compared to control, supporting no alterations in axonal mRNA localization and translation but a dominant effect of the construct specifically on LC3B lipidation. These results are now presented in Figure EV5.

Minor points

• Please maintain consistency in how you position the labels in the figure legends, ensuring they are placed uniformly either before or after the corresponding description.

We thank the reviewer for this comment. We have revised all figure legends to ensure consistent placement of labels, which are now uniformly positioned relative to their corresponding descriptions.

• The representative colors for the immunofluorescence are very confusing. Single channel images should stay in one color (either white, green, or red), 2-channel images should also stay in 2 colors (e.g. magenta-green to make this manuscript also accessible for red-green color blindness). Especially within a figure, the two channels for colors should remain the same (e.g. figure 2A).

We agree with the reviewer that the original color scheme was confusing. Accordingly, we have revised the manuscript to ensure consistent color usage throughout all figures.

• Line 129-130: The authors write "streptavidin-based immunoprecipititation". Since streptavidin is a protein not an antibody, it is rather only a precipitation or pulldown.

We apologize for this mistake and have corrected the text to read "streptavidin-based pulldown."

• Line 167-169: Please indicate a short statement, why eea1 was used (as the authors did for lc3b RNA).

A short statement has now been added to the manusciprt to better explain why *eea1* was used. It now reads: "*EEA1 (Early Endosome Antigen 1) is a RAB5 effector that regulates EE tethering and fusion, and its encoding mRNA has previously been reported to localize to EEs (Popovic et al., 2020) and was identified in our APEX2-RAB5A condition. We therefore used it as a positive control and quantified its colocalization with RAB5-positive vesicles (Fig. 2A, B).*"

• Fig. 2A should be separated in 2 visually separate panels to emphasize the distinct data:
o Fig 2A: colocalization between eea1, lc3, sqstm1 RNA and Rab5.
o Fig 2B: colocalization between lc3 and eea1 and Rab7 (early vs. late endosomes)

We thank the reviewer for this suggestion. Figures 2A and 2B have been reorganized as recommended to improve clarity.

• Fig. 2F: please indicate what CT abbreviates.

CT abbreviates for Control condition. This clarification has been added to the figure legends.

• Figure 2F: lc3 and sqstm1 should have same number of experiments and comparable numbers of cells. Please add a 3rd replicate to lc3 localization.

We performed additional experiments to obtain a third replicate for testing the effect of RAB5-endosome relocalization on *lc3b* mRNA distribution, and thus a comparable amount of cells per condition. The results have been updated inside the figure.

• Figure 2G,H: please have equal no. of experiments

We decided here to focus on *lc3b* mRNA axonal localization, as it represents the main focus of the study.

• In all plots it should be indicated whether results are non-significant or if there has not been a statistical analysis. Please add the significance bars for figures 2F,H,I,L; 3B,C,D,G; 4B; 5E; EV3A,B; EV4.

We apologize to the reviewer for this oversight and have now added all significance bars to the corresponding figures.

• Please include a reference image for fig. 2L, where its visible where RAB5 localizes with LC3 mRNA

A representative example has been added (now in Figure 2K) to illustrate the results presented in Figure 2L and Figure 2M. Arrows indicate the association of mRNAs with the RAB5 signal.

• The kymograph of figure 3A looks like its pieced together (left side). The authors should make sure it is one graph with the same settings.

We apologize for this oversight. The kymograph was originally generated from a single time-lapse sequence and we don't understnd why it appeared as "cut" and pieced back together. We have now regenerated a new one from the same axon and replaced the previous version accordingly.

• Fig. 3C: please indicate what A.R. means. (Potentially, the graph should be rearranged so that all Rs are grouped together and compared and all As are.)

We apologize for not having mentioned this information in the figure legends. "A" denotes anterograde and "R" denotes retrograde transport. This has now been corrected and data are presented as suggested.

• Please indicate in a short statement what scfvGFP is used for.

We have expanded the description of the SunTag imaging system in the manuscript, including clarification of the role of scFv-GFP. This information has been incorporated into the text. We have also revised the figure labeling to make it clearer that the signal corresponds to nascent SunTag-LC3B protein.

• Fig 3E: Please include a reference picture of a harringtonine treated axon.

A representative example has been added.

• Fig 3F: Please include the lc3b Suntag signal in the image. Also, please include what the arrows indicate.

We apologize if this point was not sufficiently clear. The lc3b SunTag signal, reflecting the event of translation, correspond to the green signal in the image. The legend was confusing. The signal for the mRNA is not visible because the mRNA itself was not labeled. We have added a clearer explanation in both the main text and the figure legends. In addition, an arrow highlights the positive signal corresponding to a nascent SunTag-LC3B protein, and this is now explicitly clarified in the figure legends.

• Line 272: "endosomes could be locally targeted by LC3B in axons". The context of this statement is missing. Targeted by LC3B for what? Please elaborate.

The original statement lacked precision and has been revised to: "*endosomes in axons could be locally regulated by LC3B-mediated autophagy.*"

• Fig 4A: a zoom in would be helpful to assess colocalization better.

The image in Figure 4A has been changed with colors allowing to better visualize colocalization. Moreover, a higher magnification has been added.

• Fig 4B: what does M1 and M2 refer to? Please indicate.

We apologize for the lack of clarity. M1 refers to the fraction of the LC3B signal overlapping with RAB5, while M2 refers to the fraction of the RAB5 signal overlapping with LC3B. This information has now been added to Figure 4B.

• Line 286-287: "autophagy is not limited to ER and mitochondria in developing axons". Please elaborate this statement or add a reference who claimed that autophagy in developing axons was limited in that way.

We acknowledge that the original statement was inaccurate and misleading. Our intention was to emphasize that autophagy of the endoplasmic reticulum and mitochondria in axons has been well studied and established, whereas its role in the regulation of other organelles in axons remains less understood. This statement has now been removed from the manuscript.

• Line 298: The text discusses ATG12-ATG5 conjugation, whereas Figure 4D depicts LC3B lipidation. Please adjust the wording to ensure consistency between the text and the figure content.

We apologize for the lack of clarity. The text has been revised and now reads: "*To block LC3B function in RGC axons, we expressed a mutated form of ATG7 (C572S), which has been shown to block ATG12-ATG5 conjugation and thereby impair LC3B lipidation and membrane association (Nitta et al., 2019, Tanida et al., 2001) (Fig. 4D)*.".

• It is not clear what data was used for fig 4E. If the same images were used as for fig. 4F, fig. 4E should be part of fig. 4F.

The data previously presented in Fig. 4E have now been moved to Fig. 4G. They represent the quantification of the immunofluorescent LC3B signal in axons, as now shown in Fig. 4F. We thank the reviewer for this suggestion, which has improved the clarity of the Figure.

• Fig 5B, C: in the manuscript it is BSA-647 in the figure it is BSA-657. Please correct.

BSA-647 was used for this experiment. This labeling error has now been corrected.

• Fig 5H: Colocalization is hard to detect with the colors magenta, green, red. I would suggest cyan, magenta, yellow or other more distinct colors.

To improve clarity, the colors have been changed in cyan, magenta, yellow as suggested.

• Fig 6D: since CHQ affects autophagy, LC3-GFP should also be shown in this panel.

We agree with the reviewer that CHQ could have potentially increased GFP-LC3B levels in axons. To address this, we initially quantified GFP-LC3B puncta following 30 minutes of stimulation in axons, and did not observe any significant change compared to control conditions. We speculate that this may be due to a combination of factors: (i) a very high variability in the number of GFP-LC3B puncta between axons (see Figure EV6), (ii) a lack of CHQ effect on canonical AP biogenesis in the growth cone; (iii) already elevated baseline levels of GFP-LC3B resulting from RFP-RAB5 overexpression; and (iv) an effect restricted to a limited subset of early endosomes (EEs) along the axon (stressed), making it difficult to detect a global change. Indeed, it is important to note that CHQ is known to significantly increase GFP-LC3B levels in cells primarily by blocking lysosomal degradation of autophagosomes (APs), rather than by enhancing AP biogenesis. In neurons, this effect would be expected to become more apparent in the cell body following longer treatment durations.

This prompted us to perform live-imaging experiments, focusing on RFP-RAB5-positive endosomes that appeared to swell in response to CHQ along the axon shaft. We observed that this swelling was followed by, or occurred concomitantly with, the accumulation of GFP-LC3B on these organelles, as shown in Figure 6E.

• Line 420: please elaborate: "Local regulation" of/for what?

We thank the reviewer for this comment. In response, we expanded the relevant section in the Discussion, which now reads: *"acting as a molecular switch that promotes rapid local endosomal turnover. A similar principle has been described in non-canonical autophagy pathways such as LC3-associated phagocytosis (LAP), where LC3 conjugation onto single-membrane endocytic compartments promotes their maturation and fusion with lysosomes."* We hope that this revision better clarifies our interpretation.

• Fig. EV1 A: x-axis labelling would be helpful

A x-axis labelling has been added to Figure EV1A.

• Fig. EV1 B: -H2O2 samples should have a similar color pattern (e.g. cold colors) and the +H2O2 should have also a similar one (e.g. warm colors) to help distinguishing the samples

As suggested by the reviewer, we have modified Figure EV1B to improve the visual distinction between -H_2O_2 and +H_2O_2 conditions by implementing a consistent color-coding scheme. Specifically, each condition is represented by a distinct color palette, with pastel tones used for -H_2O_2 samples and darker tones for +H_2O_2 samples. We hope that this revision enhances clarity and readability.

• Fig. EV2A: It would be helpful to mention in the text that figure EV2 depicts the schematic of the Boyden chamber.

It is now mentioned in the text and reads: "*Analysis of previously generated axonal RNA-seq data obtained using compartmentalized chambers (Boyden chambers, Fig. EV3A), revealed the presence of several autophagy-related mRNAs, including some identified in our APEXseq (Fig. EV3B).*"

• Fig EV4: attached is a quantification that includes quantification of colocalization of ring-like Rab5 and sqstm1. Therefore, reference pictures of ring-like rab5 colocalizing with sqstm1 should be included in the image as well.

We have added a representative image in Figure EV7 showing ring-like RAB5 vesicle colocalizing with GFP-SQSTM1.

Referee #2:

In this manuscript the authors study the association of mRNAs with early endosomes in axons. Using proximity labeling in a cell culture model system they were able to identify numerous mRNAs that are associated with endosomes. Choosing lc3b mRNA as an example, they reveal a novel mechanistic link to autophagy. The author provide evidence showing that transport of lc3b mRNA and endosomal localization are two distinct processes, suggesting that mRNAs are transported towards early endosomes. Studying translation in vivo revealed local protein synthesis on the surface of endosomes functions most likely to target the translation product to the surface of endosomes. Since LCB3 protein is involved in autophagy, the authors hypothesis that LCB3 might be important for local degradation of endosomes by autophagy. To support this hypothesis the authors demonstrate that inhibition of LC3B by preventing its lipidation results in enlarged endosomes in developing axons. Furthermore, overexpression of the endosomal GTPase RAB5 resulted in larger endosomes and interestingly, lcb3 mRNA and protein accumulate at growing endosomes. Hence, local translation might be important to target dysfunctional endosomes. Finally, they demonstrate that stress treatment using chloroquine alters endosomal homeostasis and this correlates with the accumulation of LC3B most likely by local translation.
In summary, the authors combine a number of sophisticated in vivo approaches such as proximity labeling, life cell imaging of mRNA localization and translation as well as EM imaging. They compare cell culture models with growing vertebrate axons to demonstrate

that the autophagy component LCB3 localize to stressed endosomes. They provide compelling evidence for a new mechanistic link of endosome-coupled translation and quality control by autophagy. Thus, the study merits publication in EMBO reports. However, prior to publication I would like to suggest some improvements.

We thank the reviewer for the positive feedback and helpful suggestions. Accordingly, we carried out additional experiments and revised the manuscript to clarify both the text and the figures.

Major points:
1. Since LC3B and autophagy is a main focus of the manuscript, it would be very helpful to include the state-of-the art on LC3B in the introduction of the manuscript.

We thank the reviewer for this valuable suggestion. We submitted our manuscript as a report, which is subject to strict character limitations, and this constrained the extent to which we could elaborate on LC3B and the autophagy pathway. To address this point, we have now expanded the manuscript by including a more detailed description of LC3B and SQSTM1 and their role in the autophagy process. This information has been incorporated into the Results section (Page 5, from line 177), where it directly supports the interpretation of our findings. We believe this addition improves the overall clarity and provides the necessary context for readers.

2. I had difficulties to understand the rational that mRNA co-localization was verified by rapalog experiment targeting motile endosomes, whereas later it is shown that mRNA motility is independent of endosomes. Please, explain the rational of these experimental series in more detail.

We apologize for the lack of clarity. The observed colocalization between *lc3b* mRNA and RAB5-positive endosomes raised an important question: could this reflect a direct association of the transcript with the organelle?

To address this, we employed the rapalog-based system. This approach was inspired by Popovitch et al., who demonstrated that repositioning RAB5 endosomes to the cell periphery, by inducing an interaction between RAB5 and a kinesin motor protein, resulted in the relocalization of *eea1* mRNA along with the endosomes.

Although *lc3b* mRNA association with RAB5 endosomes appeared less frequent compared to *eea1* transcript, we observed that RAB5–*lc3b* mRNA colocalization was more frequent at the cell periphery. Based on this, we hypothesized that artificially relocalizing RAB5 endosomes toward the cell center, by promoting their interaction with dynein, could alter the spatial distribution of *lc3b* mRNA. Indeed, if *lc3b* mRNA directly associates with RAB5 endosomes, repositioning the organelles should result in the coordinated relocalization of the transcripts present on the surface of the organelle. Consistent with this hypothesis, our experimental results showed that *lc3b* mRNA distribution shifted in accordance with endosome repositioning.

In this scenario, *lc3b* mRNA would co-move as a consequence of the forced relocalization of RAB5 endosomes. However, an alternative explanation must also be considered. We cannot exclude the possibility that relocalizing RAB5 endosomes to the cell center artificially increases the number of available docking sites for the transcript in that region, thereby producing a similar redistribution. This alternative explanation is now cited in the manuscript.

Importantly, this does not imply that endosomes are the primary drivers of mRNA trafficking within the cell. Indeed, the observed reditribution would also be observed if endosomes function predominantly as subcellular docking platforms rather than as active transport units, an hypothesis that is now further supported by our new live-imaging experiments using the MS2 system presented in Figure 7.

Minor points:

1. For consistence, I would recommend to call the constructs: APEX2-RAB5A, APEX2-NES and APEX2-FYVE2 instead of 2xFYVE-APEX2.

We thank the reviewer for this suggestion and agree that consistent nomenclature is important for clarity. However, we chose to retain the designation "2xFYVE-APEX2" to accurately reflect the orientation of the construct, in which the 2xFYVE domain is positioned at the N-terminus and APEX2 at the C-terminus. Renaming it as "APEX2-FYVE2" would invert this order and could therefore be misleading with respect to the actual domain organization.

To ensure clarity, we have clearly defined the construct upon first mention and have used the same terminology consistently throughout the manuscript.

2. Line 133: "A differential expression analysis..". The term "differential expression" is misleading, because the expression did not change.

We thank the reviewer for this comment. We have changed the text from "A differential expression analysis.." to "*A comparative analysis was then performed to identify mRNAs enriched in biotinylated samples relative to all unbiotinylated samples combined*"

3. Line 146: For the GO analysis the overlap of APEX2-RaB5A and 2xFYVE-APEX2 is most important. Focus on the 898 mRNAs that were found in both datasets.

We agree with the reviewer that the overlap between the APEX2-RAB5A and 2xFYVE-APEX2 datasets represents a higher-confidence set of transcripts associated with early endosomes. We also believe that analyzing all conditions remains informative. APEX2-RAB5A and 2xFYVE-APEX2 label partially overlapping but not perfectly identical endosomal subpopulations. Therefore, transcripts identified uniquely in each condition may reflect biologically meaningful heterogeneity in endosomal identity and dynamics. These may correspond to regulated or context-dependent interactions that would be overlooked by focusing exclusively on the intersection. For example, the validated *eea1* mRNA was identified only in the APEX2-RAB5A condition, potentially reflecting a transient or dynamic association with the organelle.

That said, we again agree with the reviewer that the overlapping set of transcripts identified by both APEX2-RAB5A and 2xFYVE-APEX2 is most likely to represent mRNAs closely associated with endosomal membranes. Accordingly, we have improved the description of the Gene Ontology (GO) terms associated with this overlap in the revised manuscript. This consideration also guided our decision to focus on *lc3b* mRNA, as it is present within this intersecting set.

4. Line 158: It is unclear how the "stress response mechanism" was identified.

The reviewer is correct that the proposed “stress response mechanism” was not sufficiently supported by the data presented in the figure. We have therefore revised the text to more accurately reflect our findings and now emphasize, in addition to autophagy-related processes, the enrichment of transcripts encoding regulators of lipid metabolism and membrane-remodeling factors.

5. Line 169: well known autophagy regulator LC3B and SQSTM1 are used but the proteins are not introduced. More details and citations are missing (see Major point 1).

We thank the reviewer for this comment. Our work has now been submitted as an article, allowing us to expand the manuscript and provide a clearer introduction of LC3B and SQSTM1 proteins, including a more detailed description of their established roles in the autophagy pathway, along with appropriate references. We believe that these additions improve the accessibility of the text and provide better context for the reader.

6. Line 226: lc3a is mentioned but not introduced.

The protein LC3A is now brieftly intoduced inside the manuscript: *“lc3a mRNA, encoding another member of the ATG8/LC3 family with overlapping but not identical functions to LC3B in autophagy,”*

7. Line 320: Introduce BSA-647 as labeling tool.

Bovine serum albumin conjugated with Alexa Fluor 647 (BSA-647) was used to visualize endosomal compartments in axons. Following uptake via endocytosis, the fluorescently labeled BSA accumulates within endosomes, enabling their detection and tracking by fluorescence microscopy. This explanation is now present in the manuscript (Page 11, line 371).

8. Line 411: please compare the results on the coding region to the study by the Pelham lab

We have expanded the Discussion, including our new results, to better highlight the differences between our findings and previous studies. Previous work demonstrated that the coding sequence of *Eea1* mRNA is sufficient to mediate its localization to early endosomes and that this association is disrupted by puromycin treatment, supporting a translation-dependent tethering mechanism in which the nascent EEA1 polypeptide contributes to the

interaction between the translating ribosome-mRNA complex and the endosomal membrane.

In contrast, our results show that the coding region of *lc3b* mRNA is also sufficient to promote endosomal localization, yet this association remains resistant to puromycin treatment. These findings suggest that distinct mechanisms underlie the endosomal targeting of these transcripts. In the case of *lc3b* mRNA, localization may rely on RNA-binding proteins and translation-independent recognition of specific sequence or structural features within the coding region, such as stem-loop structures or RNA duplexes. Although less common than 3'UTR-mediated targeting, coding sequences have been shown to serve as platforms for RNA-binding proteins involved in mRNA trafficking and subcellular localization, including STAUFEN1 (Sugimoto et al., 2015) and IGF2BP1 (Conway et al., 2016). It is also important to keep in mind that while our data indicate that the coding region is sufficient to support endosomal association under the conditions tested, they do not exclude additional contributions from the 3'UTR in other cellular contexts or regulatory states.

9. Discussion: provide an overview on the endosome-associated translation: which examples are known and what are the various functions.

The discussion has been expanded in the revised manuscript, including an improved description of endosome-associated translation.

10. A graphical model would be helpful

A graphical hypothetical model has been added to the revised manuscript to illustrate our main findings.

Final points: Could the authors comment on the aspect that the H2O2 treatment during APEX2 labeling might trigger a stress response in the cells and therefore stressed endosomes are also part of the analysis. This would nicely explain the detection of LCB3 mRNA.

We agree with the reviewer that this effect may occur. H_2O_2 is a well-established inducer of cellular stress and, particularly upon prolonged exposure, can affect the integrity of membranes across multiple organelles, including endosomes. The treatment may therefore have favored the association between early endosomes (EEs) and *lc3b* mRNA. This possibility is further supported by our new findings, which show that the EE-*lc3b* mRNA association is increased in response to stress. However, it is important to note that *in situ* experiments validating the association between RAB5 and *lc3b* mRNA were performed in untreated cells, confirming that this interaction can neverthelss occur in the absence of induced stress.

Referee #3:

The manuscript of Pappacena et al. presents a very intriguing research study, which identifies the pool of RNAs localized at endosomes and further shows that the mRNA lc3b is translated within neurons in SH-SY5Y neuronal cell culture and the RGCs of Xenopus. They took an APEX-seq approach to biotinylate RNAs close to the endosome using the FYVE

domain and Rab5 as markers. They identified several RNAs involved in endocytosis, endosomal trafficking, neuronal processes, synaptic environment, and autophagy and stress response pathways. Authors then identified lc3b was locally being translated at endosomes, which may be needed for endosomal autophagy. Endosomal stressors such as Rab5 overexpression and chloroquine lead to previously described large endosomal rings, which the authors have found actively translate LC3B until structures are encased. This is a very exciting work that identifies a novel non-conventional autophagic pathway that maintains endosomal integrity through local translation. I would support the publication of this manuscript after the following minor concerns are addressed.

We really appreciate the reviewer's constructive comments and valuable suggestions. In response, we performed additional experiments and revised the manuscript to improve the clarity of both the text and the figures.

In Figure 5 B, C, the authors show that LC3b-positive vesicles partially colocalize with endocytosed BSA647 in the steady states, and the colocalization increased upon Rab5 overexpression. Together with ATG7 dominant mutants' data (Figure 4), the authors interpret these results as endocytic vesicles being constitutively targeted for autophagy through de novo LC3 lipidation on the Rab5-positive vesicle's membrane. However, an alternative interpretation could be, the synthesized LC3 protein incorporates into the nascent autophagosome, followed by fusion with Rab5-positive vesicles. Similar to the atypical autophagy pathway LC3-endocytic-associated-pathway (LEAP), which shows a similar phenotype of LC3 colocalization with Rab5 endosomes (Coelho et al 2022 Nat Comm).

We thank the reviewer for this important comment. As noted, we believe we have obtained evidence that RAB5-positive endosomes are regulated by LC3-dependent autophagy processes in axons. As the reviewer highlighted, this conclusion is supported by several observations, including the increased number and size of RAB5-positive endosomes in axons expressing a dominant-negative ATG7 mutant, as well as the increased colocalization between RAB5 and LC3B in axons overexpressing RAB5 or treated with CHQ.

As raised by the reviewer, an important question is: how does this regulation occur?

Our results suggest that this process may take place through two distinct mechanisms within axons. First, we observed that RAB5-positive endosomes can be incorporated into nascent autophagosomes (APs) in the distal portion of the axon, including growth cones (as illustrated in Figure 5F). These events are consistent with canonical autophagy. We propose that this mechanism accounts for the majority of the colocalization observed between RAB5 and LC3B along axons, and leads to the presence of RAB5 signal inside the AP lumen as illustrated in Figure 5C, 5D, 5F.

In addition, we also detected instances in which RAB5-positive endosomes harboring *lc3b* mRNA directly become LC3B-positive along the axon shaft (Figure 5G). In this specific case, we think that these do not reflect the inclusion of the organelle into APs but rather the insertion of LC3B directly into the vesicle membrane. This is supported by our results

showing that GFP signal on the RAB5-positive membrane have approximately half of the fluorescent signal present on APs having RAB5 signal in their lumen (Figure 5D), possibly reflecting insertion into single versus double membrane organelle. These results are also supported by our TEM results showing the presence of single membrane LC3B-positive vesicles along the axon shaft (Figure 4C).

To nevertheless further investigate the reviewer's question, we generated a new construct enabling the expression of GFP-DFCP1 in axons. DFCP1 (Double FYVE Domain Containing Protein 1) is an endoplasmic reticulum–resident protein that serves as a key marker of omegasomes, membrane platforms that give rise to nascent autophagosomes in the distal portion of the axon (Maday and Holzbaur, Dev Cell, 2014). Imaging of axons expressing GFP-tagged DFCP1 confirmed the presence of DFCP1-positive autophagosome (AP) initiation sites restricted to distal axons. We directly observed, in real time, the rapid appearance and expansion of DFCP1-positive membranes, a process that occurs within a few minutes and culminates in the disappearance of the signal. Furthermore, co-expression with RFP-RAB5 revealed RAB5-positive endosomal signals colocalizing within the lumen of nascent DFCP1-positive omegasomes in distal axons (see figure below). These results further support our model of canonical autophagy involving RAB5-positive vesicles occurring in distal axons.

However, despite extensive acquisition of time-lapse sequences, we never observed the inclusion of enlarged, ring-like RAB5-positive endosomes into omegasomes along the axon shaft. While it is important to acknowledge that "absence of evidence is not evidence of absence," our observations suggest that such events may be extremely rare, occuring below our detection limits, and support the possibility for direct inclusion of LC3B into the enlarged endosomal membrane along the axon shaft.

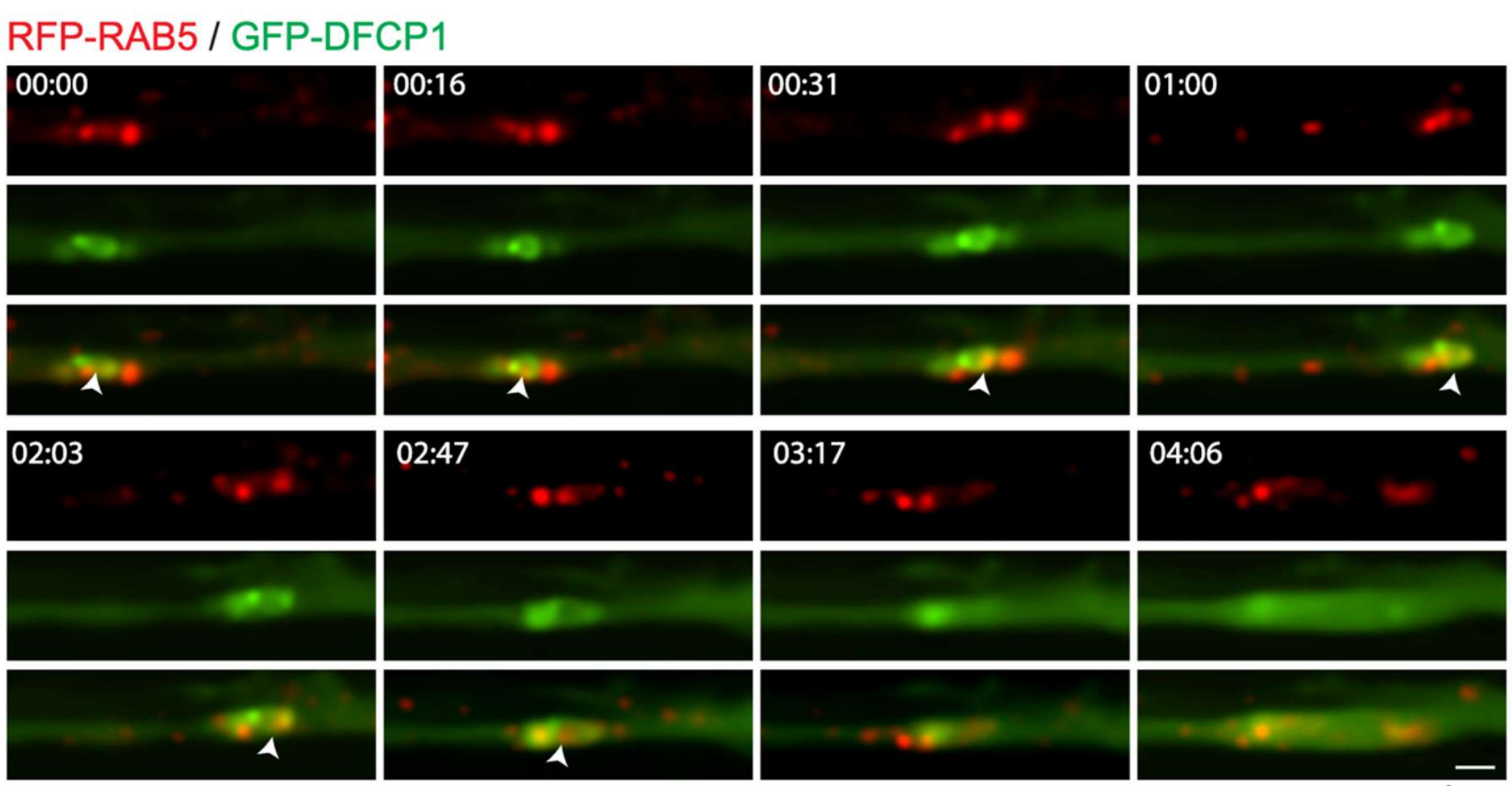

RAB5-positive endosomes are engulfed within GFP-DFCP1-marked omegasomes in distal axons. Time-lapse imaging sequence showing the presence of RFP-RAB5 signal inside the lumen of a nascent autophagosome labeled by the transient accumulation of GFP-DFCP1. Time is indicated as min:sec. Scale bar 1μm.

2) An additional important question indirectly raised by the reviewer's comment is to understand how these newly LC3B-labeled vesicles are subsequently regulated.

Unfortunately, such conversion events were relatively infrequent, and we were unable to perform long-term time-lapse imaging to reliably determine the fate of RAB5-positive vesicles following LC3B insertion into their membrane. Nevertheless, we observed by live-imaging experiments several potential outcomes for LC3B-positive RAB5 vesicles in axons. These include: (i) a subsequent loss of the RAB5 signal, suggesting complete conversion; (ii) disappearance of the vesicle; (iii) fusion between two $RAB5^{+}/LC3B^{+}$ vesicles; and (iv) interactions with other LC3B-positive vesicles.

The latter scenario is consistent with the frequent organelle-organelle interactions observed in RAB5-overexpressing axons and supports the reviewer's suggestion that these structures may ultimately fuse with pre-existing autophagosomes (APs) along the axon shaft and be targeted for degradation. Taken together, these observations support the idea that all these mechanisms coexist and collectively contribute to a reduction in RAB5-positive vesicles in axons.

However, as these observations do not allow us to draw definitive quantitative or mechanistic conclusions, we have chosen not to include them in the manuscript.

Nevertheless, we believe that our findings provide important insight into the interplay between endosomal trafficking and autophagy in axons, and they establish a framework for future studies aimed at elucidating the precise molecular processes regulating the degradation of these LC3B-associated RAB5-positive compartments.

In Figure 2J, K, L, the authors show that the deletion of the 3'-UTR leads to a decrease in the number of lc3b mRNA puncta in the distal axon, but that the association with Rab5 endosomes remains unchanged. Does the number of Rab5 endosomes change in the distal axon? A quantification of this data would also be helpful.

This is an interesting question. We quantified the density of RAB5-labeled vesicles in axons expressing either full-length *lc3b* mRNA or a version lacking the 3'UTR. Our results revealed no significant differences between these conditions.

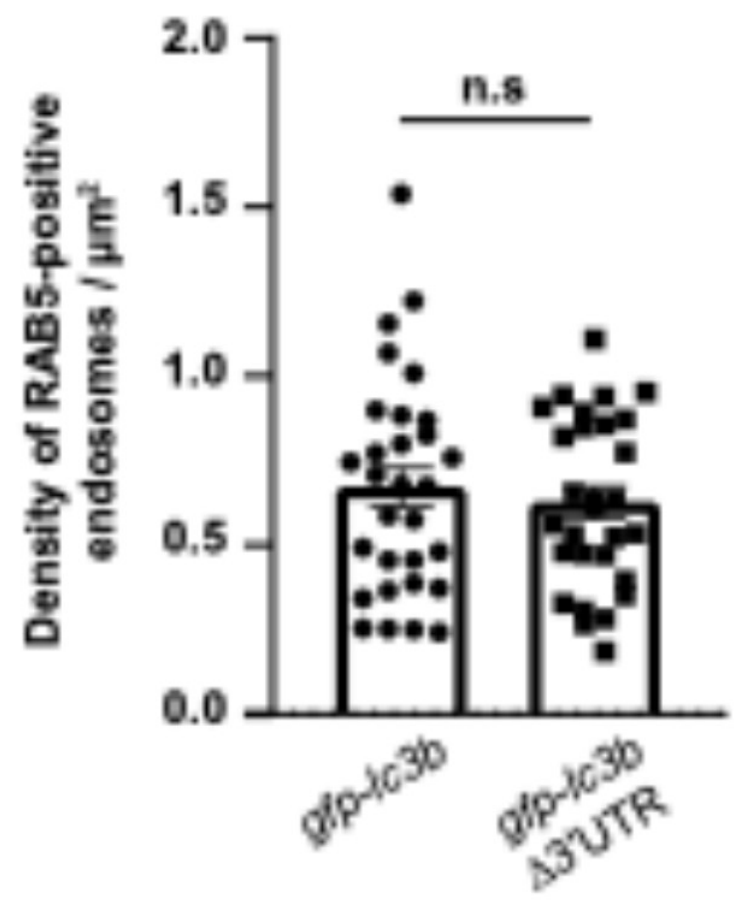

Expression of exogeneous gfp-lc3b mRNA and gfp-lc3bΔ3'UTR mRNAs does not alter RAB5-positive endosome density in axons. Quantification of the density of RFP-RAB5 puncta in axons. Dots represent axons. (*gfp-lc3b mRNA: 30* axons, *gfp-lc3bΔ3'UTR*: 29 axons, 3 exp.). Mann-Whitney test.

It is important to note that, in this assay, we are testing the ability of a fixed and controlled number of *lc3b* mRNA molecules to localize to axons and endosomes. In both conditions, the endogenous mRNA is still present. Therefore, an effect on early endosomes might be expected only in an experimental context where the endogenous lc3b gene is edited to remove the 3'UTR. Such an experiment would provide strong evidence to test the functional role of *lc3b* mRNA in axons. However, and unfortunately, it would be extremely difficult due to significant technical challenges associated with gene editing in *Xenopus laevis* (e.g. complex allotetraploid genome).

Minor suggestions:
Figure 2K-L: Please provide the corresponding images for this quantification.

The quantification previously shown in Figure 2K-L are now in Figure 2L-M and a representative images illustrating these findings has been added in Figure 2K.

Figure 3E - I would suggest adding the analysis of scfvGFP and including an image of harringtonine treatment to create a more complete figure.

We decided to remove the condition of scFvGFP expression alone, as no foci were observed in axons. However, as suggested by the reviewer, we included a representative image of axons treated with harringtonine, showing the decrease in SunTag-LC3B foci.

Figure 3G - An axis label would be beneficial for this graph.

We apologize for this mistake and have corrected the graph present in Figure 3G.

Figure 4B - Indicate what M1 and M2 (i.e., M1 is LC3B) either on the graph or in the legend.

We apologize for any lack of clarity. M1 denotes the proportion of the LC3B signal that overlaps with RAB5, whereas M2 indicates the proportion of the RAB5 signal that overlaps with LC3B. This clarification has now been included in Figure 4B.

Figure 4C - It would be beneficial to have labeling of structures within the TEM images to go with the text descriptions.

We have lightly annotated the structures in the TEM images. However, labeling microtubules proved challenging and initially interfered with the clear visualization of the immunogold signal. We hope that the revised images are now clearer.

Figure 5A - Corresponding images for this graph would be a good addition to the supplement.

The graph initially present in Figure 5A, and the corresponding images, are now present in Figure EV6.

Figure 5B - The dynamics of the LC3B and BSA647 endosomes are noted in the kymograph, but no description or graph of the results is provided.

We added a sentence to the manuscript describing the movement of the colocalizing signals, which predominantly exhibit oscillatory and retrograde co-movement, consistent with a degradative pathway.

Figure 5G - Please provide the video that the stills are from to the supplement. Additionally, add stills between 1 - 10 minutes of the autophagic structure developing; it is not clear that there is an appearance of the ring structure after a single puncta. It would be helpful to see the RFP channel by itself for these stills as well to show that Rab5 is being incorporated into these autophagic compartments.

We agree with the reviewer that it was diffuclt to easily appreciate these cellular events with the images presented in Figure 5G. We have now added the video to the supplementary data and additionnal stills to the presented sequence in the figure. We hope that the data are more clear now showing the sequentiel biogenesis of the AP followed by the presence of RAB5 signal inside APs, supporting local incorporation, and subsequent retrograde transport.

1st Revision - Editorial Decision 12th Jun 2026

Dear Dr. Cioni

Thank you for the submission of your revised manuscript to EMBO reports. We have now received the reports from the referees that were asked to assess it.

As you will see, both referees are very positive about the study and recommend publication. Before I can accept the manuscript, I need you to address some minor points below:

- Your manuscript has now 7 figures and will be published as a full Article. This would also leave room for having a longer introduction and discussion of LC3B there, as referee #2 had suggested.

- The corresponding author's email address is needed on the title page.

- We noticed the following name discrepancies: Caterino Cinzia in the manuscript vs. Cinzia Caterino in the system; Sironi Cristina in the manuscript vs. Cristina Sironi in the system; the manuscript and the manuscript submission system need to have the same format of author names: First Name Last Name. Please correct these names as appropriate.

- SUMMARY should be ABSTRACT

- Materials and Methods should be Methods

- The manuscript sections should be in the following order: Title page - Abstract - Introduction - Results - Discussion - Methods - Data Availability - Acknowledgments - Disclosure Statement & Competing Interests - References - Figure Legends - (Main Tables with legends if applicable) - Expanded View Figure Legends.

- SUPPLEMENTARY FIGURES should be EXPANDED VIEW FIGURE LEGENDS

- Regarding the Author Contributions, we use CRediT to specify the contributions of each author in the journal submission system. CRediT replaces the author contribution section, which therefore needs to be removed from the manuscript text. You can use the free text box in our system if you wish to provide more detailed descriptions. See also guide to authors https://www.embopress.org/page/journal/14693178/authorguide#authorshipguidelines.

- Popovic D et al, 2020 is a preprint. Please cite it in the text as (preprint: Popovic et al., 2020) and add the tag [PREPRINT] at the end of the reference in the reference list.

- References: et al needs to be used after 10 author names.

- Please remove the Instructions from the Reagent and Tools table.

- Source data: I recommend providing the metadata for the fluorescent images shown in Figure 1B. A spot check of the imaging data with Fiji showed that a biotin signal seems present in the APEX2-NES sample if one increases brightness and contrast a lot. Adding information on imaging aquisiton settings would help to document that this is low intensity background signal that would only show e.g., with higher laser power or prolonged exposure.

- Some comments on the figures:
- The scale bars on Figure 6A are very thin and might be hard to see.
- Figure 7A and 7G: worth mentioning that the small images have been rotated relative to the overview image? Figure 7F: the stills from the timelapse have also been rotated, which is not a problem at all, but could maybe be mentioned in the legend for better orientation.
- Figure EV3C: please provide MW markers.
- Figure EV4A: please remove the text "10 um" from the panel.
- Figure EV4C: please also show the individual datapoints in addition to the mean and SEM.
- Please add a callout for Fig. 7FIJ.

- Table EV1 is a complex dataset and needs to be updated to Dataset EV1 in all places (file name and callouts). Please also provide a legend for the Dataset in a separate tab of the .xls file.

- Movie: Please provide a legend and a callout in the manuscript. The legend should be provided in a README.txt file and then both the legend and the movie should be zipped up and uploaded as Movie EV1 zip folder. The callout in the manuscript, source file name, legend title, etc. should be Movie EV1.

- Please provide the synopsis in either jpeg, tiff, png, or eps format. The size is: 550 pixels wide x 300 pixels high. Please make

sure that the text is readable at this small size. Please upload the images as file type Cover Art/Synopsis.

- Please also provide a short (1-2 sentences) summary of the findings and their significance, and 2-3 bullet points highlighting key results. This text will be displayed underneath the synopsis image on the webpage.

- Our production/data editors have asked you to clarify several points in the figure legends (see below). Please incorporate these changes in the manuscript and return the revised file with tracked changes with your final manuscript submission.
- Please note that the exact p values are not provided in the legends of 2B,D,E,L; 3D,E; 4E,G,H,I; 5B,E,H,I,J; 6B,C,D; 7B,E,H,J; EV-4B; EV-6B.
- Please indicate the statistical test used for data analysis in the legends of figures 1D,E,G,H; EV-1C,D; EV-4D.
- Please note that the error bars are not defined in the legends of EV-2B; EV-6B; EV-7B.
- Please note that the white and yellow arrowheads are not defined in the legend of figures 2C,F; 3A,D,E,F; 5A,F,G; EV-7A; This needs to be rectified.

- I introduced a few minor changes in the abstract and suggest a shorter title. Please see the drafts at the end of this e-mail.

We look forward to seeing a final version of your manuscript as soon as possible.

With kind regards,

Martina

Martina Rembold, PhD
Senior Editor
EMBO reports

=================

Referee #1:

The authors have addressed all comments adequately.

Referee #3:

[recommends publication in the summary evaluation sheet of the system]

===============

Local translation of endosome-associated lc3b mRNA in axons contributes to endosomal clearance

RNA localization to organelles is emerging as a key mechanism for regulating protein expression at the subcellular level in neurons. Although certain transcripts associate with endosomes, the functional significance remains poorly understood. Using APEX-seq, we identify a broad set of mRNAs localized to endosomes. We focus on the autophagy-related lc3b mRNA and confirm its endosomal association in cultured cells and Xenopus neuronal axons. In axons, lc3b mRNA is translated at endosomes, where the resulting LC3B protein also colocalizes, suggesting a tight spatial coupling between transcript localization and protein function. Impairment of LC3B membrane insertion via expression of a mutant ATG7 leads to the accumulation of enlarged axonal endosomes. Moreover, RAB5 overactivation promotes the formation of dysfunctional endosomes in axons that are targeted and cleared by LC3B-mediated autophagy. Finally, chloroquine-induced damage to axonal endosomes triggers their targeting by LC3B in a translation-dependent manner. Collectively, our findings expand the catalog of endosome-associated transcripts and reveal a functional link between autophagy and endosomal turnover in axons.

All minor editorial requests have been addressed by the authors.

2nd Revision - Editorial Decision **26th Jun 2026**

Dr. Jean-Michel Cioni
IRCCS San Raffaele Scientific Institute, San Raffaele Hospital
Division of Neuroscience
via Olgettina 60
Milan 20132
Italy

Dear Jean-Michel,

Thank you for implementing the final minor revisions. I am very pleased to accept your manuscript for publication in the next available issue of EMBO reports. Thank you for your contribution to our journal.

Your manuscript will be processed for publication by EMBO Press. It will be copy edited and you will receive page proofs prior to publication. Please note that you will be contacted by Springer Nature Author Services to complete licensing and payment information.

You may qualify for financial assistance for your publication charges - either via a Springer Nature fully open access agreement or an EMBO initiative. Check your eligibility: https://link.springer.com/journal/44319/how-to-publish-with-us

Should you be planning a Press Release on your article, please notify the production team via this form in order to coordinate publication and release dates: https://forms.cloud.microsoft/e/eH4A8JVP6g

If you have any questions, please do not hesitate to contact the Editorial Office. Thank you for your contribution to EMBO Reports.

Best regards,

Martina

Martina Rembold, PhD
Senior Editor
EMBO reports

>>> Please note that it is EMBO Reports policy for the transcript of the editorial process (containing referee reports and your response letter) to be published as an online supplement to each paper. If you do NOT want this, you will need to inform the Editorial Office via email immediately. More information is available here: https://link.springer.com/partners/embo-press/editorial-policies#Peer%20review

EMBO Press Author Checklist

Corresponding Author Name: Jean-Michel Cioni	
Journal Submitted to: EMBO Report	
Manuscript Number: EMBOR-2025-62901V3	

USEFUL LINKS FOR COMPLETING THIS FORM
The EMBO Journal - Author Guidelines
EMBO Reports - Author Guidelines
Molecular Systems Biology - Author Guidelines
EMBO Molecular Medicine - Author Guidelines

Reporting Checklist for Life Science Articles (updated January
This checklist is adapted from Materials Design Analysis Reporting (MDAR) Checklist for Authors. MDAR establishes a minimum set of requirements in transparent reporting in the life sciences (see Statement of Task: 10.31222/osf.io/9sm4x). Please follow the journal's guidelines in preparing your
Please note that a copy of this checklist will be published alongside your article.

Abridged guidelines for figures
1. Data
The data shown in figures should satisfy the following conditions:
- the data were obtained and processed according to the field's best practice and are presented to reflect the results of the experiments in an accurate and unbiased manner.
- ideally, figure panels should include only measurements that are directly comparable to each other and obtained with the same assay.
- plots include clearly labeled error bars for independent experiments and sample sizes. Unless justified, error bars should not be shown for technical
- if n<5, the individual data points from each experiment should be plotted. Any statistical test employed should be justified.
- Source Data should be included to report the data underlying figures according to the guidelines set out in the authorship guidelines on Data

2. Captions
Each figure caption should contain the following information, for each panel where they are relevant:
- a specification of the experimental system investigated (eg cell line, species name).
- the assay(s) and method(s) used to carry out the reported observations and measurements.
- an explicit mention of the biological and chemical entity(ies) that are being measured.
- an explicit mention of the biological and chemical entity(ies) that are altered/varied/perturbed in a controlled manner.
- the exact sample size (n) for each experimental group/condition, given as a number, not a range;
- a description of the sample collection allowing the reader to understand whether the samples represent technical or biological replicates (including how many animals, litters, cultures, etc.).
- a statement of how many times the experiment shown was independently replicated in the laboratory.
- definitions of statistical methods and measures:
  - common tests, such as t-test (please specify whether paired vs. unpaired), simple $\chi 2$ tests, Wilcoxon and Mann-Whitney tests, can be unambiguously identified by name only, but more complex techniques should be described in the methods section;
  - are tests one-sided or two-sided?
  - are there adjustments for multiple comparisons?
  - exact statistical test results, e.g., P values = x but not P values < x;
  - definition of 'center values' as median or average;
  - definition of error bars as s.d. or s.e.m.

Please complete ALL of the questions below.
Select "Not Applicable" only when the requested information is not relevant for your study.

Materials

Newly Created Materials	Information included in the manuscript?	In which section is the information available? (Reagents and Tools Table, Materials and Methods, Figures, Data Availability Section)
New materials and reagents need to be available; do any restrictions apply?	Yes	Reagents and Tools and Materials and Methods

Antibodies	Information included in the manuscript?	In which section is the information available? (Reagents and Tools Table, Materials and Methods, Figures, Data Availability Section)
For **antibodies** provide the following information:<br>- Commercial antibodies: RRID (if possible) or supplier name, catalogue number and or/clone number<br>- Non-commercial: RRID or citation	Yes	Reagents and Tools and Materials and Methods

DNA and RNA sequences	Information included in the manuscript?	In which section is the information available? (Reagents and Tools Table, Materials and Methods, Figures, Data Availability Section)
**Short novel DNA or RNA including primers, probes**: provide the sequences.	Yes	Reagents and Tools and Materials and Methods

Cell materials	Information included in the manuscript?	In which section is the information available? (Reagents and Tools Table, Materials and Methods, Figures, Data Availability Section)
**Cell lines:** Provide species information, strain. Provide accession number in repository **OR** supplier name, catalog number, clone number, and/**OR** RRID.	Yes	Reagents and Tools and Materials and Methods
**Primary cultures:** Provide species, strain, sex of origin, genetic modification status.	Yes	Reagents and Tools and Materials and Methods
Report if the cell lines were recently **authenticated** (e.g., by STR profiling) and tested for mycoplasma contamination.	Not Applicable	

Experimental animals	Information included in the manuscript?	In which section is the information available? (Reagents and Tools Table, Materials and Methods, Figures, Data Availability Section)
**Laboratory animals or Model organisms:** Provide species, strain, sex, age, genetic modification status. Provide accession number in repository **OR** supplier name, catalog number, clone number, **OR** RRID.	Yes	Reagents and Tools and Materials and Methods
**Animal observed in or captured from the field:** Provide species, sex, and age where possible.	Not Applicable	
Please detail **housing and husbandry conditions**.	Not Applicable	

Plants and microbes	Information included in the manuscript?	In which section is the information available? (Reagents and Tools Table, Materials and Methods, Figures, Data Availability Section)
**Plants:** provide species and strain, ecotype and cultivar where relevant, unique accession number if available, and source (including location for collected wild specimens).	Not Applicable	
**Microbes:** provide species and strain, unique accession number if available, and source.	Not Applicable	

Human research participants	Information included in the manuscript?	In which section is the information available? (Reagents and Tools Table, Materials and Methods, Figures, Data Availability Section)
If collected and within the bounds of privacy constraints report on age, sex and gender or ethnicity for all study participants.	Not Applicable	

Core facilities	Information included in the manuscript?	In which section is the information available? (Reagents and Tools Table, Materials and Methods, Figures, Data Availability Section)
If your work benefited from core facilities, was their service mentioned in the acknowledgments section?	Not Applicable	

Design

Study protocol	Information included in the manuscript?	In which section is the information available? (Reagents and Tools Table, Materials and Methods, Figures, Data Availability Section)
If study protocol has been **pre-registered, provide DOI in the manuscript**. For clinical trials, provide the trial registration number **OR** cite DOI.	Not Applicable	
Report the **clinical trial registration number** (at ClinicalTrials.gov or equivalent), where applicable.	Not Applicable	

Laboratory protocol	Information included in the manuscript?	In which section is the information available? (Reagents and Tools Table, Materials and Methods, Figures, Data Availability Section)
Provide DOI OR other citation details if **external detailed step-by-step protocols** are available.	Yes	Materials and Methods

Experimental study design and statistics	Information included in the manuscript?	In which section is the information available? (Reagents and Tools Table, Materials and Methods, Figures, Data Availability Section)
Include a statement about **sample size** estimate even if no statistical methods were used.	Yes	Figure legends
Were any steps taken to minimize the effects of subjective bias when allocating animals/samples to treatment (e.g. **randomization procedure**)? If yes, have they been described?	Yes	Materials and Methods
Include a statement about **blinding** even if no blinding was done.	Yes	Materials and Methods
Describe **inclusion/exclusion criteria** if samples or animals were excluded from the analysis. Were the criteria pre-established?<br><br>If sample or data points were omitted from analysis, report if this was due to attrition or intentional exclusion and provide justification.	Not Applicable	
For every figure, are **statistical tests** justified as appropriate? Do the data meet the assumptions of the tests (e.g., normal distribution)? Describe any methods used to assess it. Is there an estimate of variation within each group of data? Is the variance similar between the groups that are being statistically compared?	Yes	Materials and Methods and Figure legends

Sample definition and in-laboratory replication	Information included in the manuscript?	In which section is the information available? (Reagents and Tools Table, Materials and Methods, Figures, Data Availability Section)
In the figure legends: state number of times the experiment was **replicated** in laboratory.	Yes	Figure legends
In the figure legends: define whether data describe **technical or biological replicates**.	Yes	Figure legends

Ethics

Ethics	Information included in the manuscript?	In which section is the information available? (Reagents and Tools Table, Materials and Methods, Figures, Data Availability Section)
Studies involving **human participants**: State details of **authority granting ethics approval** (IRB or equivalent committee(s), provide reference number for approval.	Not Applicable	
Studies involving **human participants**: Include a statement confirming that **informed consent** was obtained from all subjects and that the experiments conformed to the principles set out in the WMA Declaration of Helsinki and the Department of Health and Human Services Belmont Report.	Not Applicable	
Studies involving **human participants:** For publication of **patient photos**, include a statement confirming that consent to publish was obtained.	Not Applicable	
Studies involving experimental **animals**: State details of **authority granting ethics approval** (IRB or equivalent committee(s), provide reference number for approval. Include a statement of compliance with ethical regulations.	Yes	Materials and Methods
Studies involving **specimen and field samples:** State if relevant **permits** obtained, provide details of authority approving study; if none were required, explain why.	Not Applicable	

Dual Use Research of Concern (DURC)	Information included in the manuscript?	In which section is the information available? (Reagents and Tools Table, Materials and Methods, Figures, Data Availability Section)
Could your study fall under dual use research restrictions? Please check biosecurity documents and list of **select agents and toxins** (CDC): https://www.selectagents.gov/sat/list.htm	Not Applicable	
If you used a select agent, is the security level of the lab appropriate and reported in the manuscript?	Not Applicable	
If a study is subject to dual use research of concern regulations, is the name of the **authority granting approval and reference number** for the regulatory approval provided in the manuscript?	Not Applicable	

Reporting

The MDAR framework recommends adoption of discipline-specific guidelines, established and endorsed through community initiatives. Journals have their own policy about requiring specific guidelines and recommendations to complement MDAR.

Adherence to community standards	Information included in the manuscript?	In which section is the information available? (Reagents and Tools Table, Materials and Methods, Figures, Data Availability Section)
State if relevant guidelines or checklists (e.g., **ICMJE, MIBBI, ARRIVE, PRISMA**) have been followed or provided.	Not Applicable	
For **tumor marker prognostic studies,** we recommend that you follow the **REMARK** reporting guidelines (see link list at top right). See author guidelines, under 'Reporting Guidelines'. Please confirm you have followed these guidelines.	Not Applicable	
For **phase II and III randomized controlled trials**, please refer to the **CONSORT** flow diagram (see link list at top right) and submit the CONSORT checklist (see link list at top right) with your submission. See author guidelines, under 'Reporting Guidelines'. Please confirm you have submitted this list.	Not Applicable	

Data Availability

Data availability	Information included in the manuscript?	In which section is the information available? (Reagents and Tools Table, Materials and Methods, Figures, Data Availability Section)
Have **primary datasets** been deposited according to the journal's guidelines (see 'Data Deposition' section) and the respective accession numbers provided in the Data Availability Section?	Yes	Materials and Methods
Were **human clinical and genomic datasets** deposited in a public access-controlled repository in accordance to ethical obligations to the patients and to the applicable consent agreement?	Not Applicable	
Are **computational models** that are central and integral to a study available without restrictions in a machine-readable form? Were the relevant accession numbers or links provided?	Not Applicable	
If publicly available data were reused, provide the respective **data citations in the reference list**.	Yes	Manuscript
