# Supplementary material for: Local translation of endosome-associated lc3b mRNA in axons contributes to endosomal clearance
Source: EMBO Rep. 2026 Jul 17;27(18):5233–61. doi: 10.1038/s44319-026-00867-5 (PMC13601600; doi:10.1038/s44319-026-00867-5)
Supplement: Supplementary file 5 [file 44319_2026_867_MOESM4_ESM.zip › Figure 1/1c/Figure 1c.pdf]

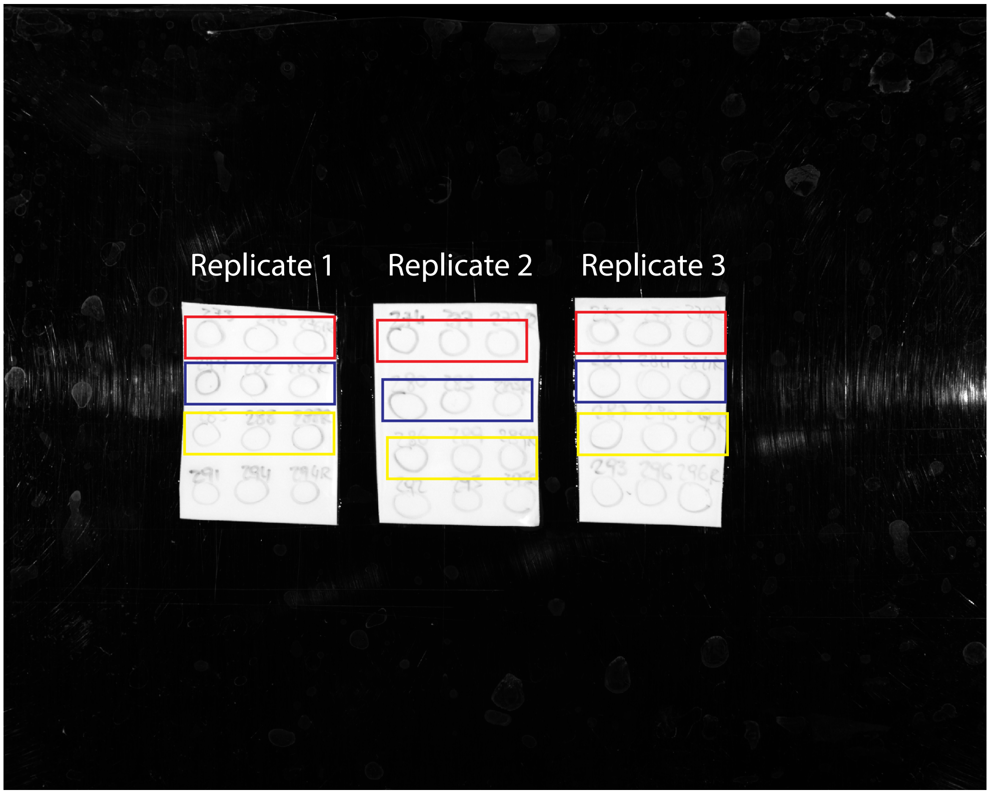

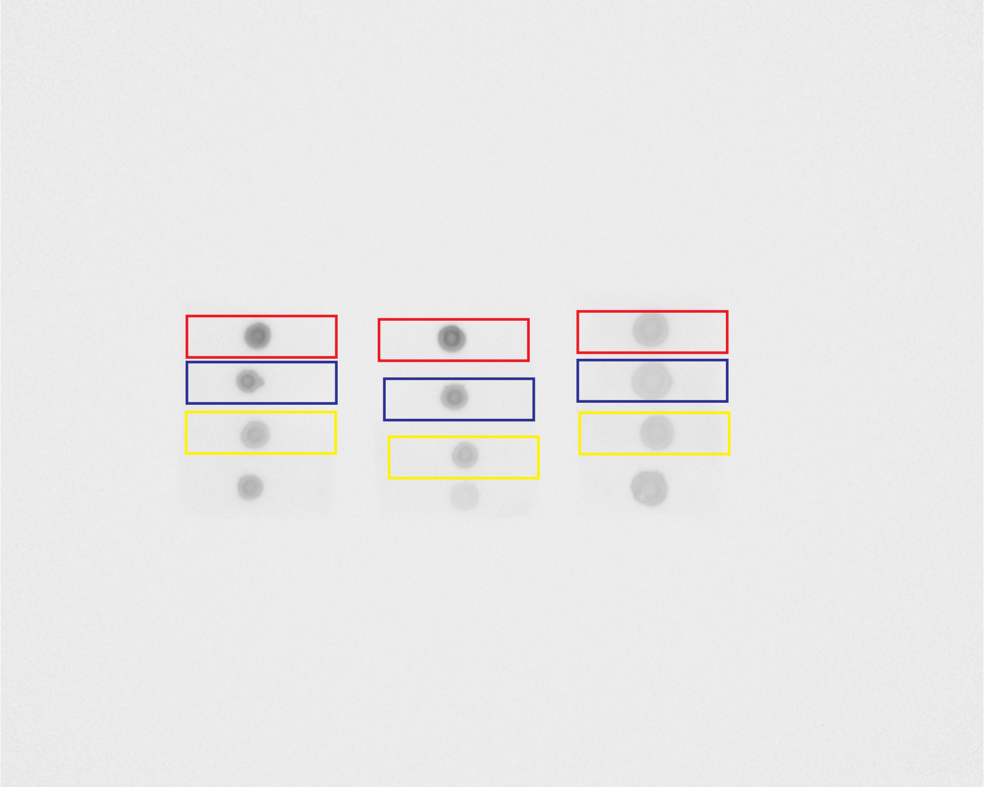

APEX2-NES

2xFYVE-APEX2

APEX2-RAB5A
